# The Effectiveness of Contact Tracing to Reduce Transmission of Infectious Diseases During Epidemic or Pandemic Response: Rapid Systematic Review

**DOI:** 10.2196/84805

**Published:** 2026-03-31

**Authors:** Michael Nunns, Samantha Febrey, Kieran Becker, Morgan Weiland, Jill Buckland, Rebecca Abbott, Rebecca Whear, Alison Bethel, Liz Shaw, Kate Boddy, Serena Carville, Tamsyn Harris, Jo Thompson Coon, G J Melendez-Torres

**Affiliations:** 1 Isca Evidence, University of Exeter Medical School Faculty of Life Sciences University of Exeter Exeter United Kingdom; 2 University of Exeter Medical School NIHR Applied Research Collaboration South West Peninsula (PenARC) University of Exeter Exeter United Kingdom; 3 UK Health Security Agency London United Kingdom

**Keywords:** contact tracing, epidemic response, pandemic preparedness, partner notification, public health and social measures, systematic review

## Abstract

**Background:**

Contact tracing (CT), the process of identifying and managing contacts of infected cases, is one public health and social measure that may reduce the spread of infectious diseases. While previous systematic reviews of CT exist, a comprehensive review of both the effectiveness and potential unintended consequences has not been undertaken to our knowledge. Understanding effective CT strategies could help governments and health authorities prepare effectively for emergency epidemic or pandemic situations.

**Objective:**

This study aims to systematically review the evidence on the effectiveness of CT across infectious diseases with epidemic or pandemic potential. Effectiveness is measured in terms of impacts on disease transmission, health care use, mortality, or unintended consequences.

**Methods:**

We searched 6 bibliographic databases (MEDLINE, Embase, Global Health, CINAHL Ultimate, Cochrane, and Scopus) between November 29 and December 3, 2024, with supplementary citation searching. We sought human studies comparing CT with interventions with no CT or other forms of CT, delivered in the community, in prespecified diseases of epidemic or pandemic potential. We included studies with any measure of disease transmission, related health care use, or unintended consequences of CT and prioritized studies with concurrent comparators. Screening, data extraction, and critical appraisal were performed in duplicate. Due to substantial heterogeneity, a narrative synthesis was performed. This review was informed by meetings with a patient and public involvement and engagement group.

**Results:**

After deduplication, a total of 12,816 titles and abstracts were screened, with 198 records assessed for eligibility at full text. Five additional studies were found through supplementary searching. Finally, 88 reports (of 86 studies) were included, of which 57 reports (of 55 studies) were prioritized. Two main routes of transmission were represented: respiratory (tuberculosis [TB], 15 studies; COVID-19, 5 studies) and blood-borne or sexually transmitted infections (STIs; 35 studies, of which 13 were in HIV, and 22 were bacterial or parasitic infections). No evidence was found on vector-borne, direct contact, or food- or water-borne routes of transmission. Evidence was highly heterogeneous, and more than half of the studies had notable methodological limitations. While there was no difference between CT and comparator interventions for most outcomes, there was some evidence of reductions in disease prevalence in TB and for provider-initiated CT to be superior to patient-led approaches in STIs. Only 2 studies reported measures of unintended consequences.

**Conclusions:**

We found inconsistent evidence for the effectiveness of CT, focused primarily on TB and on contrasts between provider-initiated CT and patient-led referral in STIs and HIV. High heterogeneity in study design precluded clear assertions regarding optimal strategies for CT, including with respect to relevant subgroups. Future work should consider generalizability of CT mechanisms across contexts, including by route of transmission and from the Global South, and a more thorough account of unintended consequences.

## Introduction

### Overview

The term Public Health and Social Measures (PHSMs) describes a range of nonpharmaceutical interventions implemented by individuals, communities, and governments to reduce the risk and scale of infection during a health emergency such as an epidemic or pandemic [1]. PHSMs play a role through all phases of such emergencies, especially while clinical countermeasures (vaccines and therapeutics) are being developed and deployed. It is therefore important to understand the effectiveness of PHSMs and their potential unintended consequences to allow governments and public health organizations to prepare for epidemics and pandemics.

Contact tracing (CT) is one important PHSM, defined by the World Health Organization (WHO) as the process of identifying, assessing, and managing people who have been exposed to someone who has been infected with the infection of interest [1]. CT may take many forms or be a component of multicomponent interventions and is an important element of organizational responses to any infectious disease. This review focused on CT, as well as innovations linked to CT that are generalizable across a range of infectious diseases.

One systematic review of CT in the control of infectious diseases from 2022 found that provider-initiated CT can be effective in managing infectious diseases but that evidence was relatively scarce outside of tuberculosis (TB), highly heterogeneous, and of mixed quality [2]. Similar reviews of CT have been published in relation to COVID-19 [3-8]. These reviews either included modeling or simulation studies, used broader definitions of CT that included human resource–intensive strategies such as active case finding, CT in combination with other measures, or included aspects of CT that are not generalizable to pathogens that spread by other routes of transmission (such as partner-delivered treatment for sexually transmitted infections [STIs]). Given the variability in the existing review evidence, and our research aim to have a comprehensive review of CT in the context of epidemic or pandemic response, a de novo systematic review examining the effectiveness of CT for different pathogens and routes of transmission is warranted.

### Research Question

What is the effectiveness of CT in reducing transmission of infectious disease as part of an epidemic or pandemic response?

## Methods

### Overview

The protocol for this review was drafted in conjunction with key interest holders at the UK Health Security Agency and a patient and public involvement and engagement group (described below). It was prospectively registered on PROSPERO (International Prospective Register of Systematic Reviews) prior to commencement of the review (CRD42025628278). The review was reported following the PRISMA (Preferred Reporting Items for Systematic Reviews and Meta-Analyses) guidelines [9] (see checklist in Multimedia Appendix 1). This was a rapid review conducted between November 2024 and April 2025. Full methodological details are available in the protocol and full report [10].

### Identification of Studies

The search strategy was developed in MEDLINE by an information specialist (JB), peer reviewed by a second information specialist (AB), and translated for the other databases. Validated search filters for randomized controlled trials (RCTs) were obtained via the InterTASC Information Specialists’ Sub-Group (ISSG) Filters Resource [11] for searching MEDLINE [12], Embase [12], and CINAHL Ultimate [13] and nonvalidated search filters for observational studies [14], evaluation studies, and nonrandomized trials or quasi-experimental studies [15].

The list of diseases was developed and validated with subject matter experts as a pragmatic list of representative diseases of each of the 5 routes of transmission (respiratory, vector-borne, direct contact, blood-borne, food-borne, and water-borne [ingestion]). The searches used a combination of relevant controlled vocabulary terms (eg, MeSH [Medical Subject]) and free-text terms.

A total of 6 bibliographic databases were searched between November 29 and December 3, 2024: MEDLINE (1946-current), Embase (1974-current), and Global Health (1973-current) via Ovid; CINAHL Ultimate (1937-current) via EBSCOhost; Cochrane Central via Cochrane; and Scopus (1788-current) via Elsevier. No date or language restrictions were used. Results were downloaded into EndNote (version 20; Clarivate Analytics), which was used for finding and removing duplicates.

Supplementary searching involved forward and backward citation searching of included references using Scopus. After deduplication using EndNote, the remaining 1996 records were single screened. Search strategies are available in Multimedia Appendix 2.

### Inclusion and Exclusion Criteria

The inclusion and exclusion criteria were defined in consultation with interest holders and are summarized below. A more detailed description is provided in Multimedia Appendix 3.

#### Participants or Population

Include: all humans in the context of an outbreak, epidemic, or pandemic of one of the prespecified infectious diseases of interest (full list provided in Multimedia Appendix 3), using WHO definitions for an outbreak, epidemic, or pandemic [16]. We focused on conditions with the potential to reach epidemic or pandemic status, as it was considered that this could help inform the use of CT to prevent an outbreak from escalating.

#### Intervention

The inclusion criteria were as follows: interventions that incorporate CT, defined as the process of starting from a confirmed case diagnosed with an infectious disease (index case), identifying who the index case may have come into contact with, and attempting to communicate with the contacts. CT may be a stand-alone intervention or part of a multicomponent intervention that may involve further management of contacts (eg, treatment or quarantine) or other activities to improve infection detection (eg, education and training of health care staff). Interventions should take place in a community setting (including CT that is led by a health care setting but not limited to that health care setting).

Exclusion criteria were as follows: any other form of PHSMs or intervention to reduce transmission that does not incorporate CT; studies in health care settings where the goal was control of nosocomial outbreaks.

#### Comparator

Different CT approaches compared with each other or compared with no CT were included.

Other PHSMs as comparators where these were not part of background measures in both arms were excluded.

#### Outcomes

Studies with at least 1 measure of effectiveness (ie, measures of transmission of the focal disease, health care use, or mortality) or unintended consequences were included, as detailed in Multimedia Appendix 3.

#### Study Design

Experimental studies with a control group, including RCTs, quasi-experimental studies, and before-and-after studies, and observational studies with a control group, including cross-sectional and case-control cohorts were included.

Studies without control groups, reviews of any type, crossover study designs, modeling studies, case series, case reports, and qualitative studies were excluded.

We included only peer-reviewed research published in English, with no restriction on date of publication or setting.

### Selection of Evidence

After piloting, the title and abstract of each record retrieved by the search were screened by 2 independent reviewers (MN, SF, MW, K Becker, RA, JTC, GJMT, and JB). The full text of each remaining record was then screened by 2 independent reviewers (MN, SF, MW, and K Becker). Disagreements at each stage were resolved through discussion. Articles excluded at full text were coded to indicate the first reason for exclusion. Several additional clarifications to the inclusion criteria were needed as study selection progressed due to the complex nature of interventions:

- Testing interventions were included if testing (ie, partner-delivered testing kits) was part of the CT process.
- Treatment interventions were excluded if there was no difference in CT activity between arms.
- Community-led surveillance interventions (where “community hot spots” with high disease prevalence were actively targeted for screening) were excluded.
- Any paper in which only proxy measures for a relevant outcome were presented was excluded.
- Active case finding or intensified active case finding was included if the process began from a known index case, rather than indiscriminate case finding based on location, for example.

### Prioritization

Because of the volume of evidence identified, we prioritized the most robust evidence (studies with concurrent control). This was a protocol deviation and was registered on PROSPERO. Top-level data were extracted from nonprioritized studies, tabulated, and briefly narratively described.

### Critical Appraisal

After piloting, each included trial was critically appraised using a modified version of the Effective Public Health Practice Project Quality Assessment Tool for Quantitative Studies, a validated tool suitable for multiple study designs [17]. Each study was rated either “strong,” “moderate,” or “weak” in 6 domains, with the number of “weak” scores given per study used to determine its overall score: studies were rated as “strong” if they had no “weak” scores, “moderate” with 1 “weak” score, and “weak” if they had 2 or more “weak” scores. Modifications and interpretations established during critical appraisal are described in the full report [10].

Appraisal was independently conducted by 2 reviewers (K Becker, MW, SF, LS, and RW), with disagreements resolved through discussion. The findings of critical appraisal informed the interpretation of results and did not influence the inclusion of studies in the review or the synthesis.

### Data Extraction

After piloting, key information was extracted from included trials by 1 reviewer and checked by a second (K Becker, MW, SF, and RA). Discrepancies were resolved through discussion. Data were extracted in relation to author and study details, sample characteristics, trial arm details, outcomes, outcome data, and inequalities addressed.

### Synthesis

Extracted data were tabulated and narratively described. Included studies were grouped based on route of transmission and condition or infection. STIs were grouped within the blood-borne route to align with the approach of the Department of Health and Social Care pandemic preparedness program at the time of conducting the review [18]. Interventions and outcomes were then described within those groups, with studies presenting similar interventions and comparators discussed together where possible. Comparators were described as usual care or standard of care as described by study authors. The CT element of each intervention was categorized into 1 of 4 groups:

- Provider-initiated contact tracing (PICT): CT is instigated by a trained provider (often health care worker) who notifies the index case’s contacts.
- Partner referral (PR): CT is instigated by the index case who notifies their contacts.
- Household investigation (HI): the home of the index case is visited by a health care worker to notify and assess household members for signs of infection.
- Technology-based contact tracing (TECH): CT facilitated using digital tools, programs, or applications.

Contract referral (where the index case is given a set amount of time to contact their partners before a health care worker notifies the contacts) was categorized as a mix of both PICT and PR.

Where study design, route of transmission, intervention, comparator, and outcome were similar in multiple studies, we considered meta-analysis. In the event, heterogeneity precluded this, and we opted for a narrative synthesis approach, guided by Synthesis Without Meta-Analysis [19].

### Patient and Public Involvement and Engagement

This review benefited from discussions with PERSPEX (Public Engagement in Research for Health and Social Policy at Exeter), a group of 17 public collaborators who bring their carer, patient, or public perspective to the work of Isca Evidence. PERSPEX members meet monthly online, and membership is culturally, geographically, and demographically diverse. The review team discussed the project 3 times with PERSPEX, enabling the review team to gain insights about CT from a patient, carer, and public perspective.

## Results

### Study Selection

**Figure 1:**
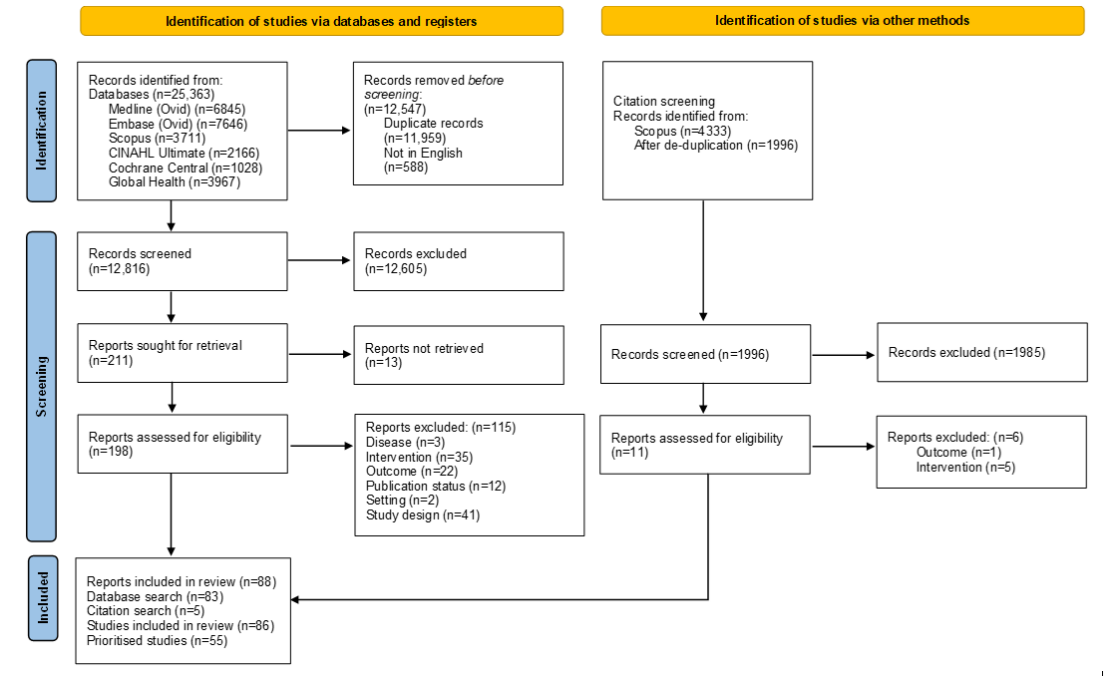
PRISMA (Preferred Reporting Items for Systematic Reviews and Meta-Analyses) flowchart summarizing the results of the literature search and screening for eligibility.

The initial searches identified 25,363 records. After deduplication and removal of non–English-language references, a total of 12,816 titles and abstracts were screened for relevance. After excluding 12,605 records for clearly not meeting 1 or more inclusion criteria, a total of 211 full-text articles were sought. Thirteen were unavailable, resulting in 198 records assessed for eligibility at full text. A total of 5 additional studies were found through supplementary searching. The most common reasons for exclusion at the full-text stage were intervention (40 reports) and study design (41 reports). Finally, 88 reports (of 86 studies) were included in the review. Of these, 57 reports (of 55 studies) were prioritized. The PRISMA flow diagram shown in Figure 1 summarizes this process. Multimedia Appendix 4 contains a list of studies with reasons for exclusion at full-text. Multimedia Appendix 5 contains the search summary table, which documents the origin of included references [20].

### Sample Characteristics

Table S1 in Multimedia Appendix 6 displays the sample characteristics of the 55 prioritized studies, reported in 57 papers [21-77], including study design and location, condition of interest, study dates and sample sizes, settings and recruitment criteria, and sample demographic characteristics. A total of 36 studies were a form of RCT [21,22,24-31,35-39,42,43, 45,51-60,65-67,69-71,74-77]. The 19 non-RCT studies included 9 observational studies with concurrent control [23,32,33,44,61-64,73], 4 controlled before-and-after studies [46-49], 3 interrupted time series [40,41,50], and 3 nonrandomized trials with concurrent controls [34,68,72]. Populations across 6 continents were sampled, with the majority taking place in Africa (n=18 [24,26,27,29,32,39,42,43,47,48, 50,51,54,60,65,66,70,76]), North America (n=13 [44,52,53,56-58,61,63,68,71,72,74,77], and Europe (n=11 [21,22,34-38,40,41,59,64,69,73]. A total of 8 studies took place in Asia [23,28,45,46,49,55,62,67], 3 in South America [25,30,75], 1 in Oceania [33], and 1 across Asia and Oceania [31].

All evidence was related to either respiratory or sexual or blood-borne routes of transmission. Within the respiratory route, 5 studies related to COVID-19 [40,41,64,73,76] and 15 to TB [23,25,42,43,46-51,54,55,65,67,75]. Under the sexual or blood-borne route, 35 studies were related to STIs [21,22,24,26-39,44,45,52,53,56-63,66,68-72,74,77]. Of the STIs, 13 studies included HIV [24,26-29,31,32,44,45,58, 60,62,63], 13 chlamydia [21,22,33-38,52,53,56,59,68,69,77], 7 gonorrhea [34,39,53,56,68,72,77], 3 nongonococcal urethritis or urethritis [34,52,56], 6 syphilis [30,39,44,61,70,71], and 3 trichomoniasis [39,57,74]. Several papers investigated more than 1 STI [34,39,44,52,53,56,68,77]. One paper included chancroid and lymphogranuloma venereum alongside syphilis, gonorrhea, and trichomoniasis [39], while another referred to STIs but did not specify which ones [66].

Where reported, sample size ranged from 55 [31] to 59,027 [41], with the total sample in this review being 125,635 participants. A total of 27 studies were set in a form of clinic or outpatient services [22,24,26-28,32-39,42,45,50-52,54,56,58, 62,63,66,68,70,72,74,78]. Of these, 14 studies were in generalist services [32,33,35,36,39,42,45,50,51,54,58,68,70,72,74] and 13 in services specialized for the condition of interest, such as an STI clinic [22,24,26-28,34,37,38,52,56,62,63,66,77]. A total of 19 studies took place in the community [21,23,25,29,41,47-49,55,59,61,64,65,67,69,71,73,75,76] and 5 in both clinic and community settings [30,43,44,57,60]. One study took place in both a clinic and on the internet [53], 1 was set in a prison [31], 1 in a nationwide program [40], and 1 setting was not reported [46].

### Critical Appraisal

Of the 55 appraised studies [21-77], a total of 9 received an overall rating of “strong” [28,42,44,57,64,65,70,76,77], 18 received an overall rating of “moderate” [21,26,30,39,40,45,49,51,52,55,59-61,67,69,71,74,75], and the remaining 28 were rated as “weak” [22-25,27,29,31-38,41,43,46-48,50,53,54,56,58,62,63,66,68,72,73]. Overall, the criteria on which studies scored most highly were study design and data collection. Studies scored most poorly on criteria related to researcher and participant blinding. Ratings are summarized in Table 1. A more detailed description of critical appraisal findings is available in the full report [10].

**Table 1:** Section and overall critical appraisal ratings for prioritized studies.

Study ID	Selection bias	Study design	Confounders	Blinding	Data collection	Withdrawals	Overall rating
Andersen et al [21]	MO^a^	ST^b^	WK^c^	MO	ST	ST	MO
Apoola and Beardsley [22]	ST	ST	WK	WK	ST	ST	WK
Bai et al [23]	MO	WK	WK	WK	ST	N/A^d^	WK
Brown et al [24]	MO	ST	ST	WK	ST	WK	WK
Cavalcante et al [25]	WK	ST	ST	WK	ST	WK	WK
Chen et al [26]	MO	ST	ST	WK	ST	ST	MO
Cherutich et al [27]	MO	ST	WK	WK	WK	ST	WK
Chiou et al [28]	MO	ST	ST	MO	ST	ST	ST
Choko et al [29]	ST	ST	WK	WK	ST	MO	WK
Clark et al [30]	MO	ST	ST	MO	WK	ST	MO
Culbert et al [31]	ST	ST	WK	WK	WK	ST	WK
Dibia et al [32]	MO	WK	WK	MO	ST	N/A	WK
England et al [33]	MO	MO	WK	WK	ST	N/A	WK
Estcourt et al [34]	WK	WK	WK	WK	WK	WK	WK
Estcourt et al [35,36]	MO	ST	ST	WK	WK	MO	WK
Estcourt et al [37,38]	WK	ST	ST	WK	ST	WK	WK
Faxelid et al [39]	MO	ST	ST	MO	WK	N/A	MO
Fetzer and Graeber [40]	MO	MO	WK	MO	ST	N/A	MO
Findlater et al [41]	MO	WK	WK	MO	ST	N/A	WK
Hanrahan et al [42]	MO	ST	ST	MO	ST	ST	ST
Hanrahan et al [43]	MO	ST	ST	WK	ST	WK	WK
Heumann et al [44]	MO	MO	ST	MO	ST	N/A	ST
Hu et al [45]	MO	ST	ST	MO	ST	WK	MO
Huang et al [46]	MO	MO	WK	WK	ST	WK	WK
Jerene et al [47]	MO	WK	WK	WK	ST	WK	WK
José et al [48]	MO	MO	WK	WK	ST	N/A	WK
Joshi et al [49]	MO	MO	WK	MO	ST	N/A	MO
Kagujje et al [50]	MO	MO	WK	MO	ST	WK	WK
Kaswaswa et al [51]	WK	ST	ST	MO	ST	ST	MO
Katz et al [52]	MO	ST	ST	MO	WK	N/A	MO
Kerani et al [53]	WK	ST	WK	MO	WK	MO	WK
Ketema et al [54]	MO	ST	WK	MO	ST	WK	WK
Khatana et al [55]	MO	ST	ST	MO	ST	WK	MO
Kissinger et al [56]	MO	ST	ST	WK	WK	MO	WK
Kissinger et al [57]	MO	ST	ST	MO	ST	ST	ST
Landis et al [58]	WK	ST	WK	WK	WK	N/A	WK
Low et al [59]	MO	ST	WK	MO	MO	MO	MO
Lugada et al [60]	MO	ST	ST	MO	ST	WK	MO
Lukac et al [61]	ST	MO	WK	MO	ST	MO	MO
Luo et al [62]	WK	WK	WK	WK	ST	N/A	WK
Malave et al [63]	ST	MO	WK	MO	WK	N/A	WK
Malheiro et al [64]	MO	MO	ST	MO	ST	N/A	ST
Martinson et al [65]	MO	ST	ST	MO	ST	ST	ST
Mathews et al [66]	WK	ST	WK	MO	MO	ST	WK
Morishita et al [67]	ST	ST	WK	ST	ST	N/A	MO
Oh et al [68]	MO	MO	WK	MO	WK	N/A	WK
Østergaard et al [69]	WK	ST	ST	MO	ST	MO	MO
Parkes-Ratanshi et al [70]	MO	ST	ST	MO	ST	ST	ST
Peterman et al [71]	ST	ST	ST	WK	ST	ST	MO
Potterat and Rothenberg [72]	MO	ST	WK	WK	WK	N/A	WK
Raymenants et al [73]	MO	MO	WK	MO	WK	ST	WK
Schwebke and Desmond [74]	MO	ST	ST	MO	ST	WK	MO
Shah et al [75]	MO	ST	ST	MO	ST	WK	MO
Tchakounte et al [76]	MO	ST	ST	MO	ST	MO	ST
Wilson et al [77]	MO	ST	ST	MO	ST	ST	ST

^a^MO: moderate.

^b^ST: strong.

^c^WK: weak.

^d^N/A: not applicable.

### Deprioritized Studies

Table S2 in Multimedia Appendix 6 displays the 31 studies that were deprioritized because of lack of a concurrent control. Like the prioritized evidence, the deprioritized evidence consisted largely of STI studies, with 9 studies on bacterial infections (eg, chlamydia) [78-86] and 8 studies on HIV [87-94]. In addition, there were 11 studies focused on TB [95-105], 2 on COVID-19 [106,107], and 1 on chronic hepatitis B [108]. None of the 31 deprioritized studies investigated a route of transmission that was not already covered by the prioritized evidence. Sample sizes ranged from 70 [78] to 2,022,127 participants [101], and the median was 919 (IQR 253-3192). Most studies compared an intervention with some variation of standard practice protocol (n=24).

### Synthesis

#### Respiratory or Airborne Transmission

Studies of CT in respiratory transmission focused on TB and COVID-19. Table S1 in Multimedia Appendix 7 summarizes the characteristics, key components, and overview of outcomes for these studies. Further details about the characteristics of interventions evaluated within these studies are available in Table S2 in Multimedia Appendix 7, and detailed outcome data are available in Table S3 in Multimedia Appendix 7. More extensive interpretation is available in the full report [10].

#### Characteristics of CT Interventions in TB

Of the 15 trials focusing on TB, 6 were cluster RCTs [25,42,51,65,67,75], 3 RCTs [43,54,55], 4 controlled before-and-after designs [46-49], 1 interrupted time series [50], and 1 observational study [23]. The TB studies were conducted outside high-income countries, except for 1 study conducted in Taiwan [46]. Nearly half of the TB studies were rated as being of weak quality, with only 2 rated as strong [42,65].

The interventions in 7 of the 15 TB studies incorporated PICT methods [27,29,47,50,53,54,58], 4 were HI [51,55,65,79], 3 involved a combination of PICT and HI [46,52,71], and 1 involved PR [55]. The majority of TB interventions were community-based, and most aimed to counteract the disease by encouraging people to attend health care facilities for screening or by facilitating access to home-based screening; 11 of the 15 interventions involved home visits [23,42,47-50,55,65,67,75]. Home visits varied in nature, but tended to involve health care staff [23,55] or recruited community members [46-48,50,67] carrying out symptom screening at the home. Some studies also carried out testing in the visits [55,65]. A total of 3 studies involved community members [46,47] or research fieldworkers [65] following up patients’ households for monitoring, while incentives were offered to contacts to present for screening in 4 studies [23,25,42,43]. Several studies included training components in which health care workers or key community figures were educated to help carry out the intervention (n=8) [23,46-48,50,54,55,67].

A total of 6 studies specifically focused on improving case finding in younger ages [51,52,54,55,59,75]. Three studies referred contacts aged 5 years or younger irrespective of symptom status [51,52,59]. Three focused their intervention on children aged 15 years or younger [75]. Some interventions were multicomponent, with activities such as attempts to increase awareness of TB [50] and screening children at school [49] being examples of additional components.

TB interventions were usually compared with a form of standard care. Often this was a national program, and in all but 1 comparator this involved passive case finding, whereby potential cases self-report for testing; the 2023 study by Hanrahan et al [43] used household CT. In 4 studies, the control or baseline program was described as a form of directly observed treatment, short-course (DOTS) program that was adapted or enhanced to improve CT and case finding [23,25,46,49]. It is likely that baseline programs used in other studies resembled DOTS, but they instead described their comparators as a national standard of care [25,42,47,50,55]. One study incorporated CT and active monitoring vs CT and passive monitoring [75].

TB interventions tended to be longer or have longer follow-ups than COVID-19 and STI interventions. The median and most frequently reported intervention duration (or time until last follow-up) was 12 months (n=5) [23,47-49,54], with a range of 3 months [51] to 5 years [25].

While all TB studies involved treatment for contacts with a pharmaceutical in both the intervention and comparator groups, some interventions focused more heavily on improving this aspect compared with the comparator. In particular, 7 studies used various approaches to encourage treatment access or adherence [25,46-48,50,54,65]. These approaches included integration of indigenous case managers, community-led home visits, improving access to chest radiography for young children, and provision of prophylaxis.

#### Synthesis of Findings of CT in TB

##### Disease Incidence or Prevalence

Six of 7 studies reporting TB incidence or prevalence observed a decrease in this outcome with CT [23,25,48,50,67,75]. Effect estimates varied in nature; no between-group statistical analyses were performed in 2 studies [50,67], and 4 of these studies were rated as weak quality [23,25,48,50]. Therefore, findings should be interpreted with caution.

All 6 interventions associated with reduced disease incidence or prevalence used home visits, and 3 included active follow-up of contacts [23,25,48]. The 1 study showing no effect also included home visits, but the authors speculated that working in a high-burden area, where motivation for testing and treatment was already high, may explain their null findings [65].

##### Case Detection

A total of 4 studies reported case detection outcomes, with 2 studies showing greater numbers of cases detected with intervention [49,55] and 2 showing no difference [51,54]. In the controlled before-and-after study by Joshi et al [49] intensified childhood TB case finding yielded 33% more cases after the intervention compared with 16% more in regions without intensified childhood TB case finding. Intensified case finding included activities such as household contact screening and mobile chest health camps, while usual care was poorly described. However, standard DOTS took place separately for TB cases as usual in both intervention and control areas, as per national guidelines [49]. In the quasi-RCT by Khatana et al [55], their active case-finding strategy led to a nearly 4-fold increase in the odds of detecting TB cases (odds ratio [OR] 3.97, 95% CI 1.73-9.11).

##### Treatment Rates

A total of 6 TB studies reported treatment rates. The 3 studies reporting increased treatment rates were all of weak quality [46,47,54]. Only 1 of these outcomes related to successful completion of treatment [46], while all others recorded initiation of treatment. Two of the 3 studies reporting increased treatment rates involved interventions designed to be culturally relevant or sensitive to the populations of interest, with usual care being generic [46,47]. In both cases, this meant that intervention deliverers understood local customs and dialects and could access people living in remote areas [46,47].

Only 3 studies reported outcomes in more than 1 category [51,54,65], with mixed findings in all instances. No studies reported on the unintended consequences of CT.

#### Characteristics of CT Interventions in COVID-19

A total of 5 trials evaluating CT in COVID-19 were included [40,41,64,73,76], based in the United Kingdom, Belgium, Portugal, and Cameroon. Two studies were rated as strong quality [64,76], 1 as moderate quality [40], and 2 as weak quality [41,73]. Interventions in 3 studies were categorized as technology-based CT methods [44,45,80], and 2 incorporated PICT [68,77].

The cluster RCT by Tchakounte et al [76] investigated digitized CT using a smartphone app. This intervention involved individuals registering for an app, where upon receiving a positive test, their information was sent to the CT unit, a clinical interview conducted, and automated text messages sent to notify contacts of their exposure to COVID-19. A total of 2 studies reported on the same natural experiment which occurred due to an error in the UK CT system [40,41], when a technical issue caused around 16,000 positive cases to be missed by the national CT system, thereby delaying the tracing and self-isolation of missed cases. Finally, there were 2 cohort studies with concurrent control groups [64,73]. One study evaluated a standard COVID-19 CT program with contact-eliciting interviews and mandatory quarantine [64]. Symptomatic close contacts were then referred for evaluation and testing [64]. The other program tested different durations of tracing windows, with contacts traced if their last interaction with the index case was 2 days before onset or test for the standard window, and 3-7 days in the extended window group [73]. Comparators were standard care protocols including national CT programs [40,41], as well as manual CT [76] and no CT [64,73]. The median intervention period was 1 week, and 3 studies had a 1-week intervention arm [40,41,73]. Intervention durations ranged from 2 days [73] to 23 weeks [76]. Interventions were delivered by local public health authorities [64], government contact tracers [73], and health care workers [76].

#### Synthesis of Findings of CT in COVID-19

Both studies evaluating the impact of CT on disease prevalence found positive associations [40,73]. Fetzer et al [40], reported there was an association between delayed notifications to quarantine, as a result of a fault in the UK Test & Trace program, and increased disease prevalence and mortality per case traced with a 6-14 day delay (multiple outcomes in Table S3 in Multimedia Appendix 7) [40].

Extending the backward CT window from 2 to 3-7 days led to 42% more cases being detected among contacts [40,73]. However, 3 studies found no impact of CT on case detection rates [41,64,76].

Additional outcomes were reported in the 2 studies evaluating the effect of delayed notification of close contacts due to an error with the UK Test & Trace system [40,41]. In the study by Findlater et al [41], they observed that the delay in CT led to primary contacts of index cases being slightly more likely to be admitted to hospital within 14 days (OR 1.1, 95% CI 1.0-1.2), although there was no impact on patient mortality or hospital admission rates in their secondary contacts. Fetzer et al [40] reported higher mortality rates in the delayed CT group (additional 0.007 deaths per capita; SE 0.003; *P*<.05). No studies reported on the unintended consequences of CT.

#### Summary

A total of 15 evaluations focused on TB [23,25,42,43,46-51,54,55,65,67,75], and 5 on COVID-19 [40,41,64,73,76]. Studies on TB typically evaluated interventions with the broad aim of encouraging the identification, testing, and treatment of contacts via home visits, active follow-up, incentives, and other measures. There was just 1 study that incorporated CT and active monitoring vs CT and passive monitoring [75]. Findings were inconclusive across outcome domains and studies, but the greatest change associated with intervention was observed for reductions in disease incidence or prevalence. However, the nature of the evidence, which was highly heterogeneous in design and quality, means that only tentative conclusions may be drawn. Five studies of varying designs, quality and outcomes, provide limited evidence about the impact of CT interventions on outcomes in the context of COVID-19.

### Sexual or Blood-Borne Transmission

#### Overview

A total of 35 studies focused on CT in sexually transmitted or blood-borne diseases. Table S1 in Multimedia Appendix 8 provides a broad overview of their key characteristics, trial arm components, and findings. Further detail about the characteristics of interventions evaluated within these studies is available in Table S2 in Multimedia Appendix 8, and detailed outcome data are available in Table S3 in Multimedia Appendix 8.

#### Characteristics of CT Interventions in STIs

Of the 35 prioritized studies falling into the sexually transmitted route of spread, 13 included chlamydia [21,22,33-38,52,53,56,59,68,69,77], 13 included HIV [24,26-29,31,32,44,45,58,60,62,63], 6 included syphilis [30,39,44,61,70,71], 6 included gonorrhea [34,53,56,68,72,77], 3 included nongonococcal urethritis [35,52,56], 3 included trichomoniasis [39,57,74], 1 included chancroid and lymphogranuloma venereum [39], and 1 did not specify which STIs were included [66]. A total of 7 trials evaluated 2 intervention arms [24,29,34-36,52,70,71], and 2 evaluated 3 intervention arms [30,62].

The duration of the interventions was recorded in 28 studies [22,24,26-31,33-39,45,53,56-58,62,63,66,68-72,74,77]. The duration ranged from as little as 45 minutes for the counseling session in the study by Mathews et al [66] to 13 months, the whole study period that personnel were introduced in the study by England et al [33], but 82% of the interventions were less than 6 months in duration. Most interventions involved a clinic setting (n=26) [22,24,26-30,33,34,37-39,44, 45,52,53,56,57,62,63, 66,68,70-72,74,77] or took place within the community (n=6) [21,57,60,61,69,71]. One study was based in a prison with community follow-up of partners [31]. Two were primary care–based [35,36,59], 1 an unspecified health facility [32], and 1 a public health facility [58]. Interventions were delivered by a range of different health care professionals, and in all but 11 studies [24,26,34,52,57,59,60,63,68,74,77] this was the same for both intervention and control.

As the interventions were broadly similar across all conditions within this group, they have been summarized together.

One study involved household contact investigation where all household members of the index case were identified and tested, not solely sexual partners of the index case [60]. In all remaining studies, the CT method involved partner notification (PN), where the sexual partners of patients diagnosed with an STI were identified and informed of their exposure to an infection and the need to be tested and treated [21,22,24,26-39,44,45,52,53,56-59,61-63,66,68-72,74,77]. One study also included notification of social contacts [26]. The majority of the PN methods were patient referral notification, where the index patient was responsible for informing their partners of their risk of exposure and referring them to services for testing (n=17) [21,22,29,30,34-39,53, 56,57,59,61,69,70,72,77]. Contact or referral slips were used in 11 of these studies [22,24,26,30,39,56,57,59,70,72,77], and 2 studies trialed the use of a web-based notification service [30,53]. Provider referral (where notification was instigated by a health care professional) was used in 9 studies [24,27,32,44,52,62,63,68,71], and 3 studies involved contract referral, whereby the index case was given limited time to notify partners before the provider stepped in [24,26,74]. In 5 studies, index cases had the choice of PN approach [28,31,33,45,58,66]. In 1 study, provider referral was used if the index case refused to notify their partners [39], and in another the index case could choose which method of PN to use (patient-initiated with nurse support or provider-initiated) [61].

In 4 studies, the nature of the partner elicitation interview with the index patient was varied [44,59,72,74]. A total of 12 studies incorporated reminders or active follow-up as part of their interventions [27,29-31,35-38,45,52,62,68,70,72].

A total of 12 studies adapted the testing process to encourage partners to engage with testing, thereby enhancing CT [21,22,27,29,31,34-38,45,62,69,71]. In 7 of these studies, index cases were provided with sampling kits to pass directly to partners [21,22,29,37,38,45,62,69]. In 2 studies urine sampling was evaluated rather than swab sampling [21,22]. Of the 7 home-based testing interventions, 3 studies provided the means to return samples to the laboratory [21,45,69], whereas partners or index cases were responsible for returning tests to a site in 3 studies [22,29,62]. The study by Choko et al [19] had an additional intervention arm incorporating a financial incentive for positive partners that attended clinic [29]. Estcourt et al [34-38] conducted 3 studies that involved the provision of sampling kits and treatment either after partner consultation through a telephone hotline [35-38] or consultation with a pharmacist [34-36]. Sampling kits and treatment were provided to the index case for their partners in the 2022/2024 study [37,38], and this was also an option for some partners in the 2015/2016 study, while other partners collected the tests and treatment from clinic reception or from the pharmacist [34-36]. Luo et al [62] incorporated an additional intervention arm where testing was completed as a couple in a clinic setting [62]. Home or field testing was offered to partners in 4 studies [27,31,60,71].

Anonymity of the index case was a feature of the interventions in 8 studies [26,30-32,45,62,67,68], 5 of these were HIV studies [26,31,32,45,62].

New or specialist personnel were introduced to deliver PN services in 5 studies [33,52,59,60,63,74]. In 1 study this was the introduction of 2 public health officers to assist with CT [33], and in another a practice nurse–led PN strategy was evaluated [59]. In 3 studies disease intervention specialists were used [52,63,74]. The introduction of different staff allowed for some studies (n=3) to evaluate the effectiveness of delivering a field-based PN service [52,60,74].

A total of 5 studies incorporated additional counseling and education for the index case, in part providing extra time and support to plan for PN [28,33,39,66,77]. Index cases in the study by Chiou et al [28] also had phone access to a counselor during working hours and through an app or via email 24 hours/day [28].

Most of the study comparators (n=25) involved patient referral PN methods [21,22,24,26,27,29-31,34-38,45,52, 53,56-58,60,62,68-70,74,77], and of these, 13 used contact or referral slips, cards, or vouchers as a means of facilitating notification [20,21,23,25,28,29,33,51,57,59,67,69,76]. A total of 3 studies involved provider referral comparators [44,61,63], 2 involved contract referral [71,72], 4 included various PN methods [28,32,33,59]. In 1 study, no detail was given for which method of PN the comparator used [66]. Two comparators involved no CT [27,39]. In the study by Cherutich et al [27], the comparator arm was delayed (by 6 weeks) provider-initiated PN (compared with immediate provider PN in the intervention arm), and outcomes were collected prior to this delayed PN occurring. The study by Faxelid et al [39] did not involve PN.

In 30 studies, the comparator was the usual care or standard of care [21,22,24,26,28-39,44,45,52,53,56-60,66,68-70,72,74,77]. In 11 studies, the intervention group received usual care or standard care plus an additional element in the intervention [28,30,34-38,45,53,66,70,74].

Information (written or online) provided to the index case was a feature of the comparator in 6 studies [30,34-38,68,74]. In 9 studies, the comparator involved a counseling session or interview with the index case, which was face-to-face in 8 studies [24,28,31,52,58,66,72,77] and telephone based in 1 study [44]. The comparator group in 9 studies involved some form of follow-up [28,30,31,37,38,45,58,62,68,70]. This predominantly involved follow-up of the index case (n=7) for feedback and experience on partner notification [28,31,37,38,45,62,68,70]. The comparator groups in 2 studies followed up partners directly [30,58].

#### Synthesis of Findings of CT Interventions in STIs

##### Case Detection

Case detection was the most frequently assessed outcome domain, being evaluated in 21 studies [21,24,26-29,31-33,44,45,58-63,69-72]. Seven of these studies observed that interventions were associated with a favorable outcome [26,27,29,31,44,60,69], while the rest observed no difference between intervention arms. In addition, the study by Luo et al [62] provided case detection rates but did not analyze between-group differences. All but 1 of the positive outcomes came from studies of HIV, including in the study by Heumann et al [44], which focused on both HIV and syphilis and only found improved detection of the former. The study by Lugada et al [60] may be considered an outlier in that it used household contact investigation to test for HIV rather than relying on PN. Compared with clinic-based detection, this led to a near 3-fold increase in the odds of case detection (OR 2.76, 95% CI 1.97-3.86; *P*<.001). The study by Ostergaard et al [69] found that home sampling, rather than office sampling, was associated with more infected partners identified per index case (mean difference 1.63 cases; 95% CI 1.1-2.3).

The remaining 5 studies observing positive case detection outcomes used a wide variety of intervention components, and other than the use of active follow-up in 3 studies [26,27,31], they rarely shared common components. Comparators were more passive forms of CT, including passive “self-tell” PN [26,31], telephone rather than in-person contact elicitation interviews [44], delayed notification [27], and referral slips alone [29]. Given that the components used in the successful studies were also used across several other interventions showing no effect, it is unclear whether specific components are associated with success. For example, the studies led by Brown et al [24], Hu et al [45], and Chen et al [26] all used a form of provider-led PN in their interventions, all in HIV samples, but while Chen et al [26] saw an improvement in case detection rates, Brown et al [24] and Hu et al [45] observed no differences.

The magnitude of effect varied widely, with Chen et al [26] finding the ratio of persons referred per index case (ratio 1.9, 95% CI 1.2-3.1) and new cases per index case (ratio 2.0, 95% CI 1.2-3.2) to be nearly doubled with intervention, and Cherutich et al [27] finding a nearly 5-fold greater likelihood of detecting new cases per index (incidence rate ratio [IRR] 5.0, 95% CI 3.2-7.9). Choko et al [29] detected 4.3% more new HIV-positive contacts with HIV self-testing kits and 6.9% more with the addition of an incentive to HIV self-testing [29], while Culbert et al [31] identified 5 new HIV-positive partners compared with 0 with assisted PN vs self-tell alone (no statistics provided) [31]. Finally, Heumann et al [44] found that the number of partners diagnosed with HIV was more than twice as high in the in-person interview arm compared with the telephone interview arm (risk ratio [RR] 2.17, 95% CI 1.04-4.50) [44].

##### Treatment Rates

A total of 15 studies looked at treatment rates, with 7 finding that interventions were associated with improvements [27,34,39,44,52,56,61]. Two of these studies included people living with HIV [27,44]; the rest were interested in multiple STIs [34,39,52,56,61]. Similarities between successful studies were minimal, with interventions having different approaches and components, all of which were also present in unsuccessful studies. In the study by Cherutich et al [27], treatment rates were more than 4 times higher at 6 weeks in the group that was immediately assisted with PN services compared with the group that received no services (IRR 4.4, 95% CI 2.6-7.4) [27]. The addition of a consultation hotline (24% greater) or consultation with a pharmacist (23% greater) led to greater treatment rates than routine PN in the study by Estcourt et al [34]. In-person interviews, rather than telephone interviews, led to a 19% greater likelihood of partners being treated for syphilis in the study by Heumann et al [44] (RR 1.19, 95% CI 1.03-1.37). Katz et al [52], in their study from 1988, found that active follow-up was associated with increased treatment rates among contacts compared with both an interview (72% vs 22% treatment rates; *P*<.001) and standard nursing referral (72% vs 18%; *P*<.001), while Kissinger et al [56] found that the provision of booklets led to greater odds of contacts of patients with STIs being treated (OR 1.66, 95% CI 1.22-2.27). Faxelid et al [39] observed that individual counseling and assisted PN resulted in a greater proportion of partners of male index patients attending treatments than with standard care (no CT; 85% vs 55%); however, this was not observed in partners of female index patients (65% vs 56%). In the study by Lukac et al [61], patient-initiated PN, with the support of public health nurses, led to better treatment rates than provider-initiated PN (65% vs 59%, no statistics provided).

##### Disease Incidence or Prevalence

A total of 7 studies evaluated disease incidence or prevalence, with 2 detecting a reduction in incidence or prevalence associated with intervention [56,77], and the rest finding no difference between intervention and comparator arms [35,37,57,66,74]. In the studies observing improved outcomes, Kissinger et al [56] reported that the odds of reinfection among index cases were lower (OR 0.22, 95% CI 0.11-0.44), while Wilson et al [77] observed a 5% drop in reinfection rates of index cases (*P*=.02).

##### Unintended Consequences

A total of 2 studies assessed unintended consequences. Both were RCTs, 1 from South Africa and 1 from Kenya, and both found that the incidence of intimate partner violence was not affected by the intervention [27,66]. However, Mathews et al [66] observed that abandonment was slightly but significantly more likely following the intervention (risk difference 1.7%, 95% CI 0.2%-3.3%; *P*=.02).

#### Summary

This review included 35 prioritized studies focusing on CT interventions in STIs, including 13 in HIV. In all but 1 study, interventions were a form of PN, which was either provider- or patient-led. Such interventions typically incorporated the use of referral slips, CT, active follow-up, and self-testing kits.

**Figure 2:**
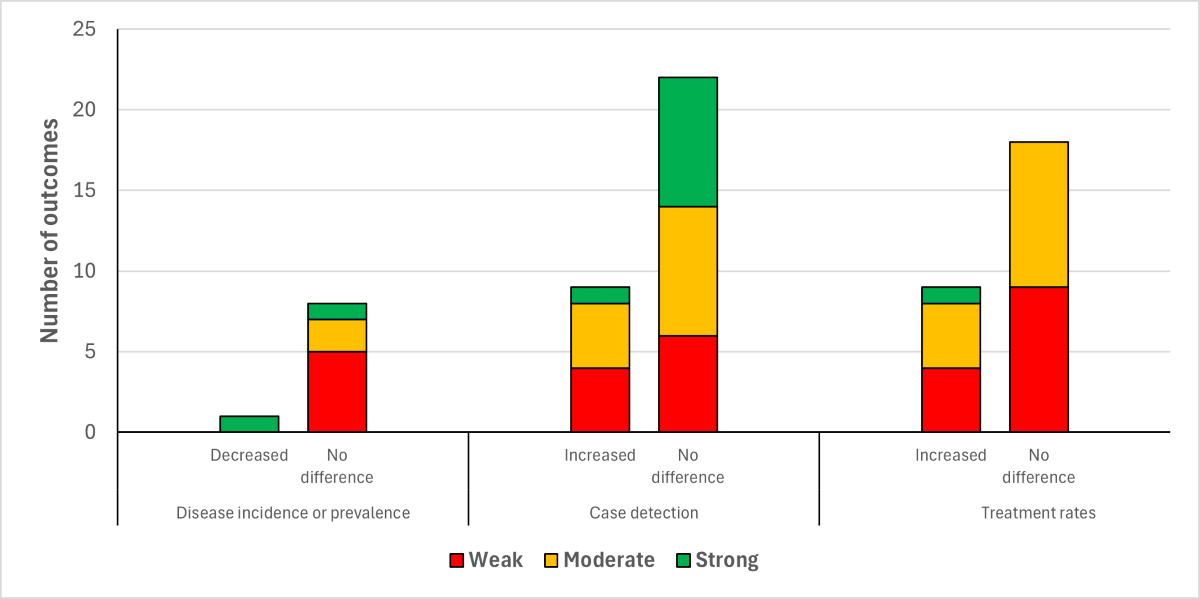
Distribution of effectiveness outcomes across the domains of disease incidence or prevalence, case detection, and treatment rates, for contact tracing (CT) in sexually transmitted or blood-borne infections. Color represents the quality rating of studies providing the outcome measure.

Despite the sizeable body of evidence, there was considerable heterogeneity across study design, intervention components, and outcomes evaluated, and there were only 5 studies rated as being of “strong” quality. Across all effectiveness outcome domains (case detection, disease incidence or prevalence, treatment rates, and other outcomes), it was most often observed that interventions had no effect on outcomes, though this was not exclusively the case, with several instances of improved outcomes, as depicted in Figure 2. Case detection, the most frequently assessed outcome domain, was typical of the pattern of results, featuring in 21 studies, with 7 interventions associated with greater case detection rates and the rest showing no differences compared with their comparators. There were only 2 instances in which outcomes related to unintended consequences were reported: 1 showing no difference between intervention and comparator for the risk of intimate partner violence, and 1 instance in which the intervention was reportedly associated with an unfavorable outcome—an increased likelihood of abandonment. Overall, there were 19 instances of improved outcomes across the 69 outcomes reported, with 1 negative outcome. Positive findings were more likely to be observed when provider referral was compared with patient referral.

There was little consistency in findings or patterns of intervention components that may be more likely to be associated with successful outcomes. The heterogeneity of the evidence, in terms of design (both at the study and intervention level), quality, and outcomes, precludes robust conclusions. However, the overall picture for CT in sexually transmitted or blood-borne diseases is one in which interventions either do no harm or may improve outcomes.

### Public and Patient Involvement and Engagement

The review was discussed 3 times with PERSPEX, our standing patient and public involvement and engagement group. This enabled the team to gain insights about CT from patient, carer, and public perspectives. In December 2024, the review team introduced the concept of CT and discussed its use as a public health intervention in general terms. Members drew on their experiences of COVID-19 CT. A majority opinion shared by members was that reviewers should not underestimate the concerns about, and lack of trust in, government agencies that are prevalent in some communities, which could affect the success of CT initiatives. Members were also concerned that some communities may experience additional barriers affecting the outcome of CT. This emphasized the importance of including health equity data in the review protocol. This was taken forward into the review and is available in the full report [10].

In February 2025, PERSPEX received a brief update on the progress of the review. In March 2025, the team reported the emergent main discussion items from the review. PERSPEX members raised a number of matters, which included a query about the role of technology in CT; a view that people respond in different ways to CT and not everyone sees the need to take action; and, for members of PERSPEX whose country of origin was outside the United Kingdom, discussion about the cost of medications even if they are traced and how this could affect the success of CT. Members also spoke about the taboos in many families and cultures around some illnesses and how this could hinder CT and subsequent management within the home. This discussion about culture fed into our thinking about the generalizability of interventions.

Some members shared their experiences of TB management requirements and visa entry for their relatives and visitors from South Asia and Africa. They also discussed UK maternity ward visitor restrictions aimed at preventing the spread of TB. These were shared as positive preventive measures, understood within the community and therefore indicative that CT may be positively received if supported by simultaneous patient education and delivered by community-based teams.

## Discussion

### Statement of Principal Findings

Our systematic review of the best available evidence relating to the effectiveness of CT identified 57 reports of 55 studies including randomized or prospective nonrandomized comparisons, interrupted time-series designs, or retrospective studies with concurrent controls. A sizeable evidence base drawing on uncontrolled pre-post comparisons was deprioritized given the extent of the higher-quality evidence identified. Included studies related to bacterial or parasitic STIs, HIV, TB, and COVID-19, covering only 2 relevant routes of transmission. Most studies compared different forms of CT against each other. More than half of the included studies had notable methodological limitations, raising an important question about the certainty of our conclusions, though no one critical appraisal domain was identified as driving this. In brief, included studies were inconclusive with respect to the impacts of CT in COVID-19 but provided some tentative evidence for reductions in disease incidence and prevalence in TB attributable to CT. Included studies focusing on STIs and HIV were similarly inconclusive, but some signal of improved effectiveness emerged in the use of provider-led referral vs patient-led referral. Interventions were broadly categorized as including technological methods, HI methods, PR, and PICT, with clear splits based on route of transmission. The heterogeneity of interventions, outcome definitions, and comparators precludes a clear assertion of effectiveness of any one CT strategy.

### Comparison With Previous Literature

Although there are some differences in the pool of evidence drawn upon, our findings are broadly in keeping with recent reviews that covered multiple conditions, led by Hossain et al [2] and Guy et al [3]. Hossain et al [2] focused on PICT as the intervention, with slight differences in definitions of CT and outcomes of interest, while Guy et al [3] included descriptive studies, leading to inclusion of nearly 380 studies, had a broad definition of CT, and included a focus on sociocultural factors influencing uptake and success of CT strategies. Ours was also the only review to consider the unintended consequences of CT. Despite these differences, all 3 reviews concluded that there was some promise for PICT interventions but that evidence was highly heterogeneous and of mixed quality. The varying foci of reviews provide complementary evidence in the quest to develop successful CT strategies suitable for a variety of infectious diseases, such as identifying CT strategies that may be successful across a variety of infectious diseases and learning about the important sociocultural factors influencing their uptake.

### Interpretation and Implications

#### Background Disease Context

We included conditions with epidemic or pandemic potential. In the case of COVID-19, all comparisons drew from a pandemic in progress. Evidence relating to TB generally drew from explicitly endemic contexts. However, a challenge in the evidence related to characterization of the “background” disease context; for example, whether STI CT was implemented in the context of a live outbreak. As a general point, it was nearly always unclear from non–COVID-19 studies how background disease surveillance specifically influenced the design of innovations in CT. Most studies resorted to appeals to health care system efficiency or general disease burden to justify why CT was evaluated.

#### Intervention Generalizability

Related to the above, a key challenge in the evidence was identifying how different approaches to CT might generalize to “true” pandemic contexts, or across conditions and routes of transmission. The substantial literature relating to bacterial STIs and HIV focused on PN, which implies a specific, and easily identifiable, form of “contact” that may be less relevant in a pandemic driven by airborne transmission, for example. Similarly, evidence relating to TB focused primarily on household contacts. Another facet of generalizability related to intervention context. Our review was global in nature, meaning we included substantial amounts of evidence from the Global South. A challenge, but also an opportunity, for public health in well-resourced settings will be to identify how innovations successful in low-income or middle-income country contexts might be relevant in the Global North [109]. Finally, generalizability may be impacted by the human capital, and other forms of capital, that CT strategies require. Many CT strategies included a focus on training and deploying community health care workers, disease intervention specialists, or community health nurses, or culturally embedded local volunteers. These may not be available at speed and in the required quantities in a pandemic context.

#### Outcomes

This review did not focus on contact yield as a key outcome, preferring instead measures of disease prevalence and incidence, transmission, and treatment, alongside a parallel focus on unintended consequences. Contact yield is an important outcome because it reflects engagement of contacts with public health services, but it does not capture meaningful impacts on health and well-being of contacts or on disease control. It is an important challenge to the field to consider why an outcome that is primarily about health system outputs, rather than population health impact, is so paramount. This is also important because anecdotally, even where interventions reported improved contact yield, this did not always translate to improved prevalence, incidence, transmission, or treatment outcomes.

About half of all studies in this review reported on a case detection–specific outcome, with treatment rates among contacts a close second. Treatment rates were particularly well described in evidence from CT for bacterial or parasitic STIs but will be less relevant for infectious diseases where treatments either do not exist or are not widely available. The ultimate test of CT effectiveness is whether it reduces, or even eliminates, longer-term incidence or prevalence of the condition, or whether it reduces morbidity or mortality where this is a risk. These outcomes were comparatively rare, and mortality analyses specifically were linked to TB and COVID-19 evidence. In addition, the link between treatment rates and improved mortality is driven by adherence to treatment regimens. In conditions such as TB that require extended courses of therapy, CT-driven improvements in treatment uptake may not translate into improvements in incidence, prevalence, and disease-specific mortality [110].

#### Unintended Consequences

An innovative feature of our review was the inclusion of unintended consequences of CT. It is notable that despite an exhaustive search for a wide range of unintended consequences, only 2 studies presented evidence in this respect. These studies evaluated CT in sexual health contexts and focused on intimate partner violence or relationship breakdown, with neither finding a difference in intimate partner violence but one finding an increase in relationship abandonment attributable to PN. While these are important, it is likely that the generalizability of these findings to other disease contexts is limited. A broader focus on the potential unintended consequences of health and social interventions is important to fully account for the health impacts of CT, especially for conditions with airborne transmission. For example, in situations where enhanced enrollment into TB treatment is rolled out because of CT, suboptimal concordance with treatment regimens may amplify the challenge of antimicrobial resistance in communities [111].

### Unanswered Questions and Future Research

CT strategies in a future pandemic will need to be designed from an understanding of disease transmission pathways. In this light, then, what is the relevance of CT strategies for other conditions, particularly given that we were only able to identify relevant evidence for 2 routes of transmission? Learning from CT for STIs and HIV could support management of stigma and linkage into treatment, and the use of contact-delivered testing where feasible. Learning from CT for TB could support appropriate services for household contacts, especially where those household contacts might be more susceptible. Finally, learning from CT for COVID-19 could focus on rapidly accelerating CT in the context of a pandemic, in addition to management of contacts where these are an ill-defined set. An important consideration for policymakers and commissioners will be to identify how innovations in CT could support new approaches for conditions, including how these relate to learning from the Global South.

This review will be used to inform the development of UK Health Security Agency advice and guidance as part of pandemic preparedness and to help inform research priorities and future evaluation and improvement approaches to CT. For future preparedness, this means developing flexible CT strategies tailored to specific diseases, investing in higher-quality evaluation methods, and ensuring fairness considerations are built into program design from the outset.

### Strengths and Limitations of the Review

An extensive and comprehensive search, selection, and appraisal were undertaken to the highest standards, though as with any systematic review, eligible studies may have been missed. Due to the quantity of identified evidence, we prioritized evidence from evaluations with a concurrent control. Although this was a post hoc decision, we believe this strengthened analysis because it focused on the best available evidence, and the deprioritized studies provided no unique coverage. As a general point, while CT is often presented as a highly prescriptive, medicalized intervention, it was not our experience that this was reflected in included studies. This was both in situations where CT was bundled as part of multicomponent interventions and where specific comparisons undertaken in the context of CT were judged by our team as not relevant. An example of this is partner-delivered treatment, which is largely applicable to bacterial STIs alone. In addition, studies compared CT against heterogeneous comparators, such as usual care and other forms of CT. Usual care was frequently described in scant detail, precluding precision in defining the comparator. Some element of subjectivity, shaped by the challenging and complex nature of CT interventions and their descriptions, may mean that an alternative review group would have included different studies with possibly different overall conclusions. However, we have sought to mitigate this with an auditable and comprehensive account of how judgments were undertaken throughout the review process.

### Conclusion

CT is a mainstay of public health response to infectious diseases. Our review found inconsistent evidence for the effectiveness of CT, focused primarily on TB and on contrasts between PICT and patient-led referral in STIs and HIV. While most outcomes did not demonstrate a difference between CT and the comparator intervention, there was some evidence of reductions in disease prevalence in TB and for PICT to be superior to patient-led approaches in STIs. These results do not necessarily reflect a lack of effectiveness of CT strategies. However, they highlight the need to build evaluation into the implementation of CT strategies to improve evidence generation. Heterogeneity of intervention, comparator, and outcome precluded any clear assertions as to optimal strategies for CT, including with respect to relevant subgroups. Future work should consider generalizability of learning across contexts, including by route of transmission and from the Global South; a clearer focus on health equity; and a more thorough account of unintended consequences of CT.

## Appendix

Multimedia Appendix 1 PRISMA checklist.

Multimedia Appendix 2 Search strategy.

Multimedia Appendix 3 Inclusion criteria.

Multimedia Appendix 4 Reasons for exclusion at full text.

Multimedia Appendix 5 Search summary table.

Multimedia Appendix 6 Study details.

Multimedia Appendix 7 Tables relating to airborne transmission.

Multimedia Appendix 8 Tables relating to sexual or bloodborne transmission.

## Abbreviations

CT: contact tracing
DOTS: directly observed treatment, short-course
HI: household investigation
IRR: incidence rate ratio
ISSG: InterTASC Information Specialists’ Sub-Group
MeSH: Medical Subject Headings
OR: odds ratio
PERSPEX: Public Engagement in Research for Health and Social Policy at Exeter
PHSM: Public Health and Social Measure
PICT: provider-initiated contact tracing
PN: partner notification
PR: partner referral
PRISMA: Preferred Reporting Items for Systematic Reviews and Meta-Analyses
PROSPERO: International Prospective Register of Systematic Reviews
RCT: randomized controlled trial
RR: risk ratio
STI: sexually transmitted infection
TB: tuberculosis
TECH: technology-based contact tracing
WHO: World Health Organization

## Data Availability

The datasets generated or analyzed during this study are available from the corresponding author on reasonable request.
