# Supplementary material for: The Effectiveness of Contact Tracing to Reduce Transmission of Infectious Diseases During Epidemic or Pandemic Response: Rapid Systematic Review
Source: JMIR Public Health Surveill. 2026 Mar 31;12:e84805. doi: 10.2196/84805 (PMC13080299; doi:10.2196/84805)
Supplement: Multimedia Appendix 2 [file publichealth_v12i1e84805_app2.docx]

## Search strategy

Ovid MEDLINE(R) ALL <1946 to November 14, 2024>

1 (contact* adj2 (tracing or trace*)).ti. 1439

2 (contact* adj2 investigat*).ti. 457

3 (contact* adj2 examin*).ti. 65

4 (contact* adj2 screen*).ti. 130

5 (partner* adj2 (notice* or notif*)).ti. 492

6 (contact* adj2 follow-up*).ti. 42

7 1 or 2 or 3 or 4 or 5 or 6 2550

8 (contact* adj2 (tracing or trace*)).ab. 4776

9 (contact* adj2 investigat*).ab. 2766

10 (contact* adj2 examin*).ab. 998

11 (contact* adj2 screen*).ab. 848

12 (contact* adj2 followup).ab. 15

13 (contact* adj2 "follow up").ab. 1109

14 (partner* adj2 (notice* or notif*)).ab. 1106

15 (household adj2 contact*).ti. 840

16 Contact Tracing/ 6531

17 or/8-16 15252

18 *Disease Outbreaks/pc [Prevention & Control] 8056

19 epidemics/ or pandemics/ 149826

20 pandemic*.tw. 248441

21 epidemic*.tw. 144929

22 *sexually transmitted diseases/ or *sexually transmitted diseases, bacterial/ or *sexually transmitted diseases, viral/ 22593

23 (sexually transmitted adj1 (infection* or disease*)).tw. 35638

24 (sti?1 or std?1).tw. 2561

25 Syphilis/pc [Prevention & Control] 1518

26 *Syphilis/ 20137

27 Syphilis.tw. 29746

28 *chlamydia/ 2413

29 *Chlamydia trachomatis/ 8464

30 chlamydia*.tw. 31321

31 COVID-19/pc [Prevention & Control] 40148

32 *COVID-19/ 230009

33 covid*.tw. 387162

34 (corona adj1 vir*).tw. 3632

35 coronav*.tw. 141907

36 coronov*.tw. 92

37 *Severe acute respiratory syndrome-related coronavirus/ 3245

38 *SARS-CoV-2/ 37836

39 sars*.tw. 142613

40 Severe acute respiratory syndrome.tw. 46924

41 HIV Infections/pc [Prevention & Control] 45110

42 *HIV Infections/ 203613

43 hiv*.tw. 372216

44 human immunodeficiency virus*.tw. 98720

45 Measles/pc [Prevention & Control] 6375

46 *Measles/ 12191

47 *Measles virus/ 4496

48 measles.tw. 26749

49 *Trichomonas/ 1458

50 Trichomonas Infections/pc [Prevention & Control] 90

51 *Trichomonas Infections/ 2859

52 Trichomon*.tw. 10610

53 Tuberculosis/pc [Prevention & Control] 13952

54 *Tuberculosis/ 106121

55 tuberculosis.tw. 222223

56 tb.tw. 79965

57 Influenza, Human/pc [Prevention & Control] 21460

58 *Influenza, Human/ 51795

59 influ*.tw. 2178055

60 flu.tw. 17618

61 exp Influenza A virus/ 51167

62 H5N1.tw. 7617

63 H1*.tw. 70102

64 Hemorrhagic Fever, Ebola/pc [Prevention & Control] 2357

65 *Hemorrhagic Fever, Ebola/ 6053

66 ebola*.tw. 11419

67 Poxviridae Infections/pc [Prevention & Control] 241

68 *Poxviridae Infections/ 1771

69 "Mpox (monkeypox)"/pc [Prevention & Control] 386

70 *"Mpox (monkeypox)"/ 2790

71 mpox*.tw. 1961

72 pox*.tw. 11573

73 monkeypox.tw. 4169

74 porcine*.tw. 100758

75 Hemorrhagic Fever, Crimean/pc [Prevention & Control] 141

76 *Hemorrhagic Fever, Crimean/ 1229

77 Crimean Congo h?emorrhagic fever*.tw. 2018

78 cchf*.tw. 1499

79 Marburg Virus Disease/pc [Prevention & Control] 153

80 *Marburg Virus Disease/ 451

81 Marburgvirus/ 780

82 marburg*.tw. 2977

83 Lassa Fever/pc [Prevention & Control] 199

84 *Lassa Fever/ 745

85 lassa*.tw. 1973

86 Rift Valley Fever/pc [Prevention & Control] 255

87 *Rift Valley Fever/ 1225

88 rift valley fever*.tw. 2436

89 rvf*.tw. 3259

90 Middle East Respiratory Syndrome Coronavirus/ 2152

91 Middle East respiratory syndrome*.tw. 3769

92 mers*.tw. 11043

93 Nipah Virus/ 749

94 nipah*.tw. 1536

95 niv.tw. 4964

96 Henipavirus Infections/pc [Prevention & Control] 171

97 *Henipavirus Infections/ 644

98 henipavir*.tw. 514

99 Zika Virus Infection/pc [Prevention & Control] 1229

100 *Zika Virus Infection/ 6848

101 *Zika Virus/ 5709

102 zika*.tw. 12235

103 exp Dengue/pc [Prevention & Control] 3135

104 *Dengue/ 13682

105 *Dengue Virus/ 8725

106 dengu*.tw. 30018

107 Chikungunya Fever/pc [Prevention & Control] 361

108 *Chikungunya Fever/ 2409

109 *Chikungunya virus/ 2774

110 Chikungunya*.tw. 7719

111 exp Shigella/ 12509

112 Shigella*.tw. 16707

113 exp Norovirus/ 5925

114 norovir*.tw. 7661

115 or/18-114 3676309

116 "evaluation study".pt. or "evaluation studies as topic"/ or program evaluation/ or "reproducibility of results"/ or "validation study".pt. or "validation studies as topic"/ or (((pre adj5 test) and (post adj5 test)) or (pretest adj5 posttest) or ((intervention or nonrandomized or program* or (quasi adj1 experimental) or randomised or randomized) adj5 (effectiveness or evaluate or evaluated or evaluates or evaluating or evaluation or evaluations or evaluator or evaluators or reliability or reproducibility or validity))).ti,ab,kf,kw. 1089224

***(From: https://hsls.libguides.com/Ovid-Medline-search-filters/experimental-studies)***

117 Epidemiologic Methods/ or exp Epidemiologic Studies/ or Observational Studies as Topic/ or Clinical Studies as Topic/ or (Observational Study or Validation Studies or Clinical Study).pt. or (observational adj3 (study or studies or design or analysis or analyses)).ti,ab,kf. or cohort*.ti,ab,kf. or (prospective adj7 (study or studies or design or analysis or analyses)).ti,ab,kf. or ((follow up or followup) adj7 (study or studies or design or analysis or analyses)).ti,ab,kf. or ((longitudinal or longterm or (long adj term)) adj7 (study or studies or design or analysis or analyses or data)).ti,ab,kf. or ((case adj control) or (case adj comparison) or (case adj controlled)).ti,ab,kf. or (case-referent adj3 (study or studies or design or analysis or analyses)).ti,ab,kf. or (population adj3 (study or studies or analysis or analyses)).ti,ab,kf. or (descriptive adj3 (study or studies or design or analysis or analyses)).ti,ab,kf. or ((multidimensional or (multi adj dimensional)) adj3 (study or studies or design or analysis or analyses)).ti,ab,kf. or (quasi adj (experiment or experiments or experimental)).ti,ab,kf. or ((non experiment or nonexperiment or non experimental or nonexperimental) adj3 (study or studies or design or analysis or analyses)).ti,ab,kf. 4511482

***(From:*** [***https://searchfilters.cadth.ca/list?q=&p=1&ps=20&name_facet=medline%20000000%7CMEDLINE***](https://searchfilters.cadth.ca/list?q=&p=1&ps=20&name_facet=medline%20000000%7CMEDLINE) 
***Edits: single-case studies as topic/ or case reports as topic/ or (retrospective adj7 (study or studies or design or analysis or analyses or data or review)).ti,ab,kf or (cross adj sectional adj7 (study or studies or design or research or analysis or analyses or survey or findings)).ti,ab,kf or ((natural adj experiment) or (natural adj experiments)).ti,ab,kf or (prevalence adj3 (study or studies or analysis or analyses)).ti,ab,kf. Deleted from the filter)***

118 "Controlled Before-After Studies"/ or "Interrupted Time Series Analysis"/ or ((interrupted adj time) or (non adj (randomised or randomized)) or nonequivalent or nonrandomised or nonrandomized).ti,ab,kf,kw. or ((one adj group) and (((post or pre) adj test) or pretest or posttest)).ti,ab,kf,kw. or (((pretest or (pre adj5 (intervention or posttest or test))) and (posttest or (post adj5 (intervention or test)))) or (pretest adj5 posttest) or quasiexperimental or (quasi adj experimental) or ((single adj group) and (group adj study)) or (uncontrolled adj (studies or study))).ti,ab,kf,kw. or intervention*.tw. or effectiv*.tw. 4024288

***(From:*** [***https://hsls.libguides.com/Ovid-Medline-search-filters/experimental-studies***](https://hsls.libguides.com/Ovid-Medline-search-filters/experimental-studies)

***Edits: intervention.ti to intervention*.tw or effective*.tw)***

119 exp randomized controlled trial/ 627195

120 controlled clinical trial.pt. 95633

121 randomi?ed.ab. 796493

122 placebo.ab. 253434

123 clinical trials as topic/ 203763

124 randomly.ab. 446110

125 trial.ti. 322108

***(From: ISSG Search Filter Resource [Internet].  Glanville J, Lefebvre C, Manson P, Robinson S, Brbre I and Woods L, editors.  York (UK):  The InterTASC Information Specialists' Sub-Group; 2006 [updated 27 October 2024; cited 27 October 2024].)***

126 119 or 120 or 121 or 122 or 123 or 124 or 125 1690741

127 116 or 117 or 118 or 126 9107704

128 17 and 115 and 127 5500

129 7 or 128 6876

130 exp animals/ not humans/ 5275914

131 129 not 130 6834
