# Supplementary material for: The Effectiveness of Contact Tracing to Reduce Transmission of Infectious Diseases During Epidemic or Pandemic Response: Rapid Systematic Review
Source: JMIR Public Health Surveill. 2026 Mar 31;12:e84805. doi: 10.2196/84805 (PMC13080299; doi:10.2196/84805)
Supplement: Multimedia Appendix 3 [file publichealth_v12i1e84805_app3.docx]

## Inclusion and exclusion criteria

|  | **Included** | **Excluded** |
| --- | --- | --- |
| **Population** | All (adults, children and young people) |  |
| **Context** | Outbreak, epidemic or pandemic (or potential for) of infectious disease. All routes of transmission to be considered separately:   - Respiratory - Vector borne - Direct borne - Blood borne - Food/water borne (ingestion) | CT using outside of outbreak, epidemic, or pandemic response. CT used outside of outbreak, epidemic, or pandemic response. CT used in nosocomial outbreaks specifically (i.e. in healthcare settings where the goal is outbreak control in that setting) |
| **Settings** | Community (include CT that is led by a healthcare setting, but not limited to that healthcare setting) | Healthcare settings where the goal is control of nosocomial outbreaks |
| **Intervention or exposure** | CT | Any other PHSM, or intervention to reduce transmission that does not incorporate CT |
| **Comparator** | - Different CT approaches compared to each other - No CT - CT and active monitoring (where contacts deemed to be at high risk of developing an infection are monitored and asked to report to health protection teams, whether or not they have symptoms) compared to CT and passive monitoring (where contacts monitor their own symptoms and only report to health protection teams if they become symptomatic) | Other PHSM where these are not part of background measures (i.e. shared equally in both arms) |
| **Outcomes** | **Effectiveness:**   - Any measure of transmission of the focal disease, including:   - Case detection rates   - Reproduction number   - Secondary attack rates   - Cases or deaths averted   - Reinfection rates among contacts   - Treatment rates among contacts   - Overall disease incidence or prevalence - Healthcare use related to the focal disease (for example, admission to A&E or ICU) - Mortality   **Unintended consequences:**   - Outcome measures of population health - Change in incidence, prevalence, attack rates, and/or mortality for diseases other than the PHSM is intended for. - Morbidity outcomes directly or indirectly related to implementation of the PHSM. For example, mobility, pain or discomfort, voice parameters (e.g., perceptual, acoustic, aerodynamic, physiological), skin conditions. - Mental health outcomes. For example, anxiety, depression, stress, post-traumatic stress disorder, disordered eating, suicide, suicidal ideation. - Individual risk factors for poor health - substance use, violence/ domestic violence, sleep disturbance or other sleep related issues, reduced physical activity or increase in sedentary behaviour, nutritional status, diet or body mass index, loneliness - Social determinants of health - Social cohesion, early childhood development, educational attainment, number of days spent in school, absenteeism, and disability. Increased exposure to risk factors such as sexual abuse and criminal exploitation in children and young people, widening social inequalities like gender disparities. - Economic outcomes such as household or individual economic hardship, poverty, food insecurity, homelessness or access to housing, unemployment, or job insecurity - Health service utilisation patterns not related to the focal disease, i.e. attendance at routine appointments, or changes in admission to A&E, hospital or ICU | The following unintended consequences:   - Macroeconomic outcomes - Metrics at a national or international scale, that are not regarded as a risk factor for poor health to an individual or a social determinant of health. For example, economic productivity or growth, GDP, national income, inflation, widening economic inequalities - Environmental or ecological outcomes. For example transportation, parks, playgrounds. - Political outcomes. For example public trust, political polling. - Legal or human rights related outcomes. For example, crime rates. - Technological outcomes. For example, development and use of technology tools, telehealth, use of artificial intelligence. |
| **Language** | English | Any other language |
| **Date of publication** | No limit |  |
| **Study design** | Experimental studies with a control group, including:   - randomised-controlled trials - quasi-experimental studies - before-and-after studies   Observational studies with a control group, including:   - cross-sectional - case-control cohort | Studies without control groups  Systematic (or rapid) reviews  Narrative reviews / literature reviews  Cross-over study designs  Modelling studies  Case series  Case reports  Qualitative studies |
| **Publication type** | Peer-reviewed research published at full text | Preprints  Conference abstracts  Editorials  Letters  News articles  Grey literature  Protocols |
| A&E, accident and emergency; CT, contact tracing; GDP, gross domestic product; ICU, intensive care unit; PHSM, public health and social measures. | | |
