# Supplementary material for: The Effectiveness of Contact Tracing to Reduce Transmission of Infectious Diseases During Epidemic or Pandemic Response: Rapid Systematic Review
Source: JMIR Public Health Surveill. 2026 Mar 31;12:e84805. doi: 10.2196/84805 (PMC13080299; doi:10.2196/84805)
Supplement: Multimedia Appendix 4 [file publichealth_v12i1e84805_app4.docx]

# Table of studies excluded at full text, with reasons (N=134)

|  | **Studies** | **Reason for exclusion** |
| --- | --- | --- |
| 1. | Admiraal R, Millen J, Patel A, Chambers T. A Case Study of Bluetooth Technology as a Supplemental Tool in Contact Tracing. *J Healthc Inform Res;*6:208-27. https://doi.org/https://dx.doi.org/10.1007/s41666-021-00112-9 | Disease / condition |
| 2. | Chambers T, Anglemyer A. Pilot of a digital contact tracing card in a hospital setting in New Zealand, 2020. *J Public Health (Oxf)* 2023;45:e171-e4. https://doi.org/https://dx.doi.org/10.1093/pubmed/fdac045 | Disease / condition |
| 3. | Shelby T, Caruthers T, Kanner OY, Schneider R, Lipnickas D, Grau LE*, et al.* Pilot Evaluations of Two Bluetooth Contact Tracing Approaches on a University Campus: Mixed Methods Study. *JMIR formative research* 2021;5:e31086. https://doi.org/https://dx.doi.org/10.2196/31086 | Disease / condition |
|  |  |  |
| 1. | Baik Y, Hanrahan CF, Mmolawa L, Nonyane BAS, Albaugh NW, Lebina L*, et al.* Conditional Cash Transfers to Incentivize Tuberculosis Screening: Description of a Novel Strategy for Contact Investigation in Rural South Africa. *Clin infect dis* 2022;74:957-64. https://doi.org/https://dx.doi.org/10.1093/cid/ciab601 | Intervention |
| 2. | Balakrishnan S, Ps R, M S, Sankar B, Ramachandran R, Ka A*, et al.* STEPS: A Solution for Ensuring Standards of TB Care for Patients Reaching Private Hospitals in India. *Global health, science and practice* 2021;9:286-95. https://doi.org/https://dx.doi.org/10.9745/GHSP-D-20-00449 | Intervention |
| 3. | Chayachinda C, Kerdklinhom C, Tachawatcharapunya S, Saisaveoy N. Video-based education versus nurse-led education for partner notification in Thai women with sexually transmitted infections: a randomized controlled trial. *Int J STD AIDS* 2018;29:1076-83. https://doi.org/https://dx.doi.org/10.1177/0956462418775507 | Intervention |
| 4. | Corbett EL, Bandason T, Duong T, Dauya E, Makamure B, Churchyard GJ*, et al.* Comparison of two active case-finding strategies for community-based diagnosis of symptomatic smear-positive tuberculosis and control of infectious tuberculosis in Harare, Zimbabwe (DETECTB): A cluster-randomised trial. *The Lancet* 2010;376:1244-53. https://doi.org/10.1016/S0140-6736(10)61425-0 | Intervention |
| 5. | Davis JL, Turimumahoro P, Meyer AJ, Ayakaka I, Ochom E, Ggita J*, et al.* Home-based tuberculosis contact investigation in Uganda: a household randomised trial. *ERJ open research* 2019;5. https://doi.org/https://dx.doi.org/10.1183/23120541.00112-2019 | Intervention |
| 6. | Ekwueme O-EC, Omotowo BI, Agwuna KK. Strengthening contact tracing capacity of pulmonary tuberculosis patients in Enugu, southeast Nigeria: a targeted and focused health education intervention study. *BMC Public Health* 2014;14:1175. https://doi.org/https://dx.doi.org/10.1186/1471-2458-14-1175 | Intervention |
| 7. | Fox GJ, Sy DN, Hoa NLP, Anh LTN, Anh NT, Hoa NB*, et al.* Household-contact investigation for detection of tuberculosis in Vietnam. *N Engl J Med* 2018;378:221-9. https://doi.org/10.1056/NEJMoa1700209 | Intervention |
| 8. | Gannon-Loew KE, Holland-Hall C, Ebersole AM, Alexy E, Jackson K, Bonny AE. Expedited Partner Therapy in Female Adolescents: A Study of Acceptance and the Impact on Reinfection Rates. *Sex Transm Dis* 2021;48:828-33. https://doi.org/https://dx.doi.org/10.1097/OLQ.0000000000001436 | Intervention |
| 9. | Hirsch-Moverman Y, Howard AA, Mantell JE, Lebelo L, Frederix K, Wills A*, et al.* Improving child tuberculosis contact identification and screening in Lesotho: Results from a mixed-methods cluster-randomized implementation science study. *PLoS One* 2021;16:e0248516. https://doi.org/https://dx.doi.org/10.1371/journal.pone.0248516 | Intervention |
| 10. | Hoxworth T, Hoffman R, Cohn D, Davidson A. Anonymous HIV testing: does it attract clients who would not seek confidential testing? *AIDS Public Policy J* 1994;9:182-9. | Intervention |
| 11. | Kaku JS, Ahmad RA, Main S, Oktofiana D, Dwihardiani B, Triasih R*, et al.* Tuberculosis Case Finding in Kulon Progo District, Yogyakarta, Indonesia: Passive versus Active Case Finding Using Mobile Chest X-ray. *Tropical Medicine and Infectious Disease* 2024;9. https://doi.org/10.3390/tropicalmed9040075 | Intervention |
| 12. | Kanu FA, Smith EE, Offutt-Powell T, Hong R, Dinh T-H, Pevzner E. Declines in SARS-CoV-2 Transmission, Hospitalizations, and Mortality After Implementation of Mitigation Measures- Delaware, March-June 2020. *MMWR Morbidity and mortality weekly report* 2020;69:1691-4. https://doi.org/https://dx.doi.org/10.15585/mmwr.mm6945e1 | Intervention |
| 13. | Karamagi E, Sensalire S, Muhire M, Kisamba H, Byabagambi J, Rahimzai M*, et al.* Improving TB case notification in northern Uganda: evidence of a quality improvement-guided active case finding intervention. *BMC Health Serv Res* 2018;18:954. https://doi.org/https://dx.doi.org/10.1186/s12913-018-3786-2 | Intervention |
| 14. | Karimi A, Kadivar MR, Fararoee M, Alborzi A. Active case-finding of communicable diseases in the south of the Islamic Republic of Iran. *East Mediterr Health J* 2000;6:487-93. | Intervention |
| 15. | Keita M, Polonsky JA, Ahuka-Mundeke S, Ilumbulumbu MK, Dakissaga A, Boiro H*, et al.* A community-based contact isolation strategy to reduce the spread of Ebola virus disease: an analysis of the 2018-2020 outbreak in the Democratic Republic of the Congo. *BMJ global health* 2023;8. https://doi.org/https://dx.doi.org/10.1136/bmjgh-2023-011907 | Intervention |
| 16. | Kumwenda A, Weideman AMK, Graybill LA, Dinwiddie MK, Freeborn K, Lusaka MM*, et al.* Two strategies for partner notification and partner HIV self-testing reveal no evident predictors of male partner HIV testing in antenatal settings: A secondary analysis. *Int J STD AIDS* 2023;34:1004-11. https://doi.org/https://dx.doi.org/10.1177/09564624231188746 | Intervention |
| 17. | Loredo C, Cailleaux-Cezar M, Efron A, de Mello FCQ, Conde MB. Yield of close contact tracing using two different programmatic approaches from tuberculosis index cases: a retrospective quasi-experimental study. *BMC Pulm Med* 2014;14:133. https://doi.org/https://dx.doi.org/10.1186/1471-2466-14-133 | Intervention |
| 18. | Lu LC, Ouyang D, D'Agostino A, Diaz A, Rudman SL, Ho DE. Integrating social services with disease investigation: A randomized trial of COVID-19 high-touch contact tracing. *PLoS One* 2023;18:e0285752. https://doi.org/https://dx.doi.org/10.1371/journal.pone.0285752 | Intervention |
| 19. | Lung T, Marks GB, Nhung NV, Anh NT, Hoa NLP, Anh LTN*, et al.* Household contact investigation for the detection of tuberculosis in Vietnam: economic evaluation of a cluster-randomised trial. *The Lancet Global health* 2019;7:e376-e84. https://doi.org/https://dx.doi.org/10.1016/S2214-109X(18)30520-5 | Intervention |
| 20. | Menzies D, Obeng J, Hadisoemarto P, Ruslami R, Adjobimey M, Fisher D*, et al.* Sustainability and impact of an intervention to improve initiation of tuberculosis preventive treatment: results from a follow-up study of the ACT4 randomized trial. *EClinicalMedicine* 2024;71:102546. https://doi.org/10.1016/j.eclinm.2024.102546 | Intervention |
| 21. | Miller WC, Rutstein SE, Phiri S, Kamanga G, Nsona D, Pasquale DK*, et al.* Randomized Controlled Pilot Study of Antiretrovirals and a Behavioral Intervention for Persons with Acute HIV Infection: Opportunity for Interrupting Transmission. *Open Forum Infectious Diseases* 2019;6. https://doi.org/10.1093/ofid/ofy341 | Intervention |
| 22. | Nuwaha F, Kambugu F, Nsubuga PS, Hojer B, Faxelid E. Efficacy of patient-delivered partner medication in the treatment of sexual partners in Uganda. *Sex Transm Dis* 2001;28:105-10. https://doi.org/https://dx.doi.org/10.1097/00007435-200102000-00008 | Intervention |
| 23. | Oxlade O, Benedetti A, Adjobimey M, Alsdurf H, Anagonou S, Cook VJ*, et al.* Effectiveness and cost-effectiveness of a health systems intervention for latent tuberculosis infection management (ACT4): a cluster-randomised trial. *The Lancet Public health* 2021;6:e272-e82. https://doi.org/https://dx.doi.org/10.1016/S2468-2667(20)30261-9 | Intervention |
| 24. | Page-Shipp L, Lewis JJ, Velen K, Senoge S, Zishiri E, Popane F*, et al.* Household point of care CD4 testing and isoniazid preventive therapy initiation in a household TB contact tracing programme in two districts of South Africa. *PLoS One* 2018;13:e0192089. https://doi.org/https://dx.doi.org/10.1371/journal.pone.0192089 | Intervention |
| 25. | Park Y, Huh IS, Lee J, Kang CR, Cho SI, Ham HJ*, et al.* Application of Testing-Tracing-Treatment Strategy in Response to the COVID-19 Outbreak in Seoul, Korea. *J Korean Med Sci* 2020;35:e396. https://doi.org/https://dx.doi.org/10.3346/jkms.2020.35.e396 | Intervention |
| 26. | Potty RS, Kumarasamy K, Adepu R, Reddy RC, Singarajipura A, Siddappa PB*, et al.* Community health workers augment the cascade of TB detection to care in urban slums of two metro cities in India. *Journal of global health* 2021;11:04042. https://doi.org/https://dx.doi.org/10.7189/jogh.11.04042 | Intervention |
| 27. | Reynolds SL, Kapadia AS, Leonard L, Ross MW. Examining the direct costs and effectiveness of syphilis detection by selective screening and partner notification. *J Public Health Med* 2001;23:339-45. https://doi.org/https://dx.doi.org/10.1093/pubmed/23.4.339 | Intervention |
| 28. | Rogers JH, Link AC, McCulloch D, Brandstetter E, Newman KL, Jackson ML*, et al.* Characteristics of COVID-19 in Homeless Shelters : A Community-Based Surveillance Study. *Ann Intern Med* 2021;174:42-9. https://doi.org/https://dx.doi.org/10.7326/M20-3799 | Intervention |
| 29. | Rose SB, Garrett SM, Hutchings D, Lund K, Kennedy J, Pullon SRH. Addressing Gaps in the Management of Chlamydia trachomatis and Neisseria gonorrhoeae in Primary Care: Lessons Learned in a Pilot Intervention Study. *Sex Transm Dis* 2019;46:480-6. https://doi.org/https://dx.doi.org/10.1097/OLQ.0000000000001005 | Intervention |
| 30. | Rosenbaum JE, Jennings J, Ellen JM, Borkovic LM, Scott JA, Wylie C*, et al.* Giving syphilis and gonorrhea to friends: using in-person friendship networks to find additional cases of gonorrhea and syphilis. *BMC Public Health* 2020;20:1526. https://doi.org/https://dx.doi.org/10.1186/s12889-020-09589-2 | Intervention |
| 31. | Rwabiyago OE, Katale A, Bingham T, Grund JM, Machangu O, Medley A*, et al.* Social network strategy (SNS) for HIV testing: a new approach for identifying individuals with undiagnosed HIV infection in Tanzania. *AIDS Impact Stockholm Sweden Special Issue: Power for action - now!* 2024;36:201-10. https://doi.org/https://dx.doi.org/10.1080/09540121.2024.2307383 | Intervention |
| 32. | Salazar-Austin N, Cohn S, Barnes GL, Tladi M, Motlhaoleng K, Swanepoel C*, et al.* Improving Tuberculosis Preventive Therapy Uptake: A Cluster-randomized Trial of Symptom-based Versus Tuberculin Skin Test-based Screening of Household Tuberculosis Contacts Less Than 5 Years of Age. *Clin Infect Dis* 2020;70:1725-32. https://doi.org/https://dx.doi.org/10.1093/cid/ciz436 | Intervention |
| 33. | Schillinger JA, Kissinger P, Calvet H, Whittington WLH, Ransom RL, Sternberg MR*, et al.* Patient-delivered partner treatment with azithromycin to prevent repeated Chlamydia trachomatis infection among women: A randomized, controlled trial. *Sex Transm Dis* 2003;30:49-56. https://doi.org/10.1097/00007435-200301000-00011 | Intervention |
| 34. | Solsona J, Cayla JA, Verdu E, Estrada MP, Garcia S, Roca D*, et al.* Molecular and conventional epidemiology of tuberculosis in an inner city district. *Int J Tuberc Lung Dis* 2001;5:724 - 31. | Intervention |
| 35. | Swayne JB, Tepper L. TUBERCULOSIS CONTACT FOLLOW-UP IN THE LOS ANGELES CITY HEALTH DEPARTMENT. *American journal of public health and the nation's health* 1964;54:1270-81. https://doi.org/https://dx.doi.org/10.2105/ajph.54.8.1270 | Intervention |
| 36. | Taylor MM, Reilley B, Yellowman M, Anderson L, de Ravello L, Tulloch S. Use of expedited partner therapy among chlamydia cases diagnosed at an urban Indian health centre, Arizona. *Int J STD AIDS* 2013;24:371-4. https://doi.org/https://dx.doi.org/10.1177/0956462412472825 | Intervention |
| 37. | Turyahabwe S, Bamuloba M, Mugenyi L, Amanya G, Byaruhanga R, Imoko JF*, et al.* Community tuberculosis screening, testing and care, Uganda. *Bull World Health Organ* 2024;102:400-9. https://doi.org/https://dx.doi.org/10.2471/BLT.23.290641 | Intervention |
| 38. | Wolde HM, Zerihun B, Sinshaw W, Yewhalaw D, Abebe G. Comparison of the yield of two tuberculosis screening approaches among household contacts in a community setting of Silti Zone, Central Ethiopia: a prospective cohort study. *BMC Pulm Med* 2024;24:135. https://doi.org/https://dx.doi.org/10.1186/s12890-024-02950-w | Intervention |
| 39. | Yanes-Lane M, Trajman A, Bastos ML, Oxlade O, Valiquette C, Rufino N*, et al.* Effects of programmatic interventions to improve the management of latent tuberculosis: a follow up study up to five months after implementation. *BMC Public Health* 2021;21:177. https://doi.org/https://dx.doi.org/10.1186/s12889-021-10195-z | Intervention |
| 40. | Young T, de Kock A, Jones H, Altini L, Ferguson T, van de Wijgert J. A comparison of two methods of partner notification for sexually transmitted infections in South Africa: patient-delivered partner medication and patient-based partner referral. *Int J STD AIDS* 2007;18:338-40. <https://doi.org/https://dx.doi.org/10.1258/095646207780749781> | Intervention |
|  |  |  |
| 1. | Al Kuwari MG, Al Nuaimi AA, Semaan S, Gibb JM, Majeed JA, Al Romaihi HE. Effectiveness of Ehteraz digital contact tracing app versus conventional contact tracing in managing the outbreak of COVID-19 in the State of Qatar. *BMJ Innovations* 2022;**8**:255 - 60. https://doi.org/https://dx.doi.org/10.1136/bmjinnov-2021-000879 | Outcome |
| 2. | Borrell S, Thorne N, Espanol M, Mortimer C, Orcau A, Coll P*, et al.* Comparison of four-colour IS<ovid:i>6110 -fAFLP with the classic IS<ovid:i>6110 -RFLP on the ability to detect recent transmission in the city of Barcelona, Spain. *Tuberculosis* 2009;**89**:233-7. https://doi.org/https://dx.doi.org/10.1016/j.tube.2009.03.003 | Outcome |
| 3. | Brewer DD, Potterat JJ, Muth SQ, Malone PZ, Montoya P, Green DL*, et al.* Randomized trial of supplementary interviewing techniques to enhance recall of sexual partners in contact interviews. *Sex Transm Dis* 2005;**32**:189-93. https://doi.org/https://dx.doi.org/10.1097/01.olq.0000154492.98350.90 | Outcome |
| 4. | Carre H, Boman J, Osterlund A, Garden B, Nylander E. Improved contact tracing for <ovid:i>Chlamydia trachomatis with experienced tracers, tracing for one year back in time and interviewing by phone in remote areas. *Sex Transm Infect* 2008;**84**:239-42. https://doi.org/https://dx.doi.org/10.1136/sti.2007.028068 | Outcome |
| 5. | Cassell JA, Dodds J, Estcourt C, Llewellyn C, Lanza S, Richens J*, et al.* The relative clinical effectiveness and cost-effectiveness of three contrasting approaches to partner notification for curable sexually transmitted infections: a cluster randomised trial in primary care. *Health technol assess* 2015;**19**:1-viii. https://doi.org/https://dx.doi.org/10.3310/hta19050 | Outcome |
| 6. | Chow BWK, Lim YD, Poh RCH, Ko A, Hong GH, Zou SWL*, et al.* Use of a digital contact tracing system in Singapore to mitigate COVID-19 spread. *BMC Public Health* 2023;**23**:2253. https://doi.org/https://dx.doi.org/10.1186/s12889-023-17150-0 | Outcome |
| 7. | Danquah LO, Hasham N, MacFarlane M, Conteh FE, Momoh F, Tedesco AA*, et al.* Use of a mobile application for Ebola contact tracing and monitoring in northern Sierra Leone: a proof-of-concept study. *BMC Infect Dis* 2019;**19**:810. https://doi.org/https://dx.doi.org/10.1186/s12879-019-4354-z | Outcome |
| 8. | Ehlman DC, Jackson M, Saenz G, Novak DS, Kachur R, Heath JT*, et al.* Evaluation of an innovative internet-based partner notification program for early syphilis case management, Washington, DC, January 2007-June 2008. *Sex Transm Dis* 2010;**37**:478-85. https://doi.org/https://dx.doi.org/10.1097/OLQ.0b013e3181e212cb | Outcome |
| 9. | Falk L, Hegic S, Wilson D, Wirehn AB. Home-sampling as a tool in the context of <ovid:i>Chlamydia trachomatis partner notification: a randomized controlled trial. *Acta Derm Venereol* 2014;**94**:72-4. https://doi.org/https://dx.doi.org/10.2340/00015555-1624 | Outcome |
| 10. | Handmann E, Camanor SW, Fallah MP, Candy N, Parker D, Gries A*, et al.* Feasibility of digital contact tracing in low-income settings - pilot trial for a location-based DCT app. *BMC Public Health* 2023;**23**:146. https://doi.org/https://dx.doi.org/10.1186/s12889-022-14888-x | Outcome |
| 11. | Johnson K, Diallo K, Hennein R, Shelby T, Zhou X, Gupta AJ*, et al.* Centering Health Equity Within COVID-19 Contact Tracing: Connecticut's Community Outreach Specialist Program. *Journal of public health management and practice : JPHMP* 2022;**28**:728-38. https://doi.org/https://dx.doi.org/10.1097/PHH.0000000000001608 | Outcome |
| 12. | Kalichman S, Banas E, Kalichman M, Dewing S, Jennings K, Daniels J*, et al.* Brief Enhanced Partner Notification and Risk Reduction Counseling to Prevent Sexually Transmitted Infections, Cape Town, South Africa. *Sex Transm Dis* 2021;**48**:174-82. https://doi.org/https://dx.doi.org/10.1097/OLQ.0000000000001295 | Outcome |
| 13. | Maierhofer CN, Powers KA, Matoga MM, Chen JS, Jere E, Massa C*, et al.* Characterizing Network-Based HIV Testing Interventions to Guide HIV Testing and Contact Tracing at STI Clinics in Lilongwe, Malawi. *Journal of acquired immune deficiency syndromes (1999)* 2023;**94**:151-9. https://doi.org/https://dx.doi.org/10.1097/QAI.0000000000003240 | Outcome |
| 14. | Ramstedt K, Forssman L, Johannisson G. Contact tracing in the control of genital Chlamydia trachomatis infection. *Int J STD AIDS* 1991;**2**:116-8. https://doi.org/10.1177/095646249100200208 | Outcome |
| 15. | Rane V, Tomnay J, Fairley C, Read T, Bradshaw C, Carter T*, et al.* Opt-Out Referral of Men Who Have Sex With Men Newly Diagnosed With HIV to Partner Notification Officers: Results and Yield of Sexual Partners Being Contacted. *Sex Transm Dis* 2016;**43**:341-5. https://doi.org/https://dx.doi.org/10.1097/OLQ.0000000000000449 | Outcome |
| 16. | Raymond C, Ouyang D, D'Agostino A, Rudman SL, Ho DE. Automated vs. manual case investigation and contact tracing for pandemic surveillance: Evidence from a stepped wedge cluster randomized trial. *EClinicalMedicine* 2023;**55**:101726. https://doi.org/https://dx.doi.org/10.1016/j.eclinm.2022.101726 | Outcome |
| 17. | Rose SB, Garrett SM, Hutchings D, Lund K, Kennedy J, Pullon SRH. Clinician education, advice and SMS/text reminders improve test of reinfection rates following diagnosis of Chlamydia trachomatis or Neisseria gonorrhoeae : before and after study in primary care. *BMJ sex reprod health* 2019; https://dx.doi.org/10.1136/bmjsrh-2018-200185. https://doi.org/https://dx.doi.org/10.1136/bmjsrh-2018-200185 | Outcome |
| 18. | Senior SL. Self-completed online contact tracing for COVID-19 is associated with reporting fewer contacts: an observational study. *J public health (Oxf)* 2023;**45**:e409-e16. https://doi.org/https://dx.doi.org/10.1093/pubmed/fdac164 | Outcome |
| 19. | Shah L, Rojas M, Mori O, Zamudio C, Kaufman JS, Otero L*, et al.* Implementation of a stepped-wedge cluster randomized design in routine public health practice: design and application for a tuberculosis (TB) household contact study in a high burden area of Lima, Peru. *BMC Public Health* 2015;**15**:587. https://doi.org/https://dx.doi.org/10.1186/s12889-015-1883-2 | Outcome |
| 20. | Tomnay JE, Pitts MK, Kuo TC, Fairley CK. Does the Internet assist clients to carry out contact tracing? A randomized controlled trial using web-based information. *Int J STD AIDS* 2006;**17**:391-4. https://doi.org/https://dx.doi.org/10.1258/095646206777323391 | Outcome |
| 21. | Toomey KE, Peterman TA, Dicker LW, Zaidi AA, Wroten JE, Carolina J. Human immunodeficiency virus partner notification. Cost and effectiveness data from an attempted randomized controlled trial. *Sex Transm Dis* 1998;**25**:310-6. https://doi.org/https://dx.doi.org/10.1097/00007435-199807000-00008 | Outcome |
| 22. | Wynendaele B, Bomba W, M'Manga W, Bhart S, Fransen L. Impact of counselling on safer sex and STD occurrence among STD patients in Malawi. *Int J STD AIDS* 1995;**6**:105-9. https://doi.org/https://dx.doi.org/10.1177/095646249500600208 | Outcome |
| 23. | Zhang T, Guo L, Zhang S, Liu W, Chen G, Hui M*, et al.* Improving detection and notification of tuberculosis cases in students in Shaanxi province, China: an intervention study. *BMC Public Health* 2011;**11**:147. https://doi.org/https://dx.doi.org/10.1186/1471-2458-11-147 | Outcome |
|  |  |  |
| 1. | Bowdoin CD, Buchanan CS. Contact investigation in unorganized Georgia counties. *The Journal of venereal disease information* 1948;**29**:340-2. | Publication status |
| 2. | Chiou PY, Wang CC, Chuang P, Yen MY, Chang CL. The effect of advanced partner notification for people living with HIV and AIDS. *Sex Transm Infect* 2013;**89**. https://doi.org/10.1136/sextrans-2013-051184.1048 | Publication status |
| 3. | Clark J, Oldenburg C, Segura E, Rios J, Villaran M, Salvatierra J*, et al.* New and traditional notification tools improve partner notification outcomes among MSM with syphilis infection in Lima, Peru. *Sex Transm Infect* 2015;**91**:A39‐A40. https://doi.org/10.1136/sextrans-2015-052270.115 | Publication status |
| 4. | Davis JL, Turimumahoro P, Gupta AJ, Ochom E, Ggita JM, Nakasendwa S*, et al.* A User-centered Strategy to Improve the Implementation and Effectiveness of Tuberculosis Contact Investigation in Uganda: a Stepped-wedge, Cluster-randomized Trial. *Am J Respir Crit Care Med* 2024;**209**. | Publication status |
| 5. | Easley EJ, Parkhurst GE, Swank RR. The 100-day experiment in contact investigation in Arkansas. *The Journal of venereal disease information* 1948;**29**:13-9. | Publication status |
| 6. | Martinson NA, Lebina L, Webb E, Ratsela A, Golub J, Bosch Z*, et al.* A cluster randomized trial of contact tracing in households of index patients with TB. *Top Antivir Med* 2021;**29**:37. | Publication status |
| 7. | Mathews C, Kalichman M, Dewing S, Banas E, Dumile S, Mdlikiva A*, et al.* Effects of enhanced STI partner notification counseling in Cape Town: randomized controlled trial. *Sex Transm Infect* 2019;**95**:A66. https://doi.org/10.1136/sextrans-2019-sti.173 | Publication status |
| 8. | Rion JW, Iskrant AP. Differentials in the process of contact investigation. *The Journal of venereal disease information* 1948;**29**:231-9. | Publication status |
| 9. | Sachdev DD, Brosnan HK, Reid MJA, Kirian M, Cohen SE, Nguyen TQ*, et al.* Outcomes of Contact Tracing in San Francisco, California-Test and Trace During Shelter-in-Place. *JAMA internal medicine* 2021;**181**:381-3. https://doi.org/https://dx.doi.org/10.1001/jamainternmed.2020.5670 | Publication status |
| 10. | Salazar-Austin N, Cohn S, Barnes G, Tladi M, Matlhaoleng K, Martinson N*, et al.* IPT coverage in nurse-led screening of child tuberculosis contacts. *Top Antivir Med* 2017;**25**:304s. | Publication status |
| 11. | Spoto JS, Iskrant AP. Contact investigation of syphilis. *The Journal of venereal disease information* 1949;**30**:140-4. | Publication status |
| 12. | van Geuns HA, Meijer J, Styblo K. Results of contact examination in Rotterdam, 1967-1969. *Bulletin of the International Union against Tuberculosis* 1975;**50**:107-21. | Publication status |
|  |  |  |
| 1. | Dasgupta K, Schwartzman K, Marchand R, Tennenbaum TN, Brassard P, Menzies D. Comparison of cost-effectiveness of tuberculosis screening of close contacts and foreign-born populations. *Am J Respir Crit Care Med* 2000;**162**:2079-86. https://doi.org/https://dx.doi.org/10.1164/ajrccm.162.6.2001111 | Setting |
| 2. | Huang Z, Guo H, Lee Y-M, Ho EC, Ang H, Chow A. Performance of Digital Contact Tracing Tools for COVID-19 Response in Singapore: Cross-Sectional Study. *JMIR mHealth and uHealth* 2020;**8**:e23148. https://doi.org/https://dx.doi.org/10.2196/23148 | Setting |
|  |  |  |
| 1. | Akhmetzhanov AR, Cheng H-Y, Linton NM, Ponce L, Jian S-W, Lin H-H. Transmission Dynamics and Effectiveness of Control Measures during COVID-19 Surge, Taiwan, April-August 2021. *Emerg Infect Dis* 2022;**28**:2051-9. https://doi.org/https://dx.doi.org/10.3201/eid2810.220456 | Study design |
| 2. | Ami J, Ishii K, Sekimoto Y, Masui H, Ohmukai I, Yamamoto Y*, et al.* Computation of Infection Risk via Confidential Locational Entries: A Precedent Approach for Contact Tracing with Privacy Protection. *IEEE Access* 2021;**9**:87420-33. https://doi.org/10.1109/ACCESS.2021.3087478 | Study design |
| 3. | Anonymous. THE SOCIAL BACKGROUND OF VENEREAL DISEASE: A REPORT ON AN EXPERIMENT IN CONTACT TRACING AND AN INVESTIGATION INTO SOCIAL CONDITIONS: TYNESIDE EXPERIMENTAL SCHEME IN VENEREAL DISEASE CONTROL OCTOBER 1943 TO MARCH 1944. *The British journal of venereal diseases* 1945;**21**:26-34. | Study design |
| 4. | Baluku JB, Kabamooli RA, Kajumba N, Nabwana M, Kateete D, Kiguli S*, et al.* Contact tracing is associated with treatment success of index tuberculosis cases in Uganda. *Int J Infect Dis* 2021;**109**:129-36. https://doi.org/https://dx.doi.org/10.1016/j.ijid.2021.06.049 | Study design |
| 5. | Banks GS. The Value of the Examination of Contacts in Pulmonary Tuberculosis. *Edinb Med J* 1937;**44**:153-64. | Study design |
| 6. | Bell G, Ward H, Day S, Ghani AC, Goan U, Claydon E*, et al.* Partner notification for gonorrhoea: a comparative study with a provincial and a metropolitan UK clinic. *Sex Transm Infect* 1998;**74**:409-14. https://doi.org/https://dx.doi.org/10.1136/sti.74.6.409 | Study design |
| 7. | Chambers T, Anglemyer A, Chen A, Atkinson J, Elers P, Baker MG. An evaluation of the population uptake and contact tracer utilisation of the Covid-19 Bluetooth Exposure Notification Framework in New Zealand. *Aust N Z J Public Health* 2024;**48**:100197. https://doi.org/https://dx.doi.org/10.1016/j.anzjph.2024.100197 | Study design |
| 8. | Dongo JP, Graham SM, Nsonga J, Wabwire-Mangen F, Maleche-Obimbo E, Mupere E*, et al.* Implementation of an Effective Decentralised Programme for Detection, Treatment and Prevention of Tuberculosis in Children. *Tropical medicine and infectious disease* 2021;**6**. https://doi.org/https://dx.doi.org/10.3390/tropicalmed6030131 | Study design |
| 9. | Feldman KA, Hanks A, Williams TW, Blouse B, Babcock C, Goode A*, et al.* A State Health Department and Health Information Exchange Partnership: An Effective Collaboration for a Data-Driven Response for COVID-19 Contact Tracing in Maryland. *Sex Transm Dis* 2023;**50**:S34-S40. https://doi.org/https://dx.doi.org/10.1097/OLQ.0000000000001702 | Study design |
| 10. | Folke T, Menon-Johansson AS. An Evaluation of Digital Partner Notification Tool Engagement and Impact for Patients Diagnosed With Gonorrhea and Syphilis. *Sex Transm Dis* 2022;**49**:815-21. https://doi.org/https://dx.doi.org/10.1097/OLQ.0000000000001707 | Study design |
| 11. | Free C, McCarthy O, French RS, Wellings K, Michie S, Roberts I*, et al.* Can text messages increase safer sex behaviours in young people? Intervention development and pilot randomised controlled trial. *Health technol assess* 2016;**20**:1-82. https://doi.org/https://dx.doi.org/10.3310/hta20570 | Study design |
| 12. | Golden MR, Hughes JP, Brewer DD, Holmes KK, Whittington WLH, Hogben M*, et al.* Evaluation of a population-based program of expedited partner therapy for gonorrhea and chlamydial infection. *Sex Transm Dis* 2007;**34**:598-603. https://doi.org/https://dx.doi.org/10.1097/01.olq.0000258319.54316.06 | Study design |
| 13. | Hammar H, Ljungberg L. Factors affecting contact tracing of gonorrhoea. *Acta Derm Venereol* 1972;**52**:233-40. | Study design |
| 14. | Ilesanmi OS, Afolabi AA. COVID-19 waves in Africa: Effects of outbreak response and interventions. *Global Biosecurity* 2021;**3**. https://doi.org/10.31646/gbio.104 | Study design |
| 15. | Judson FN. Partner notification for HIV control. *Hosp Pract* 1990;**25**:63-73. | Study design |
| 16. | Kakinda M, Matovu JKB. A yield and cost comparison of tuberculosis contact investigation and intensified case finding in Uganda. *PLoS One* 2020;**15**:e0234418. https://doi.org/https://dx.doi.org/10.1371/journal.pone.0234418 | Study design |
| 17. | Khaparde K, Jethani P, Dewan PK, Nair SA, Deshpande MR, Satyanarayana S*, et al.* Evaluation of TB Case Finding through Systematic Contact Investigation, Chhattisgarh, India. *Tuberculosis research and treatment* 2015;**2015**:670167. https://doi.org/https://dx.doi.org/10.1155/2015/670167 | Study design |
| 18. | Kim DY, Ridzon R, Giles B, Mireles T, Garrity K, Hathcock AL*, et al.* A no-name tuberculosis tracking system. *Am J Public Health* 2003;**93**:1637-9 | Study design |
| 19. | Kohl KS, Farley TA, Ewell J, Scioneaux J. Usefulness of partner notification for syphilis control. *Sex Transm Dis* 1999;**26**:201-7. https://doi.org/https://dx.doi.org/10.1097/00007435-199904000-00003 | Study design |
| 20. | Lambregts-van Weezenbeek CSB, Sebek MMGG, van Gerven PJHJ, de Vries G, Verver S, Kalisvaart NA*, et al.* Tuberculosis contact investigation and DNA fingerprint surveillance in The Netherlands: 6 years' experience with nation-wide cluster feedback and cluster monitoring*. Int J Tuberc Lung Dis* 2003;**7**:S463-70. | Study design |
| 21. | Leung KK, Zhang R, Hashim MJ, Fang M, Xu J, Sun D*, et al.* Effectiveness of containment strategies in preventing SARS-CoV-2 transmission. *Journal of infection and public health* 2022;**15**:609-14. https://doi.org/https://dx.doi.org/10.1016/j.jiph.2022.04.012 | Study design |
| 22. | Li Z-J, Tu W-X, Wang X-C, Shi G-Q, Yin Z-D, Su H-J*, et al.* A practical community-based response strategy to interrupt Ebola transmission in Sierra Leone, 2014-2015. *Infectious diseases of poverty* 2016;**5**:74. https://doi.org/https://dx.doi.org/10.1186/s40249-016-0167-0 | Study design |
| 23. | Liliia M, Geliukh E, Islam Z, Davtyan H. Optimized Case Finding of tuberculosis among key populations in Ukraine. A follow up study. *Journal of infection in developing countries* 2024;**18**:53-9. https://doi.org/https://dx.doi.org/10.3855/jidc.17532 | Study design |
| 24. | Lu L, Anderson B, Ha R, D'Agostino A, Rudman SL, Ouyang D*, et al.* A language-matching model to improve equity and efficiency of COVID-19 contact tracing. *Proc Natl Acad Sci U S A* 2021;**118**. https://doi.org/https://dx.doi.org/10.1073/pnas.2109443118 | Study design |
| 25. | Mandalakas AM, Ngo K, Alonso Ustero P, Golin R, Anabwani F, Mzileni B*, et al.* BUTIMBA: Intensifying the Hunt for Child TB in Swaziland through Household Contact Tracing. *PLoS One* 2017;**12**:e0169769. https://doi.org/https://dx.doi.org/10.1371/journal.pone.0169769 | Study design |
| 26. | Masiuk L, Denisiuk O, Geliukh E, Aslanyan G, Zachariah R, Islam Z. Breaking the paradigm: Optimized Case Finding multiplies tuberculosis detection among key populations in Ukraine. *Journal of infection in developing countries* 2021;**15**:75S-81S. https://doi.org/https://dx.doi.org/10.3855/jidc.13806 | Study design |
| 27. | Mickiewicz T, Al-Tayyib A, Thrun M, Rietmeijer C. Implementation and effectiveness of an expedited partner therapy program in an urban clinic. *Sex Transm Dis* 2012;**39**:923-9. https://doi.org/https://dx.doi.org/10.1097/OLQ.0b013e3182756f20 | Study design |
| 28. | Namukose E, Bowah C, Cole I, Dahn G, Nyanzee P, Saye R*, et al.* Active Case Finding for Improved Ebola Virus Disease Case Detection in Nimba County, Liberia, 2014/2015: Lessons Learned. *Advances in Public Health* 2018; 10.1155/2018/6753519:1-7. https://doi.org/10.1155/2018/6753519 | Study design |
| 29. | Oladimeji AM, Afe AJ, Carillo L, Hundley C, Yufang Z, Long S*, et al.* Epidemiology and control of monkeypox outbreak in Houston, Texas. *Global Health Journal* 2024;**8**:133-9. https://doi.org/10.1016/j.glohj.2024.08.008 | Study design |
| 30. | Osterlund A. Improved partner notification for genital chlamydia can be achieved by centralisation of the duty to a specially trained team. *Int J STD AIDS* 2014;**25**:1009-12. https://doi.org/https://dx.doi.org/10.1177/0956462414527265 | Study design |
| 31. | Pilny A, Huber CJ. An Egocentric Network Contact Tracing Experiment: Testing Different Procedures to Elicit Contacts and Places. *Int J Environ Res Public Health* 2021;**18**. https://doi.org/https://dx.doi.org/10.3390/ijerph18041466 | Study design |
| 32. | Richard UE, Aneaton E, Samuel I, Ifeanyi O. Partner Notification Services (PNS) Among HIV Positive Key Populations: An Important Strategy Towards Achieving UNAIDS 95.95.95 Goals by the Year 2030. *Texila International Journal of Public Health* 2021;**9**. https://doi.org/10.21522/TIJPH.2013.09.03.Art016 | Study design |
| 33. | Rodriguez P, Grana S, Alvarez-Leon EE, Battaglini M, Darias FJ, Hernan MA*, et al.* A population-based controlled experiment assessing the epidemiological impact of digital contact tracing. *Nature communications* 2021;**12**:587. https://doi.org/https://dx.doi.org/10.1038/s41467-020-20817-6 | Study design |
| 34. | Saunders MJ, Tovar MA, Collier D, Baldwin MR, Montoya R, Valencia TR*, et al.* Active and passive case-finding in tuberculosis-affected households in Peru: a 10-year prospective cohort study. *The Lancet Infectious diseases* 2019;**19**:519-28. https://doi.org/https://dx.doi.org/10.1016/S1473-3099(18)30753-9 | Study design |
| 35. | Sylvan SPE, Hedlund J. Efficacy of partner notification for Chlamydia trachomatis among young adults in youth health centres in Uppsala County, Sweden. *Journal of the European Academy of Dermatology and Venereology : JEADV* 2009;**23**:517-22. https://doi.org/https://dx.doi.org/10.1111/j.1468-3083.2008.03080.x | Study design |
| 36. | van Loenhout-Rooyacke JH, Sebek MMGG, Verbeek ALM. Contact tracing using DNA fingerprinting in an asylum seeker with pulmonary tuberculosis. *The Netherlands journal of medicine* 2002;**60**:281-4. | Study design |
| 37. | Vest JR, Valadez AM, Hanner A, Lee JH, Harris PB. Using e-mail to notify pseudonymous e-mail sexual partners. *Sex Transm Dis* 2007;**34**:840-5. https://doi.org/https://dx.doi.org/10.1097/OLQ.0b013e318073bd5d | Study design |
| 38. | Wang X, Shi L, Zhang Y, Chen H, Jiao J, Yang M*, et al.* A Comparative Retrospective Study of COVID-19 Responses in Four Representative Asian Countries. *Risk Manag Healthc Policy* 2022;**15**:13-25. https://doi.org/https://dx.doi.org/10.2147/RMHP.S334326 | Study design |
| 39. | Wanyana MW, Akunzirwe R, King P, Atuhaire I, Zavuga R, Lubwama B*, et al.* Performance and impact of contact tracing in the Sudan virus outbreak in Uganda, September 2022-January 2023. *Int J Infect Dis* 2024;**141**:106959. https://doi.org/https://dx.doi.org/10.1016/j.ijid.2024.02.002 | Study design |
| 40. | Willcox RR. International contact tracing in venereal disease. *WHO Chron* 1973;**27**:418-22. | Study design |
| 41. | Wymant C, Ferretti L, Tsallis D, Charalambides M, Abeler-Dorner L, Bonsall D*, et al.* The epidemiological impact of the NHS COVID-19 app. *Nature* 2021;**594**:408-12. https://doi.org/https://dx.doi.org/10.1038/s41586-021-03606-z | Study design |
|  |  |  |
| 1. | Asuzu MC, Rotowa NA, Ajayi IO. The use of mail reminders in STD contact tracing in Ibadan, Nigeria. *East Afr Med J* 1990;**67**:75-8. | Not found |
| 2. | Azar JE. Source detection and contact investigation in tuberculosis control. *Doc Med Geogr Trop* 1957;**9**:45-51. | Not found |
| 3. | Brugueras S, Orcau A, Millet JP, Espinosa L, de Andres A, Gorrindo P*, et al.* Tuberculosis clinical units improve contact tracing. *Int J Tuberc Lung Dis* 2016;**20**:1572-9. https://doi.org/https://dx.doi.org/10.5588/ijtld.16.0147 | Not found |
| 4. | Dougherty WJ. Venereal disease contact investigation, Navy and Marine Corps, April-June 1948. *U S Nav Med Bull* 1949;**49**:957-66. | Not found |
| 5. | Gray AL, Iskrant AP, Hibbets RS. Syphilis contact investigation in a rural county in Mississippi. *The Journal of venereal disease information* 1949;**30**:165-9. | Not found |
| 6. | Hoffman RE, Spencer NE, Miller LA. Comparison of partner notification at anonymous and confidential HIV test sites in Colorado. *J Acquir Immune Defic Syndr Hum Retrovirol* 1995;**8**:406-10. | Not found |
| 7. | Jensen SG, Lillebaek T, Wilcke T, Pedersen MK, Andersen PHS, Olsen NW*, et al.* Impact of contact investigation and tuberculosis screening among high-risk groups in Denmark. *Int J Tuberc Lung Dis*2016;**20**:1580-7. https://doi.org/https://dx.doi.org/10.5588/ijtld.16.0318 | Not found |
| 8. | Kassler WJ, Meriwether RA, Klimko TB, Peterman TA, Zaidi A. Eliminating access to anonymous HIV antibody testing in North Carolina: effects on HIV testing and partner notification. *J Acquir Immune Defic Syndr Hum Retrovirol* 1997;**14**:281-9. https://doi.org/https://dx.doi.org/10.1097/00042560-199703010-00013 | Not found |
| 9. | Pennini SN, Pedrosa L, Rebello PFB. Early diagnosis and treatment of leprosy in intradomiciliary contacts in a high prevalence area: Amazon region. *Indian J Lepr* 1998;**70**:73S-7S. | Not found |
| 10. | Pointe ML, Bailey WC, Greenberg HB. Contact investigation: an important aspect of TB control. *The Journal of the Louisiana State Medical Society* 1973;**125**:197-200. | Not found |
| 11. | Rathore AS, Misra RS, Ramesh V. Domiciliary health counselling in patients with sexually transmitted diseases. *The Journal of communicable diseases* 1997;**29**:35-9. | Not found |
| 12. | Reynolds VJ. Epidemiology and contact tracing. *Update* 1978;**17**:327 - 33. | Not found |
| 13. | Zamanpour A, Grennan T, Ablona A, Fairley CK, Estcourt C, Mema S*, et al.* Treatment and Partner Notification Outcomes for Clients Diagnosed With Chlamydia and Gonorrhea Through GetCheckedOnline Compared With Sexually Transmitted Infection Clinics in British Columbia, Canada. *Sex Transm Dis* 2023;**50**:86-91. https://doi.org/https://dx.doi.org/10.1097/OLQ.0000000000001735 | Not found |
