# Supplementary material for: The Effectiveness of Contact Tracing to Reduce Transmission of Infectious Diseases During Epidemic or Pandemic Response: Rapid Systematic Review
Source: JMIR Public Health Surveill. 2026 Mar 31;12:e84805. doi: 10.2196/84805 (PMC13080299; doi:10.2196/84805)
Supplement: Multimedia Appendix 6 [file publichealth_v12i1e84805_app6.docx]

Table S1. Sample characteristics of prioritised studies

| **Author, date; country** | **Study design** | **Infection study is focused on** | **Study start and end dates (duration, years (y) /months (m))** | **No. invited to participate / Total no. of participants (all arms)** | **Study setting / Recruitment setting and criteria** | **For whole sample index cases** | | | |
| --- | --- | --- | --- | --- | --- | --- | --- | --- | --- |
|  |  |  |  |  |  | **Age (years)**  **Median (IQR)** | **Socioeconomic status (including education, occupation, and income)** | **Ethnicity** | **(% female)** |
| Andersen, 1998;^1^ Denmark | RCT | Chlamydia | NR | NR / 96 | Community / Clinic attendees: women with chlamydia seen in general practices in Aarhus County, Denmark. | NR | NR | NR | 100 |
| Apoola, 2009;^2^ UK | RCT | Chlamydia | NR | 207 / 200 | STI clinic / Clinic attendees: female patients with a diagnosis of genital chlamydia infection. | Swab testing: 20 (18-23), urine testing: 22 (19-25) | Highest educational attainment (secondary school): 70% | White: 90% | 100 |
| Bai, 2008;^3^ China | OCC | TB | October 2003-September 2004 (11m) | 14130 / NA | Community / Health records were searched for participants. Criteria were not reported. | NR | NR | NR | NR |
| Brown, 2011;^4^ Malawi | RCT | HIV | October 2008-September 2009 (11m) | 267 / 240 | Hospital based STI clinic / Adult clinic attendees from Lilongwe with a first positive HIV test and had been sexually active in the last 90 days. | 28 (24-33) | Education: median years (IQR): whole sample 8 (5-11) | NR | 58.3 |
| Cavalcante, 2010;^5^ Brazil | cRCT | TB | November 2000-December 2004, (4y1m) | 2215  / 712 | Community / Health centre attendees who were newly diagnosed with and treated for pulmonary TB living in selected neighbourhoods. | Mean: 36 | Unemployment: 276 (39%). Education: None 55 (8%), <8 years 395 (55%), ≥8 years 262 (37%). Monthly family wage: <1 minimum wage 114 (16%), 1 to <2 minimum wage 217 (30%), 2 to <4 minimum wage 197 (28%). | White: 38.5%, Non-White: 61.5% | 32.6 |
| Chen, 2021;^6^ Malawi | RCT | HIV | June 2015-May 2019 (3y11m) | 19288 / 1885 | Hospital-based STI clinic / STI clinic attendees ≥18 years old, living in Lilongwe District, who reported having sex in the previous six months. | 18-24: 25.5%,  25-34: 46.4%,  35-44: 22.5%, ≥45: 4.5% | NR | NR | 60.50 |
| Cherutich, 2017;^7^ Kenya | cRCT | HIV | August 2013-August 2015 (2y) | 1760 / 1119 | Public HIV testing clinics and faith-based health facilities / Clinic attendees who were: ≥18 years old; not pregnant; willing to provide consent and sex partner information; newly or recently diagnosed with HIV; not yet linked to HIV care or linked to care within the preceding 6 weeks; and had reported no IPV in the preceding month. Excluded: attendees who lived outside a 50 km radius from the study site. | Arm 1: 30 (25-37), arm 2: 21 (26-38) | Unemployed 23% (student 1.5%, economic support 5.5%, other 16%), employed 77% | NR | 61.6 |
| Chiou, 2015;^8^ Taiwan | RCT | HIV | March 2012-March 2013 (1y) | 118 / 85 | Hospital infectious disease outpatient department / Clinic attendees: HIV positive MSM infected through unsafe sex who were literate and able to communicate. | Mean (SD): 28.06 (4.33) | Education: high school 28.2%, college/university 60.7%, grand school 13.1%. Employed: 79.8%. Monthly income (USD): none 6%, <667 23.8%, 667-1332 56.0%, 1,333–1,666 6.0%, >1667 8.3%. | NR | 0.0 |
| Choko, 2021;^9^ Malawi | cRCT | HIV | 8^th^ Sept 2018-2^nd^ May 2019 (8m) | 5252 / 5106 | Community / ANC arm: women attending their first ANC visit who were ≥18 years old and intending to remain in clinic catchment area, and whose male partner (child’s probable father) was present and reachable, not already receiving ART, or not already tested during this pregnancy. Excluded: women whose male partner was <18 years or not resident in the catchment area. Index arm: all clinic patients newly diagnosed with HIV, aged at least 18 years, intending to remain in the clinic catchment area, not already enrolled, who had at least one contactable sexual partner not already known to be HIV-positive. Excluded: patients with all sexual contacts aged <18 years, already receiving ART, or not available and resident in the catchment area. | ANC: Standard 22 (19-28), HIVST only 22 (19-27), HIVST incentive 23 (19-33).  Index: Standard 26 (22-32), HIVST only 26 (22-33), HIVST incentive 27 (23-33). | ANC: able to read and write: yes 89.5%, no 9.8%, data missing 0.7%. Index: able to read and write: yes 82.2%, no 15.3%, data missing 2.5%. | NR | ANC: 100,  index: 73.5 |
| Clark, 2018;^10^ Peru | RCT (pilot) | Syphilis | November 2012-July 2014 (1y8m) | 537 / 370 | Clinic and community / MSM and TW were recruited from the community and HIV/STI clinics who were diagnosed with untreated syphilis (primary, secondary, or latent) and reported anal intercourse with a male or transgender female partner in the previous 6 months. Excluded: people with a previously treated infection. | Arm 1: 27 (23-34), arm 2: 27 (23-35), arm 3: 26 (23-30), control: 30 (24-35). | Primary school only: arm 1: 2.1%, arm 2: 0%, arm 3: 0%, control: 2.2%; incomplete secondary school: arm 1: 9.5%, arm 2: 10.8%, arm 3: 11.3%, control: 12.1%; complete secondary school: arm 1: 21.0%, arm 2: 27.4%, arm 3: 25.8%, control: 25.5%; university or vocational training: arm 1: 67.4%, arm 2: 61.9%, arm 3: 62.9%, control: 60.1%. | NR | NR |
| Culbert, 2023;^11^ Indonesia | RCT | HIV | January 2020-January 2021 (1y) | 66 / 55 | Prison / Incarcerated men attending regular clinic checkups who were: ≥18 years old; HIV positive; self-reported to be sexually active and/or injecting drugs in the year before incarceration; incarcerated within the last 3 years; and > 6 months from their release date. | 36 (32-40) | NR | NR | 0.0 |
| Dibia, 2024;^12^ Nigeria | OCC | HIV | March 2021-April 2022 (1y1m) | 2705 / 2482 | Health facility / Facilities with a high client load of at least 2,000 active clients on ART and a high HIV-testing volume. Clinic attendees who were newly diagnosed with HIV and visited the facility for ART refill. | 15-24 years old: 7.5%, 25-49 years old: 82.0%, ≥50 years old: 10.5%. | NR | NR | 65.4 |
| England, 2005;^13^ Australia | OCC | Chlamydia | 1^st^ September 2002-30^th^ September 2003 (1y1m) | 531 / 391 | Clinic / The ACT Health Department patient register was searched for all patients with genital chlamydia infection notified during the study period. | NR | NR | NR | NR |
| Estcourt, 2012;^14^ UK | nrCT | STIs (chlamydia, gonorrhoea, NGU) | September 2011-July 2013 (1y10m) | 266 / 199 | STI clinic / Female clinic attendees ≥16 years old with genital chlamydia infection and at least one untreated contactable male sex partner in the last 6 months. Excluded: attendees with known HIV positive status, co-infection with other STIs, or who were unable to understand English. Male sex partners were excluded if they had symptoms of complicated infection, an allergy or contraindications to azithromycin, or were unable to understand English. | NR | NR | NR | NR |
| Estcourt, 2015^15^/2016;^16^ UK (England) | RCT (pilot) | Chlamydia | July 2018-February 2020 (including 3-month follow-up period after end of intervention phases in November 2019) (1y7m) | 16 445 / 1724 | Primary care / Heterosexual clinic attendees ≥16 years old with positive chlamydia test or clinical diagnosis of conditions for which presumptive chlamydia treatment and CT are initially provided, with a contactable sexual partner in the past 6 months. Exclusion: people with test results subsequently negative for chlamydia, MSM, people with complex circumstances, people who paid for or been paid for sex in the past 6 months, or those with insufficient English language skills. | Mean (SD) [range]: control: 25.6 (6.4) [17-62], intervention: 25.7 (7.0) [16-72]. | NR | White British or Irish: 47%, White other: 12%, Black or Black British: 23%, Asian or British Asian: 6%, Mixed: 10%, Other: 2%. | Control: 68.0 Intervention: 66.0 |
| Estcourt, 2022^17^/2024;^18^ UK (England, Scotland) | cRCT | Chlamydia | NR | 451 / 226 | Sexual health clinic / Index cases included: clinic attendees ≥16 years old with a laboratory diagnosis of chlamydia and/or gonorrhoea (men and women) or NGU (men only), and a contactable sex partner. Excluded if unable to understand English or coexistent infection with other STIs. Partners were excluded if aged <16 years or pregnant, if they had symptoms of complicated infection or contraindications to azithromycin and/or cefixime, or were unable to understand English. | NR | NR | NR | NR |
| Faxelid, 1996;^19^ Zambia | RCT | STIs (syphilis, chancroid, lymphogranuloma venereum, gonorrhoea, trichomoniasis) | October 1992-March 1993 (5m) | NR / 396 | Urban health centre / Clinic attendees with an STD, who had received information and routine STD care. Excluded if they had more than one diagnosis. | Mean (range): 28 (15-51) | Years in school: mean 8.9 | NR | 24.0 |
| Fetzer, 2021;^20^ England | cITS | Covid-19 | Pre-treatment period: 6^th^-20^th^ September; treatment period (Excel error): 20^th-^27^th^ September; post-treatment period: 27^th^ September-25^th^ October (1.5m) | NR / NR | Nationwide program / Public databases were searched for cases. Criteria were not reported. | NR | NR | NR | NR |
| Findlater, 2023;^21^ England | cITS) | Covid-19 | 30^th^ September-5^th^ October 2020 (6d) | 15 285 / 59 027 | Community / Primary cases in the control group were sourced from health records and restricted to those arising from community testing. | < 18: 8.5%,  18-29: 45%,  30-49: 25.5%,  50-69: 17.5%,  ≥70: 3.5% | NR | Black: 2%, Indian: 3%,  Mixed: 2%,  Other: 1%,  Other Asian: 3%,  Pakistani: 4.5%,  White: 58%,  Not stated: 27% | 52.5 |
| Hanrahan, 2019;^22^ South Africa | Parallel cRCT | TB | July 2016-January 2020 (January 2018 -July 2018 “washout” period) (3y6m) | 2501 / 1562 | Public primary care clinic / Sampling strategy was not recorded. All patients who started TB treatment in the 2 months prior to the study start through study end (apart from the washout period) and identified contacts. | Household-based: 39 (29-48), incentive-based: 37.5 (28-49). | NR | Black African: 99%, Mixed race: <1%, White: <1%, Indian or Asian: <1% | 42.5 |
| Hanrahan, 2023;^23^ South Africa | RCT | TB | July 2016-January 2018 (1y6m) | NR / 3655 | Public primary care clinic and community (home visits) / The largest clinics per district in which study inclusion was deemed feasible were selected (including willingness to participate, quality of record keeping, and geographic location). The control arm (facility based) included all clinic attendees presenting with TB symptoms. The intervention arm included TB index patients who had started treatment within the 2 months prior to recruitment. | Intervention: 37 (29-48), control: 38 (28-49). | NR | NR | 42.0 |
| Heumann, 2017;^24^ USA | OCC (retrospective) | Early syphilis (primary, secondary or early latent) and HIV | 2010-2014 (4y) | Total 2979 / 2174 | Clinic and community / The patient register was searched for all King County, Washington residents reported to PHSKC with early syphilis or newly diagnosed HIV infection who were interviewed by DIS for PS between 2010 and 2014. Exclusion: OPs with incomplete interviews. | Syphilis: in person 37 (28-45), telephone 41 (32-48). HIV: in person 32 (25-41), telephone 36 (28-46). | NR | White: syphilis 66.3%, HIV 58.8%; Black: syphilis 9.6%, HIV 15.5%; Latino: syphilis 12.0%, HIV 15%; Asian: syphilis 3.0%, HIV 5.9%; Other: syphilis 5.6%, HIV 5.2%. | Syphilis: 2.5, HIV: 10.0 |
| Hu, 2021;^25^ China | RCT | HIV | August 2017-January 2019 (1y5m) | 194 / 187 | Clinic / Cisgender male clinic attendees ≥18 years old who self-reported oral or anal intercourse with male partners in past six months and were diagnosed with HIV in the past three months. Attendees were excluded if they had already notified sexual partners of their HIV status. | 29.2 (24.6-36.1) | Education: high school and below 33.7%, college and above 66.3%. Occupation: full time 57.8%, part time 16.0%, unemployed 15.0%, student 11.2%. Monthly income USD: equal to or <430 32.1%, 431-720 40.1%, >720 27.8%. | NR | 0.0 |
| Huang, 2019;^26^ Taiwan | CBA | TB | January 2006-December 2013 (7y11m) | NR / 1712 | Unclear / Regions were chosen based on TB incidence rate and death rate, and patient registers were searched for recruitment. | 0-20: 8.5%, 21-30: 7.8%, 31-40: 14.4%, 41-50: 21.1%, 51-60: 21.6%, 61-70: 12.9%, 71-80: 9.1%, 81-90: 4.4%, 91-100: 0.2% | NR | NR | 31.3 |
| Jerene, 2022;^27^ Ethiopia | CBA | TB | July 2020 and June 2021 (2m) | NA / 8922 | Community / A patient register was used for recruitment. Criteria were not reported. | NR | NR | NR | NR |
| Jose, 2020;^28^ Mozambique | CBA | TB | Baseline (before): Q4 2016-Q3 2017, intervention (after):  Q4 2017-Q3 2018 (18m) | NA / NA | Community / Walk-in patients entering health centres were screened for TB, and household contacts of TB cases were identified through home visits. | NR | NR | NR | NR |
| Joshi, 2015;^29^ Nepal | CBA (retrospective) | TB | March 2012-March 2014 (2y) | NA / NR | Community / Patient registers were searched for children aged 0–14 years diagnosed and registered with TB in intervention and control districts. | NA | NA | NA | NA |
| Kagujje, 2023;^30^ Zambia | cITS | TB | Pre-intervention: January 2018-September 2019, intervention: January 2020-September 2021 (3y4m) | 5150 / 5150 | Public hospitals / District notification reports were searched for all childhood (<15 years of age) TB case notifications before and during implementation of a multicomponent strategy to bolster childhood TB diagnosis at two intervention and two control sites. | Mean (SD): intervention 3.4 (4.0) | NR | NR | Intervention: 48.8 |
| Kaswaswa, 2022;^31^ Malawi | cRCT | TB | May 2014-December 2014 (7m) | 464 / 214 | Clinic / Clinic attendees who: were never previously treated for TB and ≥18 years old on day of TB registration; had at least one child aged five years or younger residing in their households (this criteria was removed in a subsequent protocol amendment); were resident within Blantyre; and if an in-patient, were likely to be discharged within two weeks. Excluded: hospitalised patients unlikely to be discharged within 14 days or those with membership of a household already recruited. | Mean (range): intervention: 33.5 (19-73), control 32.0 (18-70) | Education: no school 3.3%, primary 39.4%, secondary 53.5, higher 3.7% | NR | 35.7 |
| Katz, 1988;^32^ USA | RCT | STI (chlamydia, NGU) | May 1985 -December 1985 (7m) | NR / 678 | STD clinic / Male clinic attendees presenting with NGU. Excluded: partners who had been treated in the clinic prior to the index patient's visit. | Mean: 26 | NR | Black: 63% | 0.0 |
| Kerani, 2011;^33^ USA | RCT | Chlamydia, gonorrhoea | July 2007-March 2009 (1y8m) | 393 / 75 | Clinic and internet / A patient register was searched for recruitment. Exclusion criteria: <18 years of age; did not speak English; reported that all partners were treated; had not had sex with a man in the 60 days preceding diagnosis; case report received >2 weeks after patient's treatment; or diagnosed with HIV or syphilis in the 90 days before their gonorrhoea or chlamydia diagnosis. | Mean (SD): 31.1 (9.0) | NR | White: 76.9%, Asian or Pacific Islander: 7.7%, Latino: 5.8%, Black: 5.8% | 0.0 |
| Ketema, 2020;^34^ Ethiopia | RCT (stepped wedge) | TB | August 2016-November 2017 (1y3m) | 181 455 / 1603 | Health centre / Attendees at health centres in Addis Ababa seeing more than 500 children under 5 per year. | NR | NR | NR | NR |
| Khatana, 2019;^35^ India | Quasi RCT | TB | 1^st^ January 2014-30^th^ June 2015 (1y6m) | 282 / 282 | Community / Attendees at community TB units with bacteriologically confirmed index PTB along with their household contacts (≥10 years of age), including transferred-in cases, registered in TB-Unit from 1^st^ January 2014 to 31^st^ December 2014. Excluded: not permanent residents of the geographical area of selected TB Unit. | Mean (SD): 42 (19.9) | Education: illiterate: 45.0%, literate and to 5th standard: 14.2%, 6^th^-10th standard: 28.0%, 11^th^-graduate: 12.4%, postgraduate: 0.4%. Occupation: agricultural cultivator: 11.3%, business: 4.6%, govt./private service: 7.4%, homemaker: 22.3%, non- agricultural & skilled worker: 9.6%, student: 17.0%, unemployed: 6.0%, old age (> 60 years): 21.6%. | NR | 53.9 |
| Kissinger, 2005;^36^ USA | RCT | STI (urethritis, chlamydia, gonorrhoea) | December 2001-March 2004 (2y3m) | 1148 / 977 | Public STD clinic / Male attendees of the public STD clinic in Louisiana during the study period who: were diagnosed with urethritis; had a test positive for chlamydia or gonorrhoea; were 16–44 years old; and had at least 1 female sex partner who did not accompany them to the clinic. | <24: 48.3% | High school educated: 77.4% | African American: 95.7% | 0.0 |
| Kissinger, 2006;^37^ USA | RCT | Trichomoniasis | December 2001-August 2004 (2y8m) | NR / 463 | Clinic and community / Female clinic attendees who: had a culture-confirmed diagnosis of TV; were not in their first trimester of pregnancy; had no medical contraindication to taking metronidazole or bringing metronidazole to a partner; and had at least 1 male sex partner in the last 60 days. | Mean: 25.8 (SD 6.8) | High school education or greater: 87.3% | Black: 99.1% | 100 |
| Landis, 1992;^38^ USA | RCT | HIV | November 1988-June 1990 (11y7m) | 162 / 74 | Three large county health departments / Clients with HIV identified through HIV counselling and testing programs and other health department clinics. Excluded: clients who: did not return for test results and could not be located; previously tested positive for HIV with no new sex or needle-sharing partners; only had partners whose names they did not know; had had no sex or needle-sharing partners in the past year; or lived outside or had partners who lived outside the jurisdictions of the three county health departments. | Mean: 30 | NR | Black: 87% | 31.0 |
| Low, 2006;^39^ England | RCT | Chlamydia | March 2001-October 2002 (1y6m) | 190 / 140 | Community / Men and women with a positive chlamydia test who received the result at their general practice, randomly selected from the patient register. | 16-19: 38.5%, 20-24: 50%, 25-39: 11.5% | Deprivation in ward of residence: low: 38.5%, medium: 34%, high: 25.5%, missing data: 1.25% | White: 81.5%, Black Caribbean: 5%, Black Other: 2.5%, Indian, Pakistani or Bangladeshi: 1%, Chinese or Other: 2%, Missing data: 8% | 65.5 |
| Lugada, 2010;^40^ Uganda | cRCT | HIV | February 2005-February 2007 (2y) | NR / 1453 | Clinic and community / A patient register was used for recruitment. Criteria were not reported. | NR | NR | NR | NR |
| Lukac, 2021;^41^ Canada | OCC | Syphilis | 1^st^ January 2016-31^st^ December 2016 (1y) | 648 / 281 | Community / A patient register was used to recruit all patients diagnosed with infectious syphilis between 1^st^ January and 31^st^ December 2016 in BC who resided in BC and identified as gbMSM. | Median (range): 38 (17-86) | NR | NR | 0 |
| Luo, 2020;^42^ China | OCC | HIV | June 2014-June 2016 (2y) | 2495 / 466 | VCT clinic / Male clinic attendees: ≥18 years old; newly diagnosed as HIV positive within one month; and disclosed male sexual partners to VCT staff in pre- or post-testing counselling at the time of diagnosis. Included contacts: individuals reported to be sexual partners of ICs in post-testing counselling at the time of HIV testing. Index cases and contacts were excluded if they had cognitive impairment, a severe AIDS complication or other illnesses requiring hospitalization, or disabilities. | Mean: 39.5 (SD 9.6) | Education level:  primary school or below: 7.0%,  middle school: 19.5%,  high school: 23.3%,  college or above: 47.5%,  missing: 2.7% | NR | 0 |
| Malave, 2008;^43^ USA | OCC | HIV | January-December 2004 (11m) | 3666 / 3666 | STD clinic and other diagnosing facilities including hospital / HIV index case-patients newly diagnosed in STD clinics and non-STD facilities in 2004. Exclusion: index case-patients diagnosed in DOHMH-run tuberculosis clinics and NYC jail clinics from the non-STD facilities. | Mean (SD): STD clinic group: 33.2 (9.7),  non-STD facility: 38.0 (11.6) | Neighbourhood income category:  <20% below poverty line: 31.55%, ≥20% below poverty line: 61.75%, unknown: 6.75% | Black: 59.1%,  Hispanic: 26.5%,  White: 10.65%,  Other/unknown: 3.75% | 25.45 |
| Malheiro, 2020;^44^ Portugal | OCC | Covid-19 | 1^st^ March 2020-30^th^ April 2020 (2m) | 630 / 551 | Community / A public health authority database was used to source all Covid-19 confirmed cases that were either identified as close contacts of an index case or returned from affected areas and placed under mandatory quarantine, with daily follow-up until laboratory confirmation of SARS-CoV-2 infection. Control cases included all COVID-19 confirmed cases that were not subject to CT nor to quarantine measures preceding the diagnosis. Control cases included all Covid-19 confirmed cases that were not subject to CT nor to quarantine measures preceding the diagnosis. | Intervention: 50.5 (34.2-66), control 53 (36-67) | NR | NR | 58.8 |
| Martinson, 2022;^45^ South Africa | cRCT | TB | December 2016-March 2019 (2y3m) | 2393 / 2062 | Community / Clinic attendees with TB of any age (but those aged ≥7 years must have laboratory-confirmed pulmonary TB) and TB patients who died within 8 weeks of TB diagnosis. Excluded: institutionalized TB patients and those whose households we could not locate or from where no household member could be recruited. | 37 (28-48) | Employment:  currently employed: 17.5%, not employed: 67.5%, student/child: 8.5%, other: 6%. Income type: salary: 17%, wage: 5.5%, grant: 22%, no income: 55.5%. | NR | 40.0 |
| Mathews, 2020;^46^ South Africa | RCT | STIs (no further specifics given) | June 2014-August 2017 (3y2m) | 1434 / 1050 | Community STI clinic / Clinic attendees ≥18 years old diagnosed with STI using a syndromic approach. | Mean: 29.7 | High school completion: 45.03% | NR | 49.8 |
| Morishita, 2016;^47^ Cambodia | Quasi RCT | TB | February 2012-December 2012 then May 2013-March 2014 (1y8m) | NA / NA | Community / Patient registers were searched from 30 ODs with high TB case notification rates for smear-positive TB (>125 per 100,000 population) and high poverty/access-barrier composite scores. | NA | NA | NA | NA |
| Oh, 1996;^48^ USA | nrCT | Chlamydia, gonorrhoea | December 1990-April 1993 (2y4m) | 265 / 363 | Urban non-STD clinic / Adolescent female clinic attendees with positive gonorrhoea or chlamydia cultures. | Range 12-19 | NR | Data for whole clinic not just study sample: African American: 85%, White: 15%, Other: <0.1% | 100 |
| Ostergaard, 2003;^49^ Denmark | RCT | Chlamydia | February 1999-March 2000 (1y1m) | NR / 1826 | Community / A patient register was searched for patients with a swab sample positive for chlamydia who chose to submit the study questionnaire. | Mean 23.7 | NR | White: 100% | 73.70 |
| Parkes-Ratanshi, 2020;^50^ Uganda | RCT | Syphilis | February 2015-February 2016 (1y) | 452 / 442 | Antenatal clinic / Female clinic attendees: with a positive pregnancy test and treponemal antibody rapid POCT; aged >18 years or 14-17 years and being a mature and emancipated minor; having a known sexual partner; having access to a cell phone; and being willing and able to use and receive SMS or phone calls. Excluded if: illiterate; having confirmed neurosyphilis; or attending with their partners, as male partners were notified and treated for syphilis on the same day. | 26 (23-30) | Education: none: 0.2%, primary: 41.9%, secondary: 49.8%, tertiary: 7.7%, missing: 0.5%. Currently employed: yes 53.2%. | NR | 100.0 |
| Peterman, 1997;^51^ USA | RCT | Syphilis (primary, secondary or early latent) | December 1990-March 1993 (2y3m) | 1966 / 1966 | Community / Syphilis cases were reported from multiple sources: STD and non-STD clinics, jail, hospitals, prenatal/obstetric and other settings. All people with primary, secondary, or early latent syphilis in Broward County, Florida; Tampa, Florida; and Paterson, New Jersey were included. | Mean: 33 | NR | Black: 79% | 47.0 |
| Potterat, 1977;^52^ USA | Controlled trial | Gonorrhoea | February-September 1975 (7m) | 187 / 187 | Public health clinic / All heterosexual male clinic attendees with gonorrhoea diagnosed at the El Paso City-County Health Department (Colorado). | NR | NR | NR | 0 |
| Raymenants; 2022,^53^ Belgium | OCC | Covid-19 | 1^st^ February 2021-31^st^ May 2021 (4m) | NA / 4522 | Community / A patient register was searched for students with either a positive RT-qPCR test result at the KU Leuven test centre or reported to the tracing team as having had a positive RT-qPCR test result elsewhere and had recently resided in or had come into contact with others in the city of Leuven. Exclusion intervention: if the treating physician interpreted the result as falsely positive, or as a past infection with Covid-19, or case could not be contacted by the tracing team after repeated attempts, or information on symptom onset was missing. Exclusion contacts: already identified as contacts exposed to a previously diagnosed index case within 7 days before the CT interview were excluded as contacts from the second identified index case, while still being considered as contacts for the first. Contacts who had already tested positive on the same day as the index case or up to 60 days before, were also excluded. Inclusion control: all students who attended the test centre for the first time during the study period, and who self-reported symptoms suggestive for Covid-19 as the reason for their test. | Mean 21.4 (SD 2.30) | NR | NR | 48.9 |
| Schwebke, 2010;^54^ USA | RCT | Trichomoniasis | February 2003-June 2008 (5y4m) | NR / 484 | Clinic / Female clinic attendees ≥19 years old attending the Jefferson County Department of Health in Birmingham, AL, with a culture or wet prep positive for TV. Exclusion: infection with other STD pathogens requiring Health Department DIS intervention; pregnancy or currently breast feeding; recent (8 hours) ingestion of alcoholic beverages or intent to do so in the 24 hours following treatment; allergy to metronidazole; presence of a sexual partner in the clinic at the time of enrolment; history of referral by a partner already treated for trichomoniasis; or report of more than 4 sexual partners in the preceding 30 days. | Mean (SD): 28.8 (8.0) | NR | African American: 94.8%, White: 4.1%, Other 1.0% | 100.0 |
| Shah, 2020;^55^ Peru | cRCT (stepped wedge) | TB | November 2012-June 2014 (1y7m) | NR / 3222 | Community / Clinic attendees newly diagnosed with TB ≥16 years old enrolled in a SJL NTP healthcare centre between 1^st^ November 2012 to 30^th^ June 2014 with at least one household contact. | Mean: 30.4 | Education level: none: 1.6%, primary school: 13.2%, high school: 66.9%, technical degree: 1.7%, university degree: 16.3% | NR | 36.9 |
| Tchakounte, 2024;^56^ Cameroon | cRCT | Covid-19 | October 2022-March 2023 (5m) | 431 / 313 | Community / The Mamal PRO app register was searched for individuals who tested positive for SARS-CoV-2 in selected testing sites who registered in the Mamal PRO app for testing and had identified contacts within the last 14 days prior to the test date. Exclusion: no contacts declared. | 35 (28-49) | NR | NR | 61.0 |
| Wilson, 2009;^57^ USA | RCT | Chlamydia, gonorrhoea | January 2002-December 2004 (2y11m) | 1113 / 600 | STI clinic / Clinic attendees with a microbiologically confirmed diagnosis of chlamydia or gonorrhoea who were: ≥18 years old; able to complete an interview in English or Spanish; diagnosed with chlamydia or gonorrhoea within the previous 2 weeks; sexually active in the previous 2 months; and residing in NYC area for the evaluation period. | Mean (SD): 25 (6.7) | Completed high school: 62%. Full time employed: 42%. | African American: 40%, Afro-Caribbean: 52.5%, Other: 7.5%, Hispanic: 10.5% | 41.0 |
| ACT, Australian Capital Territory; AIDS, acquired immunodeficiency syndrome; AL, Alabama; ANC, antenatal clinic; ART, antiretroviral therapy; BC, British Columbia; CBA, controlled before and after study; cITS, controlled interrupted time series; CT, contact tracing; cRCT, cluster randomised controlled trial; DIS, disease intervention specialists; DOHMH, Department of Health and Mental Hygiene; gbMSM, a male who reported male sexual partners only or both male and female sexual partners; govt., government; HIV, human immunodeficiency virus; HIVST, human immunodeficiency virus self-testing; IC, index case; IQR, interquartile range; KU, Katholieke Universiteit; MSM, men who have sex with men; NA, not applicable; NGU, non-gonococcal urethritis; No., number; nrCT, non-randomised controlled trial; NR, not reported; NTP, National Tuberculosis Program; NYC, New York City; OCC observational with concurrent control; OD, operational district; OP, original patient; POCT, point-of-care test; PS, partner services; PTB, pulmonary tuberculosis; PHSKC, Public Health – Seattle and King County; RCT, randomised controlled trial; RT-qPCR, quantitative reverse transcription polymerase chain reaction; SARS-CoV-2, severe acute respiratory syndrome coronavirus 2; SJL, San Juan de Lurigancho; SMS, short messaging service; SD, standard deviation; STD, sexually transmitted disease; STI, sexually transmitted infection; TB, tuberculosis; TV, Trichomonas vaginalis; TW, transgender women; UK, United Kingdom; USA, United States of America; USD, United States dollar; VCT, voluntary counselling and testing. | | | | | | | | | |

Table S2. Details of deprioritised studies

| **Author, date** | **Study design** | **Country (of study)** | **Sample size (intervention, control)** | **Infectious disease** | **Names of study arms** | **Summary of key findings** |
| --- | --- | --- | --- | --- | --- | --- |
| Adejugbagbe, 2022^58^ | UBA: 3 months prior vs 3 months during | Nigeria | 3927 (NR) | Covid-19 | Decentralisation of Covid-19 response to local government areas | Across all three intervention areas, only numbers tested, and the number of incident management meetings increased during the study period. The positivity rate, contacts line-listed and reported cases reduced following the intervention. |
|  |  |  |  |  | SOC (centralised response) |  |
| Beebeejaun, 2021^59^ | Self-controlled before and after study | England | 752 (650, 102) | (Chronic) Hepatitis B | Nurse-led enhanced management and CT | There was an improvement in case referral rates by an additional 14% (from 86% (88/102 cases) to 99.7%; 648/650 cases) with implementation of the intervention. There was an increase in the number of contacts tested from 34% to 72%-94% with 18 new cases of HBV diagnosed. During the study vaccination rates of at least three doses, amongst close contacts, increased from 77% (43/56) to 93% (452/491). |
|  |  |  |  |  | SOC |  |
| Djuric, 2024^60^ | UBA | Italy | 1604 (1394, 490) | Covid-19 | Improved CT protocols | Implementation of prompt CT was associated with a 10% decrease in the number of secondary cases (excess relative risk –0.1 (95% CI: –0.35 to 0.15). |
|  |  |  |  |  | SOC |  |
| Duarte, 2012^61^ | UBA | Portugal | 877 total TB patients (2001-2003: 502; 2004-2006 375) | TB | Home and workplace visits (extended TB CT/screening) | With the intervention more patients with active TB and LTBI were detected (1.4 per index patient compared with 0.75 per index patient previously), and more TB cases were prevented. |
|  |  |  |  |  | SOC - Interview with newly diagnosed IC - only named contacts screened |  |
| Dzhangaziev, 2020^62^ | UBA | Kyrgyzstan | 5778 (3325,2453) | TB | Expanded CT | The new TB tracing strategy resulted in increased numbers of indexes (21%) and contacts (36%). Though a smaller number of contacts (1730 vs. 1590) were screened in 2018, the proportion of TB diagnosed was substantially higher (95% CI: 0.024 to 0.005, p=0.002) in 2018 vs 2017. |
|  |  |  |  |  | Limited CT |  |
| Edmiston, 2010^63^ | UBA (pre-int vs during int) | Australia | 70 (NR) | Chlamydia | 'Make Contact' card for ICs and contacts + social media campaign | No differences were found in the notification index (1.83 vs 1.91, p=0.74) or treatment index (0.94 vs 0.91, p=0.89) in the pre-intervention and the intervention phases. |
|  |  |  |  |  | SOC (no card) |  |
| Engelgau, 1995^64^ | UBA | USA | 800 (373, 427) | Syphilis (early) | Campaign (the number of public health workers assigned to syphilis control activities and hours of STD clinical services increased from the pre-campaign level) | Comparing the early campaign with the pre-campaign periods, there was a greater average number, per case, of infected persons identified (0.48 vs 0.37, p=0.66) and persons prophylactically treated (3.9 vs 2.5, p< 0.01). |
|  |  |  |  |  | SOC (standard staffing levels) |  |
| Fatima, 2016^65^ | UBA | Pakistan | NR | TB | Close contact screening (TB screening of all people staying within 50m of household of index case + household) | The intervention contributed an increase of case detection of bacteriologically confirmed tuberculosis by 6.8% and all forms TB patients by 7.9%. |
|  |  |  |  |  | HHC screening only |  |
| Fiumara, 1953^66^ | UBA | USA | 297 (126, 171) | Venereal diseases/ STIs (gonorrhoea, syphilis) | Specialised nurse interviewers (for CT) | The trained nurses obtained 1.29 contacts for gonorrhoea and 3.46 contacts for syphilis compared to military interviewers who obtained 1.13 contacts per patient with gonorrhoea and 1.58 contacts for lesion syphilis (see paper for further data including treatment numbers). |
|  |  |  |  |  | Untrained/unspecialised military interviewers |  |
| Fu, 2016^67^ | UBA | China | 253 (148, 105) | HIV | Sexual PN and HIV testing mobilisation | Compared with previously identified ICs, the proportion of contactable sexual partners of newly identified ICs was higher, but the testing rate was lower (p<0.001). There was no statistical difference in the number of reactive HIV among tested sexual partners in the process initiated by newly identified primary ICs compared to previously identified primary ICs (13.0% vs 6.6%, p=0.09 in the whole process). |
|  |  |  |  |  | SOC (no active PN or testing initiatives) |  |
| Grande, 2021^68^ | UBA | Botswana | 6440 (1265, 5175) | HIV | Intensified assisted PS training, supervision and data collection | Between pre and post intervention phases, there was an increase in the percentage of cases with an identified HIV-positive partner from 12.6% to 60% (p=0.001, all outcomes). In phase 1, the testing index (partners tested per case) was higher (0.56 vs 0.45, p=0.05), whereas the case-finding index did not change (partners testing HIV-positive per case) (0.13 vs 0.14, p=0.50). |
|  |  |  |  |  | SOC (minimal assisted PS training, supervision and data collection) |  |
| Jones, 2021^69^ | UBA | USA | 184 (85, 99) | Chlamydia | Adapted Check It (contact screening program) for African American men aged 15-24y | In post-adaptation phase female sexual partners were significantly more likely to complete treatment (n=153) compared to pre-adaptation (n=161) (RR=3.02, 95% CI: 1.81 to 5.05). |
|  |  |  |  |  | SOC (original, unchanged Check It program) |  |
| Kantor, 2021^70^ | UBA | USA | 100 (52, 48) | HIV | In cluster | Reinterviewing did not improve CT beyond initial interviews, and the study was stopped early for futility (see paper for further data). |
|  |  |  |  |  | Not in a cluster |  |
| Kingbo, 2020^71^ | Cohort (no concurrent control) | Cote d'Ivoire | 1089 (NR) | HIV | Client referral (ICs refer the contacts for HIV testing) | From 1,089 newly diagnosed ICs there were 1,089 sex partners identified and 469 children. Approximately 90% of children were contacted through client referral: 85.2% of those were tested and 1.4% was positive. 56.3% of sex partners were contacted through provider referral, and 97.2% were HIV-tested and 75.5% positive. 30% of sex partners were contacted through contract referral and 81.5% were HIV-tested and 72.7% found to be positive. Provider referral is a successful and acceptable strategy for bringing in sex partners for testing. Client referral is preferred for children. |
|  |  |  |  |  | Provider referral (health care providers refer the contacts) |  |
|  |  |  |  |  | Contract referral (IC-provider hybrid approach) |  |
|  |  |  |  |  | Dual referral (both the index and their partner are tested simultaneously) |  |
| Learner, 2021^72^ | Awaiting classification | USA | 1242 (NR) | Gonorrhoea | Traditional PS + enhanced CT (interviewing and testing sexual partners of persons who tested negative for gonorrhoea, and social contacts of ICs and partners) | 1,088 sexual partners were identified through traditional methods and 105 were newly diagnosed with gonorrhoea. An additional 59 sexual partners and 52 social contacts were identified through enhanced PS and of those partners and contacts, 3 were newly diagnosed with gonorrhoea. |
|  |  |  |  |  | Traditional PS (counselling individuals diagnosed with an STD and identifying their sexual partners, interviewing, testing, and treating partners, and providing PS to those who are newly diagnosed) |  |
| Mahachi, 2019^73^ | UBA | Zimbabwe | 25704 | HIV | Zimbabwe HIV Care and Treatment project - index testing and PN services in the community | During the first six months, there was a 10% increase in the average HIV positivity rate which rose to more than 30% by August 2016 and this level was sustained above 30% through to May 2018. The overall facility HIV positivity rate was 4.1% during the same period. |
|  |  |  |  |  | SOC (no specific community PS) |  |
| Martinez-Camprecios, 2024^74^ | UBA | Angola | 969 (NR) | TB | Medical staff reinforcement | Engagement in the programme was low, although during the community DOT period there was a significant increase. Of 457 contacts younger than 5 years, 36 (7.9%) received preventive TB treatment. There was a high rate of contacts lost to follow-up before a final decision was taken on treatment. |
|  |  |  |  |  | Routine staff |  |
|  |  |  |  |  | Community DOT with community worker support |  |
|  |  |  |  |  | Enhanced CT |  |
| Ospina, 2012^75^ | UBA | Spain | 960 (388, 572) | TB | Community health workers CT among immigrants | During the pre-intervention period CT was performed on 65.7% of 201 smear-positive cases compared to 81.6% of 152 smear-positive cases during the intervention period (p<0.001). Several risk factors were identified to be associated with incomplete CT of smear-positive ICs. |
|  |  |  |  |  | SOC (public health nurse-led CT) |  |
| Osterlund, 2005^76^ | UBA | Sweden | 719 (676, 43) | Chlamydia | Specialist contact tracers and genetic sequencing | The year prior to the study, in two municipalities CT revealed 1.6 partners for each IC (n=110). During the study period the figure for the whole county was 2.1 partners per IC (n=2065). Valuable information in mapping individual sexual networks was added with genotyping, this however was not essential for achieving satisfactory CT results. |
|  |  |  |  |  | SOC (non-specialised CT staff and no genetic sequencing) |  |
| Qader, 2017^77^ | Dose-response | Afghanistan | 24619 (NR) | TB | Urban DOTS + contact investigation | Between 2009 and 2015, the number of DOTS-providing centres in Kabul increased from 22 to 85. The case notification rate for all forms of TB increased from 59 per 100,000 population to 125 per 100,000. There was an increase in treatment success rate for all forms of TB increased from 31% to 67%. The treatment success rate for private HFs increased from 52% in 2010 to 80% in 2015. When contact screening was introduced in 2013, the TB yield was 723 per 100,000, more than two times higher than the estimated national prevalence of 340 per 100,000. Of the 2,509 child contacts identified through CT, 76% received IPT. Service accessibility, TB case finding, and treatment outcomes were significantly improved in Kabul with the comprehensive urban DOTS program. |
|  |  |  |  |  | SOC (generic DOTS) |  |
| Rudy, 2012^78^ | UBA | USA | 151 (87, 64) | Syphilis | After CEDIS placement | In 2009-2010 after placement of the CEDIS, 15% (21) of 140 partners elicited were identified with early syphilis and brought to treatment compared with 0% of 13 partners elicited before placement of the CEDIS (2006-2007) and 15% (25) of 171 partners elicited countywide. |
|  |  |  |  |  | Before CEDIS placement |  |
| Sanaie, 2016^79^ | UBA | Afghanistan | 2022127 (NR) | TB | Systematic screening at health facilities | 2,022,127 people for TB symptoms were screened during the intervention, 59,838 tested and 5,046 detected people with smear-positive TB. Among tested individuals, 46,763 (78.1%) were HF attendees, 8,836 (14.8%) were IDPs, and 4,239 (7.1%) were HHCs. There was a 56% increase in smear-positive TB notifications observed between the baseline and intervention periods among the 47 HFs, where cases detected by all three strategies were notified. There was a significant increase in the proportion of screened individuals who were tested among HHC and HF attendees, but this significantly decreased among IDPs between years 1 and 2 (p<0.001 for all strategies). |
|  |  |  |  |  | Active HHC investigation |  |
|  |  |  |  |  | ACF through outreach and screening at IDP camps |  |
|  |  |  |  |  | SOC (passive case finding) |  |
| Schleihauf, 2019^80^ | UBA (16 months prior vs 19 months post | Canada | 343 (220, 123) | Gonorrhoea | Enhanced CT including NG-MAST | The number of reported gonorrhoea cases per month significantly increased when comparing the preintervention and postintervention periods. The reported gonorrhoea rate in 2016 was 2.9 times higher than in 2014. This increase was not associated with changes in testing rates and was more pronounced among women than men. |
|  |  |  |  |  | Standard CT without NG-MAST/standard practice |  |
| Sulis, 2018^81^ | UBA | Burkina Faso | 3717 (NR) | TB | Active CT | Active tracing led to a substantially higher contact/index ratio (1.83) and screening coverage (99.3%, 145/146). |
|  |  |  |  |  | SOC (passive case finding) |  |
| Taylor, 2010^82^ | UBA | USA | 334 (115, 219) | Syphilis | DIS placed in HIV clinics to improve partner elicitation interviews | After the placement of clinic based DIS, there were increases in the average number of locatable partners (1.1 after vs 0.6 before, p=0.004). The average number of partners exposed and brought to treatment or infected and brought to treatment also increased (0.6 after vs 0.3 before, p=0.02). |
|  |  |  |  |  | SOC (no DIS in clinics) |  |
| Tembo, 2019^83^ | UBA | Malawi | 6408 (2628, 3780) approximate calculation | HIV | Behavioural skills-building training aimed at improving the ability of healthcare workers to deliver passive referral IC-finding programme. | 200 facility-months were observed before and 124 facility-months observed after training. During the ten-month study period the mean number of contacts who received HIV testing (pre=11.1, post=24.8, p<0.001) and the mean number of HIV-positive contacts identified per facility-month (pre=1.3, post=2.3, p<0.001) increased. |
|  |  |  |  |  | SOC (no behavioural skills training) |  |
| Tembo, 2021 ^84^ | UBA | Malawi | 983 (604, 379) approximate calculation | HIV | Blended learning package combining digital and face-to-face training for index testing services | Index testing effectiveness indicators increased between pre and post periods: indexes screened (pre=63, post=101, p<0.001); contacts elicited (pre=75, post=131, p<0.001); and contacts who received HIV testing (pre=27, post=41, p=0.014). In the post-period the mean number of contacts tested per facility per month was 1.6 times higher compared to the pre-period. There was an indication towards more contacts newly testing as HIV-positive per facility per month, but this was not a statistical difference (pre=4.0, post=7.5, p=0.409). |
|  |  |  |  |  | SOC (no training) |  |
| Udeagu, 2012^85^ | UBA | USA | 2703 (1280,1423) | HIV | HIV Field Services initiative | In 2005 partner service outcomes (partner elicited, notified, and tested) were similar at participating and nonparticipating sites. In 2008 significant differences in key partner service outcomes were observed between participating and nonparticipating sites in the post-intervention period. |
|  |  |  |  |  | SOC |  |
| Van de Berg, 2021^86^ | UBA | Netherlands | 3192 (1489, 1703) | TB | After guideline adaptation (stone in the pond principle of contact investigation) | Of 1,703 and 1,489 index patients reported (pre and post), 27,187 and 21,056 contacts were eligible for contact investigation, 86% and 89% were tested for TB, and 0.70% and 0.73% were identified with active TB. |
|  |  |  |  |  | SOC (before guideline) |  |
| Volkmann, 2016^87^ | UBA (single time point 6 months before and 6 months after) | Kenya | 183 (75, 108) | TB | Changes to CT process | A 42% increase in the proportion of ICs traced was found between retrospective and prospective periods (p<0.001) and from the contact investigations instigated, this gave an average of 3.0 and 2.6 contacts identified per IC respectively (p=0.14). There were 22 (12%) symptomatic contacts during the retrospective period and 17 (10%) during the prospective period (p=0.85) and of these, the proportion referred increased from 64% to 82% after initiation of the CT intervention (p<0.001) and of those eligible for treatment, the proportion that started IPT increased from 19% to 27% (p=0.15). |
|  |  |  |  |  | SOC |  |
| Zachariah, 2003^88^ | UBA | Malawi | 189 (87, 102) | TB | Active case finding | 985 HHCs were found from 189 TB ICs. The prevalence of TB by passive case finding in the 524 HHCs (0.19%, 191/100 000) was significantly lower than with active finding among 461 contacts (1.74%, 1735/100 000) (p=0.01). In the passive cohort 126 contacts were children and of these, 22 (17%) received treatment compared to the active cohort where 25 (22%) of 113 children received the drug. |
|  |  |  |  |  | SOC (passive case finding) |  |
| ACF, active case finding; CEDIS, community-embedded disease investigation specialist; CI, confidence interval; CT, contact tracing; DIS, disease intervention specialists; DOT, directly observed treatment; DOTS, directly observed treatment, short-course; HBV, hepatitis B virus; HF, health facility; HHC, household contact; HIV, human immunodeficiency virus; IC, index case; IDP, internally displaced people; IPT, isoniazid preventive therapy; LTBI, latent tuberculosis infection; NG-MAST, multiantigen sequence typing; NR, not reported; PN, partner notification; PS, partner services; RR, relative risk; SOC, standard of care; STD, sexually transmitted disease; STI, sexually transmitted infection; TB, tuberculosis; UBA, uncontrolled before and after; USA, United States of America; vs, versus. | | | | | | |

# References

1. Andersen B, Ostergaard L, Moller JK, Olesen F. Home sampling versus conventional contact tracing for detecting Chlamydia trachomatis infection in male partners of infected women: randomised study. *BMJ (Clinical research ed)* 1998;**316**:350-1. <https://doi.org/10.1136/bmj.316.7128.350>

2. Apoola A, Beardsley J. Does the addition of a urine testing kit to use of contact slips increase the partner notification rates for genital chlamydial infection? *International journal of STD & AIDS* 2009;**20**:775-7. <https://doi.org/10.1258/ijsa.2009.009196>

3. Bai LQ, Yang HL, Jian XW, He XG, Chen YF, Tang Y*, et al.* Increasing tuberculosis case detection through intensive referral and tracing in Hunan, China. *Int J Tuberc Lung Dis* 2008;**12**:1431-5.

4. Brown LB, Miller WC, Kamanga G, Nyirenda N, Mmodzi P, Pettifor A*, et al.* HIV partner notification is effective and feasible in sub-Saharan Africa: opportunities for HIV treatment and prevention. *J Acquir Immune Defic Syndr* 2011;**56**:437-42. <https://doi.org/10.1097/qai.0b013e318202bf7d>

5. Cavalcante SC, Durovni B, Barnes GL, Souza FBA, Silva RF, Barroso PF*, et al.* Community-randomized trial of enhanced DOTS for tuberculosis control in Rio de Janeiro, Brazil. *Int J Tuberc Lung Dis* 2010;**14**:203-9.

6. Chen JS, Matoga M, Pence BW, Powers KA, Maierhofer CN, Jere E*, et al.* A randomized controlled trial evaluating combination detection of HIV in Malawian sexually transmitted infections clinics. *Journal of the International AIDS Society* 2021;**24**:e25701. <https://doi.org/10.1002/jia2.25701>

7. Cherutich P, Golden MR, Wamuti B, Richardson BA, Asbjornsdottir KH, Otieno FA*, et al.* Assisted partner services for HIV in Kenya: a cluster randomised controlled trial. *Lancet HIV* 2017;**4**:e74-e82. <https://doi.org/10.1016/S2352-3018(16)30214-4>

8. Chiou P-Y, Lin L-C, Chen Y-M, Wu S-C, Lew-Ting C-Y, Yen H-W*, et al.* The effects of early multiple-time PN counseling on newly HIV-diagnosed men who have sex with men in Taiwan. *AIDS and behavior* 2015;**19**:1773-81. <https://doi.org/10.1007/s10461-015-1007-0>

9. Choko AT, Fielding K, Johnson CC, Kumwenda MK, Chilongosi R, Baggaley RC*, et al.* Partner-delivered HIV self-test kits with and without financial incentives in antenatal care and index patients with HIV in Malawi: a three-arm, cluster-randomised controlled trial. *Lancet Global Health* 2021;**9**:e977-e88. <https://doi.org/10.1016/S2214-109X(21)00175-3>

10. Clark JL, Segura ER, Oldenburg CE, Salvatierra HJ, Rios J, Perez-Brumer AG*, et al.* Traditional and Web-Based Technologies to Improve Partner Notification Following Syphilis Diagnosis Among Men Who Have Sex With Men in Lima, Peru: Pilot Randomized Controlled Trial. *J Med Internet Res* 2018;**20**:e232. <https://doi.org/10.2196/jmir.9821>

11. Culbert GJ, Levy JA, Steffen AD, Waluyo A, Earnshaw VA, Rahadi A. Findings from a prison-based model of HIV assisted partner notification in Indonesia. *Journal of the International AIDS Society* 2023;**26**. <https://doi.org/10.1002/jia2.26132>

12. Dibia CC, Nwaokoro P, Akpan U, Toyo O, Cartier S, Sanwo O*, et al.* Innovations in Providing HIV Index Testing Services: A Retrospective Evaluation of Partner Elicitation Models in Southern Nigeria. *Glob Health Sci Pract* 2024;**12**. <https://doi.org/10.9745/GHSP-D-24-00013>

13. England DO, Currie MJ, Bowden FJ. An audit of contact tracing for cases of chlamydia in the Australian Capital Territory. *Sexual health* 2005;**2**:255-8. <https://doi.org/10.1071/sh05021>

14. Estcourt C, Sutcliffe L, Cassell J, Mercer CH, Copas A, James L*, et al.* Can we improve partner notification rates through expedited partner therapy in the UK? Findings from an exploratory trial of Accelerated Partner Therapy (APT). *Sexually transmitted infections* 2012;**88**:21-6. <https://doi.org/10.1136/sti.2010.047258>

15. Estcourt CS, Sutcliffe LJ, Copas A, Mercer CH, Roberts TE, Jackson LJ*, et al.* Developing and testing accelerated partner therapy for partner notification for people with genital Chlamydia trachomatis diagnosed in primary care: a pilot randomised controlled trial. *Sexually Transmitted Infections* 2015;**91**:548-54.

16. Estcourt C, Sutcliffe L, Mercer CH, Copas A, Saunders J, Roberts TE*, et al.* The Ballseye programme: a mixed-methods programme of research in traditional sexual health and alternative community settings to improve the sexual health of men in the UK. *NIHR Journals Library* 2016; 10.3310/pgfar04200. <https://doi.org/10.3310/pgfar04200>

17. Estcourt CS, Stirrup O, Copas A, Low N, Mapp F, Saunders J*, et al.* Accelerated partner therapy contact tracing for people with chlamydia (LUSTRUM): a crossover cluster-randomised controlled trial. *Lancet Public Health* 2022;**7**:e853-e65. <https://doi.org/10.1016/S2468-2667(22)00204-3>

18. Estcourt CS, Mapp F, Woode Owusu M, Low N, Flowers P, Copas A*, et al.* Improving sexual health through partner notification: the LUSTRUM mixed-methods research Programme including RCT of accelerated partner therapy. *National Institute for Health and Care Research* 2024; 10.3310/TRQW3886. <https://doi.org/10.3310/TRQW3886>

19. Faxelid E, Tembo G, Ndulo J, Krantz I. Individual counseling of patients with sexually transmitted diseases. A way to improve partner notification in a Zambian setting? *Sex Transm Dis* 1996;**23**:289-92.

20. Fetzer T, Graeber T. Measuring the scientific effectiveness of contact tracing: Evidence from a natural experiment. *Proc Natl Acad Sci U S A* 2021;**118**. <https://doi.org/10.1073/pnas.2100814118>

21. Findlater L, Pierotti L, Turner C, Wensley A, Chen C, Seaman S*, et al.* Evaluating the impact on health outcomes of an event that resulted in a delay in contact tracing of COVID-19 cases in England, September 2020: an observational study. *BMJ open* 2023;**13**:e064982. <https://doi.org/10.1136/bmjopen-2022-064982>

22. Hanrahan CF, Nonyane BAS, Mmolawa L, West NS, Siwelana T, Lebina L*, et al.* Contact tracing versus facility-based screening for active TB case finding in rural South Africa: A pragmatic cluster-randomized trial (Kharitode TB). *PLoS medicine* 2019;**16**:e1002796. <https://doi.org/10.1371/journal.pmed.1002796>

23. Hanrahan CF, Nonyane BAS, Lebina L, Mmolawa L, Siwelana T, West NS*, et al.* Household- Versus Incentive-Based Contact Investigation for Tuberculosis in Rural South Africa: A Cluster-Randomized Trial. *Clin Infect Dis* 2023;**76**:1164-72. <https://doi.org/10.1093/cid/ciac920>

24. Heumann CL, Katz DA, Dombrowski JC, Bennett AB, Manhart LE, Golden MR. Comparison of In-Person Versus Telephone Interviews for Early Syphilis and Human Immunodeficiency Virus Partner Services in King County, Washington (2010-2014). *Sex Transm Dis* 2017;**44**:249-54. <https://doi.org/10.1097/OLQ.0000000000000583>

25. Hu Q-H, Qian H-Z, Li J-M, Leuba SI, Chu Z-X, Turner D*, et al.* Assisted Partner Notification and Uptake of HIV Testing among Men Who Have Sex with Men: A Randomized Controlled Trial in China. *Lancet Reg Health West Pac* 2021;**12**:100171. <https://doi.org/10.1016/j.lanwpc.2021.100171>

26. Huang Y-W, Tsao C-Y, Chen W-W, Yen P-S, Lee J-J, Huang S-S*, et al.* Enhanced directly-observed treatment short-course for tuberculosis control program in mountain areas of Taiwan. *J Infect Dev Ctries* 2019;**13**:123-9. <https://doi.org/10.3855/jidc.10219>

27. Jerene D, Assefa D, Tesfaye K, Bayu S, Seid S, Aberra F*, et al.* Effectiveness of women-led community interventions in improving tuberculosis preventive treatment in children: Results from a comparative, before-after study in Ethiopia. *BMJ Open* 2022;**12**:e062298. <https://doi.org/10.1136/bmjopen-2022-062298>

28. Jose B, Manhica I, Jones J, Mutaquiha C, Zindoga P, Eduardo I*, et al.* Using community health workers for facility and community based TB case finding: An evaluation in central Mozambique. *PloS one* 2020;**15**:e0236262. <https://doi.org/10.1371/journal.pone.0236262>

29. Joshi B, Chinnakali P, Shrestha A, Das M, Kumar AMV, Pant R*, et al.* Impact of intensified case-finding strategies on childhood TB case registration in Nepal. *Public health action* 2015;**5**:93-8. <https://doi.org/10.5588/pha.15.0004>

30. Kagujje M, Nyangu S, Maimbolwa MM, Shuma B, Mutti L, Somwe P*, et al.* Strategies to increase childhood tuberculosis case detection at the primary health care level: Lessons from an active case finding study in Zambia. *PloS one* 2023;**18**:e0288643. <https://doi.org/10.1371/journal.pone.0288643>

31. Kaswaswa K, MacPherson P, Kumwenda M, Mpunga J, Thindwa D, Nliwasa M*, et al.* Effect of patient-delivered household contact tracing and prevention for tuberculosis: A household cluster-randomised trial in Malawi. *PloS one* 2022;**17**:e0269219. <https://doi.org/10.1371/journal.pone.0269219>

32. Katz BP, Danos CS, Quinn TS, Caine V, Jones RB. Efficiency and cost-effectiveness of field follow-up for patients with Chlamydia trachomatis infection in a sexually transmitted diseases clinic. *Sex Transm Dis* 1988;**15**:11-6. <https://doi.org/10.1097/00007435-198801000-00003>

33. Kerani RP, Fleming M, DeYoung B, Golden MR. A randomized, controlled trial of inSPOT and patient-delivered partner therapy for gonorrhea and chlamydial infection among men who have sex with men. *Sex Transm Dis* 2011;**38**:941-6. <https://doi.org/10.1097/OLQ.0b013e318223fcbc>

34. Ketema L, Dememew ZG, Assefa D, Gudina T, Kassa A, Letta T*, et al.* Evaluating the integration of tuberculosis screening and contact investigation in tuberculosis clinics in Ethiopia: A mixed method study. *PloS one* 2020;**15**:e0241977. <https://doi.org/10.1371/journal.pone.0241977>

35. Khatana GH, Haq I, Khan SMS. Effectiveness, acceptance and feasibility of home-based intervention model for tuberculosis contact tracing in Kashmir. *J Clin Tuberc Other Mycobact Dis* 2019;**14**:19-25. <https://doi.org/10.1016/j.jctube.2019.01.001>

36. Kissinger P, Mohammed H, Richardson-Alston G, Leichliter JS, Taylor SN, Martin DH*, et al.* Patient-delivered partner treatment for male urethritis: a randomized, controlled trial. *Clin Infect Dis* 2005;**41**:623-9. <https://doi.org/10.1086/432476>

37. Kissinger P, Schmidt N, Mohammed H, Leichliter JS, Gift TL, Meadors B*, et al.* Patient-delivered partner treatment for Trichomonas vaginalis infection: A randomized controlled trial. *Sex Transm Dis* 2006;**33**:445-50. <https://doi.org/10.1097/01.olq.0000204511.84485.4c>

38. Landis SE, Schoenbach VJ, Weber DJ, Mittal M, Krishan B, Lewis K*, et al.* Results of a randomized trial of partner notification in cases of HIV infection in North Carolina. *N Engl J Med* 1992;**326**:101-6. <https://doi.org/10.1056/NEJM199201093260205>

39. Low N, McCarthy A, Roberts TE, Huengsberg M, Sanford E, Sterne JAC*, et al.* Partner notification of chlamydia infection in primary care: randomised controlled trial and analysis of resource use. *BMJ (Clinical research ed)* 2006;**332**:14-9. <https://doi.org/10.1136/bmj.38678.405370.7C>

40. Lugada E, Levin J, Abang B, Mermin J, Mugalanzi E, Namara G*, et al.* Comparison of home and clinic-based HIV testing among household members of persons taking antiretroviral therapy in Uganda: Results from a randomized trial. *J Acquir Immune Defic Syndr* 2010;**55**:245-52. <https://doi.org/10.1097/QAI.0b013e3181e9e069>

41. Lukac CD, Consolacion T, Ryan V, Cumming E, Mercado J, Ford G*, et al.* Population-Level Outcomes of Partner Notification Among Gay, Bisexual, and Other Men Who Report Sex With Men Diagnosed With Infectious Syphilis in British Columbia, Canada. *Sex Transm Dis* 2021;**48**:901-8. <https://doi.org/10.1097/OLQ.0000000000001477>

42. Luo M, Hann K, Zhang G, Pan X, Ma Q, Jiang J*, et al.* HIV testing uptake and yield among sexual partners of HIV-positive men who have sex with men in Zhejiang Province, China, 2014-2016: A cross-sectional pilot study of a choice-based partner tracing and testing package. *PloS one* 2020;**15**:e0232268. <https://doi.org/10.1371/journal.pone.0232268>

43. Malave MC, Shah D, Sackoff JE, Rubin S, Begier EM. Human immunodeficiency virus partner elicitation and notification in new york city: public health does it better. *Sex Transm Dis* 2008;**35**:869-76. <https://doi.org/10.1097/OLQ.0b013e31817d2f82>

44. Malheiro R, Figueiredo AL, Magalhaes JP, Teixeira P, Moita I, Moutinho MC*, et al.* Effectiveness of contact tracing and quarantine on reducing COVID-19 transmission: a retrospective cohort study. *Public health* 2020;**189**:54-9. <https://doi.org/10.1016/j.puhe.2020.09.012>

45. Martinson NA, Lebina L, Webb EL, Ratsela A, Varavia E, Kinghorn A*, et al.* Household Contact Tracing With Intensified Tuberculosis and Human Immunodeficiency Virus Screening in South Africa: A Cluster-Randomized Trial. *Clin Infect Dis* 2022;**75**:849-56. <https://doi.org/10.1093/cid/ciab1047>

46. Mathews C, Lombard C, Kalichman M, Dewing S, Banas E, Dumile S*, et al.* Effects of enhanced STI partner notification counselling and provider-assisted partner services on partner referral and the incidence of STI diagnosis in Cape Town, South Africa: randomised controlled trial. *Sexually transmitted infections* 2021;**97**:38-44. <https://doi.org/10.1136/sextrans-2020-054499>

47. Morishita F, Eang MT, Nishikiori N, Yadav R-P. Increased Case Notification through Active Case Finding of Tuberculosis among Household and Neighbourhood Contacts in Cambodia. *PloS one* 2016;**11**:e0150405. <https://doi.org/10.1371/journal.pone.0150405>

48. Oh MK, Boker JR, Genuardi FJ, Cloud GA, Reynolds J, Hodgens JB. Sexual contact tracing outcome in adolescent chlamydial and gonococcal cervicitis cases. *J Adolesc Health* 1996;**18**:4-9. <https://doi.org/10.1016/1054-139X(95)00109-6>

49. Østergaard L, Andersen B, Møller JK, Olesen F, Worm AM. Managing partners of people diagnosed with Chlamydia trachomatis: A comparison of two partner testing methods. *Sexually Transmitted Infections* 2003;**79**:358-62. <https://doi.org/10.1136/sti.79.5.358>

50. Parkes-Ratanshi R, Mbazira Kimeze J, Nakku-Joloba E, Hamill MM, Namawejje M, Kiragga A*, et al.* Low male partner attendance after syphilis screening in pregnant women leads to worse birth outcomes: the Syphilis Treatment of Partners (STOP) randomised control trial. *Sexual health* 2020;**17**:214-22. <https://doi.org/10.1071/SH19092>

51. Peterman TA, Toomey KE, Dicker LW, Zaidi AA, Wroten JE, Carolina J. Partner notification for syphilis: a randomized, controlled trial of three approaches. *Sex Transm Dis* 1997;**24**:511-8. <https://doi.org/10.1097/00007435-199710000-00003>

52. Potterat JJ, Rothenberg R. The case finding effectiveness of a self referral system for gonorrhea: a preliminary report. *Am J Public Health* 1977;**67**:174-6. <https://doi.org/10.2105/AJPH.67.2.174>

53. Raymenants J, Geenen C, Thibaut J, Nelissen K, Gorissen S, Andre E. Empirical evidence on the efficiency of backward contact tracing in COVID-19. *Nat Commun* 2022;**13**:4750. <https://doi.org/10.1038/s41467-022-32531-6>

54. Schwebke JR, Desmond RA. A randomized controlled trial of partner notification methods for prevention of trichomoniasis in women. *Sex Transm Dis* 2010;**37**:392-6. <https://doi.org/10.1097/OLQ.0b013e3181dd1691>

55. Shah L, Rojas Pena M, Mori O, Zamudio C, Kaufman JS, Otero L*, et al.* A pragmatic stepped-wedge cluster randomized trial to evaluate the effectiveness and cost-effectiveness of active case finding for household contacts within a routine tuberculosis program, San Juan de Lurigancho, Lima, Peru. *Int J Infect Dis* 2020;**100**:95-103. <https://doi.org/10.1016/j.ijid.2020.09.034>

56. Tchakounte Youngui B, Mambo A, Machekano R, Kana R, Epee E, Tenkeu SZ*, et al.* Improving COVID-19 contact tracing and testing of exposed individuals in Cameroon using digital health technology: a cluster randomised trial. *EClinicalMedicine* 2024;**74**:102730. <https://doi.org/10.1016/j.eclinm.2024.102730>

57. Wilson TE, Hogben M, Malka ES, Liddon N, McCormack WM, Rubin SR*, et al.* A randomized controlled trial for reducing risks for sexually transmitted infections through enhanced patient-based partner notification. *Am J Public Health* 2009;**99 Suppl 1**:S104-10. <https://doi.org/10.2105/AJPH.2007.112128>

58. Adejugbagbe AM, Fatiregun AA, Dosumu MO, Itse O, Akanbiemu FA, Fagbemi S*, et al.* Lessons learnt from decentralization of COVID-19 response in a southwest state of Nigeria. *Glob Biosecurity* 2022;**4**. <https://doi.org/10.31646/gbio.158>

59. Beebeejaun K, Amin-Chowdhury Z, Letley L, Kara E, Mahange B, Harrington K*, et al.* Impact of a nurse-led enhanced monitoring, management and contact tracing intervention for chronic hepatitis B in England, 2015-2017. *Journal of viral hepatitis* 2021;**28**:72-9. <https://doi.org/10.1111/jvh.13403>

60. Djuric O, Larosa E, Cassinadri M, Cilloni S, Bisaccia E, Pepe D*, et al.* Effect of an enhanced public health contact tracing intervention on the secondary transmission of SARS-CoV-2 in educational settings: The four-way decomposition analysis. *eLife* 2024;**13**. <https://doi.org/10.7554/eLife.85802>

61. Duarte R, Neto M, Carvalho A, Barros H. Improving tuberculosis contact tracing: the role of evaluations in the home and workplace. *Int J Tuberc Lung Dis* 2012;**16**:55-9. <https://doi.org/10.5588/ijtld.10.0511>

62. Dzhangaziev B, Kulzhabaeva A, Truzyan N, Zhoroev A, Otorbaeva D, Temirbekov S*, et al.* New approach for tuberculosis contact tracing implemented in the two regions of Kyrgyz Republic during 2017-2018. *J Infect Dev Ctries* 2020;**14**:109S-15S. <https://doi.org/10.3855/jidc.11978>

63. Edmiston N, Merritt T, Ooi C. Make contact: a comparative study of contact tracing strategies. *International journal of STD & AIDS* 2010;**21**:431-4. <https://doi.org/10.1258/ijsa.2010.010118>

64. Engelgau MM, Woernle CH, Rolfs RT, Greenspan JR, O'Cain M, Gorsky RD. Control of epidemic early syphilis: the results of an intervention campaign using social networks. *Sex Transm Dis* 1995;**22**:203-9.

65. Fatima R, Qadeer E, Yaqoob A, Haq MU, Majumdar SS, Shewade HD*, et al.* Extending 'Contact Tracing' into the Community within a 50-Metre Radius of an Index Tuberculosis Patient Using Xpert MTB/RIF in Urban, Pakistan: Did It Increase Case Detection? *PloS one* 2016;**11**:e0165813. <https://doi.org/10.1371/journal.pone.0165813>

66. Fiumara NJ, Segal J, Jolly J. Venereal disease contact investigation; a combined military-civilian program. *Public health reports (Washington, DC : 1896)* 1953;**68**:289-94.

67. Fu X, Qi J, Hu Y, Pan X, Li Y, Liu H*, et al.* Partner notification in cooperation with community-based organizations among HIV-positive men who have sex with men in two Chinese cities. *International journal of STD & AIDS* 2016;**27**:821-31. <https://doi.org/10.1177/0956462416648827>

68. Grande M, Mawandia S, Bakae O, Tau L, Mogomotsi GP, Ngombo M*, et al.* Intensified Assisted Partner Notification Implementation in Botswana Increased Partner Identification but Not HIV Case-Finding: Findings Highlight the Need for Improved Data Monitoring. *J Acquir Immune Defic Syndr* 2021;**87**:951-8. <https://doi.org/10.1097/QAI.0000000000002673>

69. Jones AT, Craig-Kuhn MC, Schmidt N, Gomes G, Scott G, Jr., Watson S*, et al.* Adapting Index/Partner Services for the Treatment of Chlamydia Among Young African American Men in a Community Screening Program. *Sex Transm Dis* 2021;**48**:323-8. <https://doi.org/10.1097/OLQ.0000000000001325>

70. Kantor R, Steingrimsson J, Fulton J, Novitsky V, Howison M, Gillani F*, et al.* Prospective Evaluation of Routine Statewide Integration of Molecular Epidemiology and Contact Tracing to Disrupt Human Immunodeficiency Virus Transmission. *Open Forum Infect Dis* 2024;**11**:ofae599. <https://doi.org/10.1093/ofid/ofae599>

71. Kingbo M-HKA, Isaakidis P, Lasry A, Takarinda KC, Manzi M, Pringle J*, et al.* Partner Notification Approaches for Sex Partners and Children of Human Immunodeficiency Virus Index Cases in Cote d'Ivoire. *Sex Transm Dis* 2020;**47**:450-7. <https://doi.org/10.1097/OLQ.0000000000001180>

72. Learner ER, Schlanger K, Mauk K, Pham CD, Mukai R, Mulleavey L*, et al.* Outcomes of Traditional and Enhanced Gonorrhea Partner Services in the Strengthening the US Response to Resistant Gonorrhea Project, 2017 to 2019. *Sex Transm Dis* 2021;**48**:S124-S30. <https://doi.org/10.1097/OLQ.0000000000001527>

73. Mahachi N, Muchedzi A, Tafuma TA, Mawora P, Kariuki L, Semo B-W*, et al.* Sustained high HIV case-finding through index testing and partner notification services: experiences from three provinces in Zimbabwe. *Journal of the International AIDS Society* 2019;**22 Suppl 3**:e25321. <https://doi.org/10.1002/jia2.25321>

74. Martinez-Camprecios J, Gil E, Aixut S, Moreno M, Zacarias A, Nindia A*, et al.* Tuberculosis contact tracing, Angola. *Bulletin of the World Health Organization* 2024;**102**:196-203. <https://doi.org/10.2471/BLT.23.290068>

75. Ospina JE, Orcau A, Millet J-P, Sanchez F, Casals M, Cayla JA. Community health workers improve contact tracing among immigrants with tuberculosis in Barcelona. *BMC public health* 2012;**12**:158. <https://doi.org/10.1186/1471-2458-12-158>

76. Osterlund A, Persson T, Persson I, Lysen M, Herrmann B. Improved contact tracing of Chlamydia trachomatis in a Swedish county--is genotyping worthwhile? *International journal of STD & AIDS* 2005;**16**:9-13. <https://doi.org/10.1258/0956462052932809>

77. Qader G, Hamim A, Sayedi M, Rashidi M, Manzoor L, Seddiq MK*, et al.* Addressing tuberculosis control in fragile states: Urban DOTS experience in Kabul, Afghanistan, 2009-2015. *PloS one* 2017;**12**:e0178053. <https://doi.org/10.1371/journal.pone.0178053>

78. Rudy ET, Aynalem G, Cross J, Ramirez F, Bolan RK, Kerndt PR. Community-embedded disease intervention specialist program for syphilis partner notification in a clinic serving men who have sex with men. *Sex Transm Dis* 2012;**39**:701-5. <https://doi.org/10.1097/OLQ.0b013e3182593b51>

79. Sanaie A, Mergenthaler C, Nasrat A, Seddiq MK, Mahmoodi SD, Stevens RH*, et al.* An Evaluation of Passive and Active Approaches to Improve Tuberculosis Notifications in Afghanistan. *PloS one* 2016;**11**:e0163813. <https://doi.org/10.1371/journal.pone.0163813>

80. Schleihauf E, Leonard E, Phillips C, Hatchette T, Haldane D, Arnason T*, et al.* Increase in Gonorrhea Incidence Associated With Enhanced Partner Notification Strategy. *Sex Transm Dis* 2019;**46**:706-12. <https://doi.org/10.1097/OLQ.0000000000001060>

81. Sulis G, Combary A, Getahun H, Gnanou S, Giorgetti PF, Konseimbo A*, et al.* Implementation of tuberculosis prevention for exposed children, burkina faso. *Bulletin of the World Health Organization* 2018;**96**:386-92. <https://doi.org/10.2471/BLT.17.201343>

82. Taylor MM, Mickey T, Winscott M, James H, Kenney K, England B. Improving partner services by embedding disease intervention specialists in HIV-clinics. *Sex Transm Dis* 2010;**37**:767-70. <https://doi.org/10.1097/OLQ.0b013e3181e65e8b>

83. Tembo TA, Kim MH, Simon KR, Ahmed S, Beyene T, Wetzel E*, et al.* Enhancing an HIV index case testing passive referral model through a behavioural skills-building training for healthcare providers: a pre-/post-assessment in Mangochi District, Malawi. *Journal of the International AIDS Society* 2019;**22**:e25292. <https://doi.org/10.1002/jia2.25292>

84. Tembo TA, Simon KR, Kim MH, Chikoti C, Huffstetler HE, Ahmed S*, et al.* Pilot-Testing a Blended Learning Package for Health Care Workers to Improve Index Testing Services in Southern Malawi: An Implementation Science Study. *J Acquir Immune Defic Syndr* 2021;**88**:470-6. <https://doi.org/10.1097/QAI.0000000000002796>

85. Udeagu C-CN, Shah D, Shepard CW, Bocour A, Guiterrez R, Begier EM. Impact of a New York City Health Department initiative to expand HIV partner services outside STD clinics. *Public health reports (Washington, DC : 1974)* 2012;**127**:107-14.

86. van de Berg S, Erkens C, Mulder C. Tuberculosis contact investigation following the stone-in-the-pond principle in the Netherlands - Did adjusted guidelines improve efficiency? *Euro Surveill* 2021;**26**. <https://doi.org/10.2807/1560-7917.ES.2021.26.45.2001828>

87. Volkmann T, Okelloh D, Agaya J, Cain K, Ooko B, Malika T*, et al.* Pilot implementation of a contact tracing intervention for tuberculosis case detection in Kisumu County, Kenya. *Public health action* 2016;**6**:217-9. <https://doi.org/10.5588/pha.16.0032>

88. Zachariah R, Spielmann MP, Harries AD, Gomani P, Graham SM, Bakali E*, et al.* Passive versus active tuberculosis case finding and isoniazid preventive therapy among household contacts in a rural district of Malawi. *Int J Tuberc Lung Dis* 2003;**7**:1033-9.
