# Supplementary material for: The Effectiveness of Contact Tracing to Reduce Transmission of Infectious Diseases During Epidemic or Pandemic Response: Rapid Systematic Review
Source: JMIR Public Health Surveill. 2026 Mar 31;12:e84805. doi: 10.2196/84805 (PMC13080299; doi:10.2196/84805)
Supplement: Multimedia Appendix 7 [file publichealth_v12i1e84805_app7.docx]

## Table S1. Overview of study characteristics, intervention components and findings of studies to improve contact tracing in tuberculosis and COVID-19

| **Author, date; design; quality** | **Disease; country; setting** | **Intervention** | | **Comparator** | **Outcomes** | | | |
| --- | --- | --- | --- | --- | --- | --- | --- | --- |
|  |  | **Category** | **Key components** |  | **Disease incidence or prevalence** | **Case detection** | **Treatment rates** | **Others** |
| Bai, 2008;^1^  OCC;  WEAK | TB; China; Community | PICT | Referral slip  Incentive  Active follow-up  Home visits  DOTS | DOTS  (No description of case finding)  (Usual care) | **Reduced**  (2 outcomes) |  |  |  |
| Cavalcante, 2010;^2^  cRCT;  WEAK | TB; Brazil; Community | PICT | Intensive screening  Active follow-up  Home visits  Prophylaxis | DOTS with PR  (Usual care) | **Reduced**  (1 outcome)  No difference (1 outcome) |  |  |  |
| Hanrahan, 2019;^3^  cRCT;  STRONG | TB; South Africa; Primary care | PICT/HI | Incentive  Home visits | Facility screening  (Usual care) |  |  | No difference (1 outcome) |  |
| Hanrahan, 2023,^4^  RCT,  WEAK | TB; South Africa; Primary care/ community | PICT | Incentive | Home visits  Mandatory testing  (Usual care) | No difference (1 outcome) |  | No difference (1 outcome) |  |
| Huang, 2019;^5^  CBA;  WEAK | TB; Taiwan; NR | PICT | Enhanced DOTS  Indigenous case manager  Screening of close contacts (and frequent visitors) | DOTS  Case manager  Active case finding  (Usual care) |  |  | **Increased**  (1 outcome) |  |
| Jerene, 2022;^6^  CBA;  WEAK | TB; Ethiopia; Community | HI | Female team of Iddirs*  Home visit  Incentive  Referral slip  Education  Active follow-up | Passive case finding  (Usual care) |  |  | **Increased**  (1 outcome) |  |
| Jose, 2020;^7^  CBA;  WEAK | TB; Mozambique; Community | PICT/HI | Active follow-up  Education  Home visits | Passive case finding  (Usual care) | **Reduced**^+^  (2 outcomes) |  |  |  |
| Joshi, 2015;^8^  CBA;  MODERATE | TB; Nepal; Community | PICT | Intensive childhood case finding  Household contact screening  Mobile screening units  Screening at school  DOTS | DOTS  (no description of case finding)  (Usual care) |  | **Increased**  (1 outcome) |  |  |
| Kagujje, 2023,^9^  cITS,  WEAK | TB; Zambia; Public hospitals | PICT | Capacity building  Education  Fast track services  Home visits | Passive case finding  Child TB screening algorithms (Usual care) | **Reduced**^+^  (1 outcome) |  |  |  |
| Kaswaswa, 2022;^10^  cRCT;  MODERATE | TB; Malawi, Clinics & community | Usual care + Index case reporting (PR) | Screening and testing of household contacts by index patient | Passive case finding  Routine HIV testing and treatment linkage  (Usual care) |  | No difference (1 outcome) | No difference (2 outcomes) |  |
| Ketema, 2020;^11^  RCT;  WEAK | TB; Ethiopia; DOTS clinics | PICT | Evaluation at DOTS clinics  Enhanced childhood TB case finding  Prophylaxis offered to contacts | Non-integrated TB screening at DOTS clinics (Usual care) |  | No difference (1 outcome) | **Increased**  (1 outcome) |  |
| Khatana, 2019;^12^  Quasi RCT;  MODERATE | TB; India; Home/TB unit | HI | Weekly home visit on fixed day  Testing of household contacts | Passive case finding  (Usual care) |  | **Increased**  (1 outcome) |  |  |
| Martinson, 2022;^13^  cRCT;  STRONG | TB; South Africa; Community | HI | Home visits  Testing and screening  Treatment linkage support | PR  Referral letters for contacts (Usual care) | No difference (1 outcome) |  |  | Mortality: no difference  (1 outcome) |
| Morishita, 2016;^14^  Quasi cRCT;  MODERATE | TB; Cambodia; Community | PICT/HI | Home visits  10 nearest homes visited  Invitation to ACF if symptomatic | Passive case finding  (Usual care) | **Reduced**^+^  (1 outcome) |  |  |  |
| Shah, 2020;^15^  cRCT;  MODERATE | TB; Peru; Clinics | HI | Active monitoring  Home visits  Testing and referral | Passive case finding  PR  Passive monitoring  Self-report to clinic  (Usual care) | **Reduced** ^+^  (1 outcome) |  |  |  |
| Fetzer, 2021;^16^  cITS;  MODERATE | Covid-19; UK; Community | TECH | Delayed notification of close contacts due to error | National CT system  Phone and digital tracing (Usual care) | **Increased**  (3 outcomes) |  |  | Mortality: **Increased**  (1 outcome) |
| Findlater, 2023;^17^  cITS;  WEAK | Covid-19; UK; Community | TECH | Delayed notification of close contacts due to error | National CT system  Phone and digital tracing (Usual care) |  | No difference  (2 outcomes) |  | Mortality: No difference  (4 outcomes)  Hospital admission: **Increased**  (1 outcome)  No difference: (1 outcome) |
| Malheiro, 2020;^18^  OCC;  STRONG | Covid-19; Portugal; Community | PICT | Interview  Quarantine orders  Active follow-up | No CT  No quarantine |  | No difference  (2 outcomes) |  |  |
| Raymenants, 2022;^19^  OCC;  WEAK | Covid-19;  Belgium;  Community | PICT | Interview  Extended window | No CT |  | **Increased**  (1 outcome) |  |  |
|  |  | PICT | Interview  Standard window | No CT |  | **Increased**  (1 outcome) |  |  |
|  |  | PICT | Interview  Extended window | Interview  Standard window |  | **Increased**^+^  (1 outcome) |  |  |
| Tchakounte, 2024;^20^  cRCT;  Strong | Covid-19; Cameroon; Community | TECH | Phone application  Interview  Automated CT via SMS  Active follow-up of contacts | Manual listing of contacts (Usual care) |  | No difference  (2 outcomes) |  |  |
| ACF, active case finding; CBA, controlled before-and-after; CT, contact tracing; cITS, controlled ITS; cRCT, cluster RCT; DOTS, directly observed treatment (short-course); HI, household investigation; NR, not reported; OCC, observational study with concurrent control group; PICT, provider-initiated CT; PR, patient referral CT; PN, partner notification; PR, patient referral; RCT, randomised controlled trial; SMS, short messaging service; TB, tuberculosis; TECH, technology-based CT; UK, United Kingdom. *Iddirs, a group of local people who have formed a membership-based association and have voluntarily entered into an agreement to help each other during times of difficulty. | | | | | | | | |

## Table S2. Characteristics of interventions to improve contact tracing in tuberculosis and Covid-19

| **Author, date** | **Study arm name** | **Study arm summary** | **Duration** | **Setting** | **Who delivered** |
| --- | --- | --- | --- | --- | --- |
| *Tuberculosis studies* | | | | | |
| Bai, 2008^1^ | New strategic referral and tracing system | County TB dispensaries were responsible for diagnosis and treatment of TB patients. Key activities: 1) Training and health promotion. 2) Referral of TB suspects/patients: use of a referral slips and designated staff reporting at hospital/clinic; use of financial incentives. 3) Tracing of suspects/cases via disease control website. Case tracing carried out within a week using telephone calls, letters and home visits on any TB suspects/patients who did not attend clinic. Home visits were carried out by town and village doctors. Both smear-positive and smear-negative PTB cases received treatment free of charge and were managed under DOTS. | 12 months | Community (including hospitals/clinics and TB dispensaries) | TB dispensary staff / county staff ran training / hospital staff |
|  | Non-project area | Usual care (DOTS strategy without intensive referral and tracing). | 12 months | Community | NR |
| Cavalcante, 2010^2^ | DOTS-A (enhanced DOTS) | Enhanced routine DOTS with the addition of intensive screening of HHCs of TB cases for evidence of active TB or LTBI. ICs provided names of HHCs and prompted contacts to attend clinic for evaluation. Input from outreach worker if contact failed to attend. | 5 years | One of the three municipal health centres within the communities | Healthcare staff at health centres and outreach workers |
|  | DOTS | Following Brazil’s NTP guidelines, clinic staff informed TB ICs of the need for their HHCs to come to the clinic for evaluation. | 5 years | One of the three municipal health centres within the communities | Healthcare staff at health centre |
| Hanrahan, 2019^3^ | CT (split 50:50 incentive and household) | Incentive programme: index patients and their contacts were given monetary incentives if the contact presented to the clinic for screening, and the index patient received extra if a contact tested positive. Household programme: At the household, all contacts present were offered TB screening, and those contacts not already on TB treatment had sputum collected for testing. Up to two additional visits for comprehensiveness. | 18 months | Public primary care clinics | Study staff |
|  | Facility-based screening | SOC in South Africa (comprised initial symptom-based screening, escalating to sputum collection and Xpert MTB/RIF testing). | 18 months | Public primary care clinics | Study staff |
|  | Incentive-based contact investigation | Index patients and their contacts were given monetary incentives if the contact presented to the clinic for screening, and the index patient received extra if a contact tested positive. | 18 months | Public primary care clinics | Health facility staff |
| Hanrahan, 2023^4^ | Household CT | Study teams visited index patient household and administered a questionnaire covering sociodemographic items, clinical history and symptom screen to all eligible consenting household contacts. Sputum sample collected from all contacts, regardless of symptoms. | 18 months | Home-based | Study staff |
| Huang, 2019^5^ | E-DOTS (enhanced-DOTS) | Employed indigenous case managers for screening of close contacts, including HHCs and frequent visitors of ICs, as well as ensuring patients’ adherence to TB treatment. | NR | NR | Lay health advisors |
|  | DOTS | Case manager to ensure patient compliance; ACF. | NR | NR | Health workers |
| Jerene, 2022^6^ | Women-led community intervention | Team of women-only Iddir* members and volunteers were trained on basics of TB, focusing on U5C contact management and TPT. Home visits to ICs and screening of contacts with focus on U5C. All U5C received a referral slip. Follow up calls/visits/texts with focus on U5C. Linkage with community mobilisers and zonal project coordinators for data collection, monitoring and evaluation. Certificate of completion incentive. | ~12 months | Community (including home visits) | Community leaders (Iddirs*), community mobilisers, TB officers and community volunteers |
|  | SOC | National SOC (contact investigation rarely practiced at community level; Iddirs* do not participate in TB services) If children present at a health facility as a household contact of a person with TB, they are offered TPT if they are found not to have active disease. | ~12 months | Health centre / hospitals | Health extension workers, healthcare professionals |
| Jose, 2020^7^ | Community-based TB case-finding | 30 CHWs employed and trained across 5 intervention districts to carry out ACF through facility-based screening. TB sensitisation sessions on all individuals presenting to local health facilities, and active CT of people diagnosed and notified with TB through household visits. CHWs linked with facility TB supervisors and community leaders. CHWs also identified individuals known to be pre-treatment lost to follow up or lost to follow up and carried out community-based tracing to find and link these individuals to care. | 12 months | Community | CHWs |
|  | SOC (passive case finding) | PCF and testing. Control districts did not receive any TB ACF activity during the study. | 12 months | Primarily health centres | NA (self-report) |
| Joshi, 2015^8^ | Intensified childhood tuberculosis case finding | Intensified early TB case detection amongst children aged 0–14 years in 10 districts of Nepal. Several case finding screening activities including HHC screening, private and public mix services, mobile health chest camps, community home based care units, screening of children at school, safe motherhood health services. | 12 months | Community: 7/10 intervention districts (3 delayed and not included) | Program volunteers |
|  | Standard DOTS strategy | WHO recommended DOTS strategy. | 12 months | Community | NR |
| Kagujje, 2023^9^ | Multicomponent TB detection strategy | Intervention involved: (1) Capacity development on childhood TB among HCWs (2) TB awareness raising and demand creation (3) setting up fast track TB evaluation services (4) strengthening of household CT (5) improving access to screening and diagnosis. Strengthening household CT: HCWs conducted CT of adult TB patients and reverse CT of children diagnosed with TB. Home visits to provide education and invite all contacts for TB screening or completed symptom screening at the home. Home visits replaced with phone calls for symptom-based screening during Covid-19. | 19 months | Hospital | CHW |
|  | SOC | Two control sites provided standard of care services for childhood TB defined by the Zambian Ministry of Health. No new interventions or strategies were implemented. Childhood TB case finding was mainly passive (e.g., testing only among symptomatic children brought for evaluation) via the outpatient department. Childhood TB screening algorithms were available in the control sites; however, they may not have been routinely used by healthcare providers. | 19 months | Outpatient clinic | Healthcare worker |
| Kaswaswa 2022^10^ | PACTS | SOC + IC delivered TB screening of HHCs and testing of symptomatic HHCs. ICs asked to return to the study clinics within 28 days, along with completed HHC screening cards and sputum samples and all U5C. | 3 months | Clinics and community | Research assistants |
|  | SOC | Encouraged all household members to report to the health facility for TB screening and HIV testing in accordance with national guidelines. Also advised to return to the facility after 28 days with their children. HIV testing and linkage to ART offered to ICs through the routine system. | 3 months | Clinics and community | Research assistants |
| Ketema, 2020^11^ | Contact investigation in TB DOTS clinic | Enhanced childhood TB case finding in the TB DOTS unit clinics through contact investigation. TB screening and diagnosis, and CT were based on the existing national guidelines and algorithms. All U5C contacts of PTB ICs identified at the TB DOTS clinics underwent evaluation for TB and all children with screen negative or non-presumed TB case were eligible for prophylaxis and offered IPT. | Ranged between 4 and 12 months | TB DOTS clinic | Healthcare workers |
|  | Control | Baseline assessment. | Ranged between 4 and 12 months | IMNCI and DOTS clinics | Healthcare workers |
| Khatana, 2019^12^ | Interventional group - home based interventional model (ACF) | Home visited of IC on fixed weekly contact evaluation day. Spot sputum specimen collected from symptomatic contacts, second sputum specimen to be submitted by any family member. | 6 months | Home/TB unit | Medical Officer of concerned TB Unit |
|  | Controlled group (SOC) | SOC (CT under Revised National Tuberculosis Control Programme (PCF)). | 6 months | TB unit | Health staff/ TB Unit staff |
| Martinson, 2022^13^ | Home tracing and intensive HIV/TB screening | House visits to TB ICs conducted within 14 days of enrolment (maximum 3 attempts), for screening and testing of household members for TB and HIV. Intervention households visited around 3 months to support treatment linkage. | 14 days maximum for visits, also visited 3 months later | Home-based | Research fieldworkers |
|  | SOC (clinic referral letters) | Index TB patients were given referral letters household members, recommending screening for TB and HIV at clinic. | NR | Health facility | Recruiting team at health facility |
| Morishita, 2016^14^ | ACF (+ PCF) | House visits to TB ICs registered for treatment during the preceding 2 years for screening of HHCs. 10 nearest households (or next nearest within same village) visited and screened to identify neighbourhood contacts. All HHCs and symptomatic neighbourhood contacts invited to ACF session at nearest health centres where contacts were re-screened and tested for TB symptoms. All detected patients-initiated treatment immediately (within a few days in most cases) and managed by routine health services. | Two 12-month periods | Community | Community volunteers |
|  | PCF | Routine PCF. | 10 months | Health centres | Health workers |
| Shah, 2020^15^ | ACF | Up to three household visits to TB ICs ≥16 years to screen household members for symptoms of active TB (cough >14 days). Testing of symptomatic contacts ≥15 years old and referral to the NTP centre for evaluation. HHCs <15 years old referred for evaluation regardless of symptoms as per TB program guidelines. In the health centres, NTP staff followed Ministry guidelines for evaluating HHCs and initiating treatment for active TB or latent TB infection as indicated. | 20 months | Community | Healthcare staff |
|  | PCF | TB cases presenting to NTP clinics were asked to name all HHCs and encouraged to tell household members ≥15 years with cough >14 days to self-report to the clinic for evaluation. TB cases were diagnosed using existing NTP guidelines throughout the trial. | 20 months | Clinic | Healthcare staff |
| *Covid-19 studies* | | | | | |
|  | Delayed CT due to the Excel error | There was a “technical issue” discovered in the night of October 2nd - 3rd, which caused a total of 15,841 positive cases (20% of all cases at the time) to be missed in both the officially reported figures and the case data that was transferred to the national CT system. Information had accidentally been truncated from Excel spreadsheets. The original reporting dates of the missed cases were September 25th-October 2nd. The data glitch did not affect the dissemination of test results to people who tested positive, but close recent contacts experienced a delay in CT. | 1 week | NA | NA |
| Fetzer, 2021^16^ | National contract tracing program | SOC (from national CT system: NHS Test and Trace. All PCR-positive cases in England were identified and contacted by phone, digital tools and through partnerships with local authorities).** | 1 week | NA | NA |
|  | Delayed CT | Between 25^th^ September-2^nd^ October 2020, some test results from community testing sites failed to import from the laboratory surveillance system to the CTAS. The event did not affect test result notifications or affect the results of any specific testing sites, geographical areas or population groups. However, the delay in the upload of case records to CTAS resulted in the contacts of these cases experiencing a delay in CT. | 1 week | Community testing sites | NA |
| Findlater, 2023^17^ | National contract tracing program | SOC (from national CT system: NHS Test and Trace. All PCR tested positive cases in England were identified and contacted by phone, digital tools and through partnerships with local authorities). | 1 week | Community testing sites | NA |
|  | CT and quarantine | CT involved a detailed interview with the Covid-19 patient or their caregiver, capturing all household, family, work/school, and social contacts who have had contact with a confirmed case (within 2 days before symptom onset and 14 days afterward). Close contacts were placed under mandatory quarantine and assessed once daily, by telephone, for Covid-19 symptoms. Follow-ups of close contacts ended 14 days after the last exposure or if the contact was diagnosed with Covid-19. Work-related contacts of healthcare and nursing home staff were excluded, as they were managed elsewhere. Close contacts with Covid-19 symptoms were transferred directly to a healthcare facility for further evaluation and testing. | 14 days | Community | Local public health authorities |
| Malheiro, 2020^18^ | No CT | All Covid-19 confirmed cases that were not subject to CT nor to quarantine measures preceding the diagnosis were controls. | NA | Community | NA |
|  | Extended window (backward) CT | An extended tracing window was employed (i.e. their last close interaction with the IC was 3-7 days before onset or test). The backward traced group consisted of 788 additional contacts. | 7 days | Community | NA |
|  | Standard-window CT | The standard tracing window group, which would have been identified through standard practice, consisted of 1608 individuals in close contact with the IC in the period from 2 days before onset or test until the CT interview. | 2 days | Community | Government contact tracers |
| Raymenants, 2022^19^ | No CT | Students who attended our test centre for the first time with self-reported symptoms suggestive of Covid-19 as the main reason for their test were control. | 0 days | Community | NA |
|  | Digitalised CT | Following app registration and SARS-CoV-2 testing, the contacts of those testing positive was transmitted to the CT unit of the health district digitally. Individuals tested positive were referred to the Covid-19 prevention, care and treatment focal person (Covid-19 focal person) of each facility, who has administrator access to the “Mamal PRO” app. They carried out a clinical interview, involving updating client-entered demographic and clinical information and completing other sections (e.g. link with the IC and any additional contacts). Contract tracing was activated, automatically sending the list of contacts with their details to the CT unit and sending a text message to contacts to inform them about their recent exposure to SARS-CoV-2 and encourage them to get tested rapidly. District HCWs who call contacts had contact tracer app access, allowing them to see the lists of contacts in their health district and to enter outcome data of each CT attempt. All follow-up information were recorded in real time and monitored by supervisors at the health district and regional level. HCWs involved in the CT activities were trained use of the app module. | 23 weeks | Health facility/community | Health workers |
| Tchakounte, 2024^20^ | Manual CT | In the control arm, ICs still registered on the app and used it during the Covid-19 testing process. However, the CT module of the app was not available, and therefore CT involved manual listing of contacts by the HCW after an index case was diagnosed using national paper-based contact line listing forms. Once completed, the physical forms were then transferred to the district unit and manual documentation (using paper-based tracking forms and registers) of each contact was followed up. They were referred to the COVID-19 focal person for further care and listing recent contacts using the national paper-based contact line listing form. These contact line listing forms were transferred daily to the district unit responsible for CT. The HCW in charge of CT documented the follow up and the outcome of the CT. | 23 weeks | Health facility/ community | Health workers |
| ACF, active case finding; ART, antiretroviral therapy; CHWs, community health workers; CT, contact tracing; CTAS, CT tool; DOTS, directly observed treatment (short-course); DOTS-A, DOTS-Ampliado (enhanced DOTS); HHC, household contact; HCW, healthcare worker; IC, index case; IMNCI, Integrated Maternal, Neonatal and Child Illnesses; IPT, isoniazid preventative therapy; LTBI, latent tuberculosis infection; NA, not applicable; NHS, National Health Service; NR, not reported; NTP, National TB Programme; PCR, ​polymerase chain reaction; PACTS, patient-delivered household active case finding for TB; PCF, passive case finding; PTB, pulmonary TB; SARS-CoV-2, severe acute respiratory syndrome coronavirus 2; SOC, standard of care/practice; TB, tuberculosis; TPT, tuberculosis preventative treatment; U5C, children under 5; WHO, World Health Organization.  *Iddirs, a group of local people who have formed a membership-based association and have voluntarily entered into an agreement to help each other during times of difficulty.  **Comparator for Fetzer was extracted from Findlater as they compare to the same usual care, but it is not described in Fetzer**.** | | | | | |

## Table S3. Outcomes reported for studies investigating CT in tuberculosis and Covid-19

| **First author, date; design** | **Study arm name** | **Outcome**  **Category** | **Specific outcome** | **Time point** | **Index (I) / contact (C)** | **N** | **Value** | **Estimate of effect** |
| --- | --- | --- | --- | --- | --- | --- | --- | --- |
| Bai, 2008;^1^ OCC | DOTS expansion | DIP | Total new smear positive cases per area | 1 year | NA | 18,361, 000,000 | 10596 | X^2=362.74, p=0.0001 |
|  | Non-project area |  |  |  | NA | 4,831, 000,000 | 18731 |  |
|  | DOTS expansion | DIP | Notification of positive test/100000 | 1 year | NA | 18,361, 000,000 | 57.7/ 100000 | X^2=1085.1, p=0.0001 |
|  | Non-project area |  |  |  | NA | 4,831, 000,000 | 38.8/ 100000 |  |
| Cavalcante, 2010;^2^ cRCT | DOTS-A | DIP | Incidence rate/100000 | For year 1999 | NA | NA | 339 | [*No difference*] |
|  | DOTS |  |  |  | NA | NA | 340 |  |
|  | DOTS-A | DIP |  | For year 2004 | NA | NA | 305 | MD statistically significant at 5 years (p=0.04) |
|  | DOTS |  |  |  | NA | NA | 358 |  |
| Hanrahan, 2019;^3^ Parallel cRCT | CT (split 50:50 incentive and household) | TR | Treatment initiation ratio comparing the yield of all TB patients started on treatment in both arms | 18 months | NA | NA | NA | OR: 1.06, 95% CI: 0.84 to 1.3 |
|  | Facility-based screening |  |  |  |  |  |  |  |
| Hanrahan, 2023;^4^ RCT | Incentive-based contact investigation | TR | Total numbers of patients that initiated treatment | 36 months | Both | 2720 | 1510 | Adjusted IRR:1.05, 95% CI: 0.97 to 1.13 |
|  | Household-based CT |  |  |  |  | 2664 | 1413 |  |
|  | Incentive-based contact investigation | SAR | Number of contacts diagnosed with TB | 36 months | C | 1940 | 17 | NR |
|  | Household-based CT |  |  |  |  | 1882 | 17 |  |
| Huang, 2019;^5^ CBA | E-DOTS | TR | Treatment success rates (all TB + cases that completed treatment) | NR | I | 473 | range 82.5% - 100% in 2013 when E-DOTs implemented for 2 years | NR |
|  | DOTS |  |  |  |  | 1239 | range 51.4% - 84.6% across whole of DOTs period |  |
| Jerene, 2022;^6^ CBA | Women-led community intervention | TR | Number of eligible children under 15 who initiated treatment | Before-after difference | C | NA | 885 (+963%) | Exp (β): 2.09, 95% CI: 1.88 to 2.32 |
|  | SOC |  |  |  |  | NA | 159(+90.3%) |  |
| Jose, 2020;^7^ CBA | Community-based TB case-finding | DIP | Additional cases of pulmonary Bac + detected compared to baseline | 12 months after baseline | Both | 222 | 8.2% | 8.2% greater (vs 33% worse) case detection in intervention districts compared to baseline than in standard care districts |
|  | Standard care (passive case finding) |  |  |  |  | -397 | -33% |  |
|  | Community-based TB case-finding |  | Additional cases of all TB detected compared to baseline | 12 months after baseline |  | 122 | 2.1% | 2.2% greater (vs 4.1% worse) case detection in intervention districts compared to baseline than in standard care districts |
|  | Standard care (passive case finding) |  |  |  |  | -973 | -34.1% |  |
| Joshi, 2015;^8^ CBA (retrospective) | Intensified childhood tuberculosis case finding | CD | Numbers of childhood TB cases detected vs historical data^+^ | Difference between before and after (2 years) | Both | 1489785 | +6 (33%) per 100,000 | p <0.001 |
|  | Standard DOTS strategy |  |  |  |  | 722597 | +2.2 (16%) per 100,000 |  |
| Kagujje, 2023;^9^ cITS | Multicomponent TB detection strategy | DIP | Childhood TB notifications pre- and post-implementation by site - % change | Difference between period 1 and 2 (2 years) | NR | NR | +352 (225.6%) | NR |
|  | SOC |  |  |  | NR | NR | -85 (-31%) |  |
| Kaswaswa, 2022;^10^ cRCT | PACTS | CD | Household contacts diagnosed with TB | 3 months | I | 106 | 3 | Unadjusted OR: 2.02, 95% CI: 0.48 to 8.51 |
|  | SOC |  |  |  |  | 107 | 6 |  |
|  | PACTS | TR | Children under 5 initiating preventive treatment | 3 months | C | 81 | 47 | Unadjusted OR: 2.02, 95% CI: 0.96 to 4.24 |
|  | SOC |  |  |  |  | 87 | 36 |  |
|  | PACTS | TR | Children under 5 initiating preventive treatment | 9 months | C | 81 | 39 | Unadjusted OR: 1.76, 95% CI: 0.90 to 3.44 |
|  | SOC |  |  |  |  | 87 | 30 |  |
| Khatana, 2019;^12^ Quasi RCT | Interventional group (ACF) | CD | Case detection rate of active pulmonary TB cases | 6 months | C | 598 | 27 | OR: 3.97,95% CI: 1.73 to 9.11, p=0.001 |
|  | Controlled group (SOC) |  |  |  |  | 593 | 7 |  |
| Ketema, 2020;^11^ Stepped wedge RCT | Contact investigation in TB DOTS clinic | CD | Secondary attack rates | 16 months | I | 919 | 10 | P=0.36 [*No difference*] |
|  | Control |  |  |  |  | 684 | 2 |  |
|  | Contact investigation in TB DOTS clinic | TR | Eligible children under 5 initiating preventive treatment | 16 months | I | 919 | 159 | P<0.001 |
|  | Control |  |  |  |  | 684 | 69 |  |
| Martinson, 2022;^13^ cRCT | Home tracing and intensive HIV/TB screening | CD | Case detection rate -contacts diagnosed with TB (from total contacts interviewed) | 15 months | C | 3188* | 51 (1.6%) | HR: 1.33, 95% CI: 0.83 to 2.16 |
|  | SOC |  |  |  |  | 2551* | 31 (1.2%) |  |
|  | Home tracing and intensive HIV/TB screening | Mortality | Number of deaths among contacts (from total contacts measured at baseline) | 15 months |  | 4242 | 42 (1.0%) | HR: 0.72, 95% CI: 0.47 to 1.10 |
|  | SOC |  |  |  |  | 3961 | 49 (1.2%) |  |
| Morishita, 2016;^14^ Quasi cRCT | ACF (plus PCF) | CD | Additional cases compared to baseline | Year 1 | NA | NA | 838 (9.8% change) | NR |
|  | PCF (SOC) |  |  |  | NA | NA | -1120 (-12.3% change) |  |
| Shah, 2020;^15^ cRCT | ACF | DIP | TB incidence rate among household contacts (excluding extrapulmonary)** | 4-16 months | C | NA | 1992.9 per 100,000 contacts/year | IRR: 1.51, 95% CI: 1.21 to 1.88,  p <0.0001 |
|  | PCF |  |  |  |  | NA | 1321.3 per 100,000 contacts/year 1321.3 |  |
| *Covid-19 studies* | | | | | | | | |
| Fetzer, 2021;^16^ cITS | Delayed CT due to the Excel error | DIP | Weekly positive tests per capita | 6 weeks | NA | NA | NA | An additional 0.676 weekly positive tests (SE 0.130, p<0.01) were generated as a result of as a result of each late referral from delayed CT. |
|  | Timely CT |  |  |  |  |  |  |  |
|  | Delayed CT due to the Excel error | DIP | Share of tests returning a positive result per capita | 6 weeks | NA | NA | NA | An additional 0.1 percentage points of tests were positive as a result of each late referral from delayed CT (p<0.01). |
|  | Timely CT |  |  |  |  |  |  |  |
|  | Delayed CT due to the Excel error | DIP | Daily new Covid-19 cases per capita | 6 weeks | NA | NA | NA | An additional 0.606 Covid-19 cases (SE 0.119, p<0.01) were generated as a result of each late referral from delayed CT. |
|  | Timely CT |  |  |  |  |  |  |  |
|  | Delayed CT due to the Excel error | Mortality | Daily new Covid-19 deaths per capita | 6 weeks | NA | NA | NA | An additional 0.007 deaths (SE 0.003, p<0.05) were generated as a result of as a result of each late referral from delayed CT. |
|  | Timely CT |  |  |  |  |  |  |  |
| Findlater, 2023;^17^ cITS | Delayed CT |  |  |  |  | 37373 | 2702 |  |
|  | National contract tracing program | CD | Secondary attack rates | 1 week | C | 121205 | 9419 | Adjusted SAR: INT 7.5%,95% CI:7.3 to 7.8 vs CON 7.7%,95% CI:7.5 to 7.8,  Adjusted OR of infection: 0.99, 95% CI:0.94 to 1.03 |
|  | Delayed CT | CD | Secondary attack rates | 1 week | Secondary C | 6540 | 377 | Adjusted SAR: INT 5.8%,95% CI:5.2 to 6.3 vs CON 5.7%,95% CI:5.4 to 6.0,  Adjusted OR of infection: 0.99, 95% CI:0.88 to 1.12 |
|  | National contract tracing program |  |  |  |  | 23119 | 1315 |  |
|  | Delayed CT | Mortality | Death of a person with a laboratory-confirmed positive SARS-CoV-2 test | 28 days | C | 34047 | 10 (2.9, 95% CI: 1.4 to 5.4 per 10 000  population) | Crude OR: 0.8, 95% CI:0.4 to 1.6 |
|  | National contract tracing program |  |  |  |  | 109907 | 41 (3.7, 95% CI: 2.7 to 5.1 per 10 000  population) |  |
|  | Delayed CT | Mortality | Death of a person with a laboratory-confirmed positive SARS-CoV-2 test | 60 days | C | 34047 | 10 (2.9, 95% CI: 1.4 to 5.4 per 10 000  population) | Crude OR: 0.6, 95% CI:0.3 to 1.1 |
|  | National contract tracing program |  |  |  |  | 109907 | 61 (5.6, 95% CI: 4.2 to 7.1) per 10 000  population |  |
|  | Delayed CT | Mortality | Death of a person with a laboratory-confirmed positive SARS-CoV-2 test | 28 days | Secondary C | 6045 | 0 (0.0, 95% CI: 0.0 to 6.1 per 10 000  population) | Crude OR: 0.5, 95% CI:0.0 to 9.7 |
|  | National contract tracing program |  |  |  |  | 21201 | 3 (1.4, 95% CI: 0.3 to 4.1 per 10 000  population) |  |
|  | Delayed CT | Mortality | Death of a person with a laboratory-confirmed positive SARS-CoV-2 test | 60 days | Secondary C | 6045 | 1 | Crude OR: 0.7, 95% CI:0.1 to 4.0 |
|  | National contract tracing program |  |  |  |  | 21201 | 7 |  |
|  | Delayed CT | UC | Admission to hospital as inpatient | 14 days | C | 34047 | 1121 (3.3%, 95% CI: 3.1% to 3.5%) | Crude OR: 1.1, 95% CI:1.0 to 1.2 |
|  | National contract tracing program |  |  |  |  | 109907 | 3364 (3.1%, 95% CI: 3.0% to 3.2%) |  |
|  | Delayed CT | UC | Admission to hospital as inpatient | 14 days | Secondary C | 6045 | 206 | Crude OR 1.1, 95% CI: 0.9 to 1.2 |
|  | National contract tracing program |  |  |  |  | 21201 | 687 |  |
| Malheiro, 2020;^18^ OCC | CT and quarantine | CD | Case detection rate | 2 months | C | 132 | 16 (12.1%, 95% CI: 7.1 to 18.9) | p=0.125 |
|  | No CT or quarantine |  |  |  |  | 1495 | 138 (9.2%, 95% CI: 7.8 to 10.8) |  |
|  | CT and quarantine | CD | Secondary attack rates | 2 months | I | 98 | 13 (13.3%) | p=0.406 |
|  | No CT or quarantine |  |  |  |  | 453 | 78 (17.2%) |  |
| Raymenants, 2022;^19^ OCC | Extended CT | CD | Case detection rate | 7 days | C | 788 | 14.6 (12.2-17.3) | RR: 2.22, 95% CI:1.72 to 2.88, p<0.0001 |
|  | No care control |  |  |  |  | 1461 | 6.5 (5.3-7.9) |  |
|  | Standard CT | CD | Case detection rate | 7 days | C | 1608 | 17.2 (15.4-19.1) | RR: 2.61, 95% CI: 2.09 to 3.26, P<0.001 |
|  | No care control |  |  |  |  | 1461 | 6.5 (5.3-7.9) |  |
|  | Extended CT | CD | Case detection | 7 days | C | 788 | 42% more cases identified as direct contacts of an index case by extending the CT window | NR |
|  | Standard CT |  |  |  |  | 1608 |  |  |
| Tchakounte, 2024;^20^ cRCT | Digitalised CT | CD | Contacts tested positive declared by the index patient. | 23 weeks | Both | 164/854 | 4 (0.5%) | Unadjusted RR: 0.80, 95% CI:0.21to 2.59, p=0.732; cluster adjusted model RR: 0.80 (0.09-6.85), p=0.835 |
|  | Manual CT (usual care) |  |  |  |  | 149/849 | 5 (0.6%) |  |
| ACF, active case finding; CBA, controlled before and after study; CD, case detection; CI, confidence interval; cITS, controlled interrupted time series; CON, control; cRCT, cluster randomised controlled trial; CT, contact tracing; DIP, disease incidence or prevalence; DOTS, directly observed treatment short-course; DOTS-A, DOTS-Ampliado (enhanced DOTS); HIV, human immunodeficiency virus; HR, hazard ratio; IRR, incidence ratio rate; INT, intervention; MD, mean difference; NA, not applicable; NR, not reported; OCC, observational with concurrent control; OR, odds ratio; PACTS, patient delivered household active case finding for TB; PCF, passive case finding; RCT, randomised controlled trial; RR, risk ratio; SAR, secondary attack rate; SARS-CoV-2, severe acute respiratory syndrome coronavirus 2; SE, standard error; SOC, standard of care; TB, tuberculosis; TR, treatment rate among contacts; UC, unintended consequences.  + additional break down of smear positive, smear negative and extra pulmonary TB available from paper.  * N is lower as only a portion of each group (63% SOC and 75% Int) were interviewed directly and TB status ascertained.  ** data for active TB contacts of index patients including extrapulmonary and contacts detected with active smear positive pulmonary TB available from paper. | | | | | | | | |

# References

1. Bai LQ, Yang HL, Jian XW, He XG, Chen YF, Tang Y*, et al.* Increasing tuberculosis case detection through intensive referral and tracing in Hunan, China. *Int J Tuberc Lung Dis* 2008;**12**:1431-5.

2. Cavalcante SC, Durovni B, Barnes GL, Souza FBA, Silva RF, Barroso PF*, et al.* Community-randomized trial of enhanced DOTS for tuberculosis control in Rio de Janeiro, Brazil. *Int J Tuberc Lung Dis* 2010;**14**:203-9.

3. Hanrahan CF, Nonyane BAS, Mmolawa L, West NS, Siwelana T, Lebina L*, et al.* Contact tracing versus facility-based screening for active TB case finding in rural South Africa: A pragmatic cluster-randomized trial (Kharitode TB). *PLoS medicine* 2019;**16**:e1002796. <https://doi.org/10.1371/journal.pmed.1002796>

4. Hanrahan CF, Nonyane BAS, Lebina L, Mmolawa L, Siwelana T, West NS*, et al.* Household- Versus Incentive-Based Contact Investigation for Tuberculosis in Rural South Africa: A Cluster-Randomized Trial. *Clin Infect Dis* 2023;**76**:1164-72. <https://doi.org/10.1093/cid/ciac920>

5. Huang Y-W, Tsao C-Y, Chen W-W, Yen P-S, Lee J-J, Huang S-S*, et al.* Enhanced directly-observed treatment short-course for tuberculosis control program in mountain areas of Taiwan. *J Infect Dev Ctries* 2019;**13**:123-9. <https://doi.org/10.3855/jidc.10219>

6. Jerene D, Assefa D, Tesfaye K, Bayu S, Seid S, Aberra F*, et al.* Effectiveness of women-led community interventions in improving tuberculosis preventive treatment in children: Results from a comparative, before-after study in Ethiopia. *BMJ Open* 2022;**12**:e062298. <https://doi.org/10.1136/bmjopen-2022-062298>

7. Jose B, Manhica I, Jones J, Mutaquiha C, Zindoga P, Eduardo I*, et al.* Using community health workers for facility and community based TB case finding: An evaluation in central Mozambique. *PloS one* 2020;**15**:e0236262. <https://doi.org/10.1371/journal.pone.0236262>

8. Joshi B, Chinnakali P, Shrestha A, Das M, Kumar AMV, Pant R*, et al.* Impact of intensified case-finding strategies on childhood TB case registration in Nepal. *Public health action* 2015;**5**:93-8. <https://doi.org/10.5588/pha.15.0004>

9. Kagujje M, Nyangu S, Maimbolwa MM, Shuma B, Mutti L, Somwe P*, et al.* Strategies to increase childhood tuberculosis case detection at the primary health care level: Lessons from an active case finding study in Zambia. *PloS one* 2023;**18**:e0288643. <https://doi.org/10.1371/journal.pone.0288643>

10. Kaswaswa K, MacPherson P, Kumwenda M, Mpunga J, Thindwa D, Nliwasa M*, et al.* Effect of patient-delivered household contact tracing and prevention for tuberculosis: A household cluster-randomised trial in Malawi. *PloS one* 2022;**17**:e0269219. <https://doi.org/10.1371/journal.pone.0269219>

11. Ketema L, Dememew ZG, Assefa D, Gudina T, Kassa A, Letta T*, et al.* Evaluating the integration of tuberculosis screening and contact investigation in tuberculosis clinics in Ethiopia: A mixed method study. *PloS one* 2020;**15**:e0241977. <https://doi.org/10.1371/journal.pone.0241977>

12. Khatana GH, Haq I, Khan SMS. Effectiveness, acceptance and feasibility of home-based intervention model for tuberculosis contact tracing in Kashmir. *J Clin Tuberc Other Mycobact Dis* 2019;**14**:19-25. <https://doi.org/10.1016/j.jctube.2019.01.001>

13. Martinson NA, Lebina L, Webb EL, Ratsela A, Varavia E, Kinghorn A*, et al.* Household Contact Tracing With Intensified Tuberculosis and Human Immunodeficiency Virus Screening in South Africa: A Cluster-Randomized Trial. *Clin Infect Dis* 2022;**75**:849-56. <https://doi.org/10.1093/cid/ciab1047>

14. Morishita F, Eang MT, Nishikiori N, Yadav R-P. Increased Case Notification through Active Case Finding of Tuberculosis among Household and Neighbourhood Contacts in Cambodia. *PloS one* 2016;**11**:e0150405. <https://doi.org/10.1371/journal.pone.0150405>

15. Shah L, Rojas Pena M, Mori O, Zamudio C, Kaufman JS, Otero L*, et al.* A pragmatic stepped-wedge cluster randomized trial to evaluate the effectiveness and cost-effectiveness of active case finding for household contacts within a routine tuberculosis program, San Juan de Lurigancho, Lima, Peru. *Int J Infect Dis* 2020;**100**:95-103. <https://doi.org/10.1016/j.ijid.2020.09.034>

16. Fetzer T, Graeber T. Measuring the scientific effectiveness of contact tracing: Evidence from a natural experiment. *Proc Natl Acad Sci U S A* 2021;**118**. <https://doi.org/10.1073/pnas.2100814118>

17. Findlater L, Pierotti L, Turner C, Wensley A, Chen C, Seaman S*, et al.* Evaluating the impact on health outcomes of an event that resulted in a delay in contact tracing of COVID-19 cases in England, September 2020: an observational study. *BMJ open* 2023;**13**:e064982. <https://doi.org/10.1136/bmjopen-2022-064982>

18. Malheiro R, Figueiredo AL, Magalhaes JP, Teixeira P, Moita I, Moutinho MC*, et al.* Effectiveness of contact tracing and quarantine on reducing COVID-19 transmission: a retrospective cohort study. *Public health* 2020;**189**:54-9. <https://doi.org/10.1016/j.puhe.2020.09.012>

19. Raymenants J, Geenen C, Thibaut J, Nelissen K, Gorissen S, Andre E. Empirical evidence on the efficiency of backward contact tracing in COVID-19. *Nat Commun* 2022;**13**:4750. <https://doi.org/10.1038/s41467-022-32531-6>

20. Tchakounte Youngui B, Mambo A, Machekano R, Kana R, Epee E, Tenkeu SZ*, et al.* Improving COVID-19 contact tracing and testing of exposed individuals in Cameroon using digital health technology: a cluster randomised trial. *EClinicalMedicine* 2024;**74**:102730. <https://doi.org/10.1016/j.eclinm.2024.102730>
