# Supplementary material for: The Effectiveness of Contact Tracing to Reduce Transmission of Infectious Diseases During Epidemic or Pandemic Response: Rapid Systematic Review
Source: JMIR Public Health Surveill. 2026 Mar 31;12:e84805. doi: 10.2196/84805 (PMC13080299; doi:10.2196/84805)
Supplement: Multimedia Appendix 8 [file publichealth_v12i1e84805_app8.docx]

## Table S1. Overview of study characteristics, intervention components and findings of studies to improve contact tracing in sexually transmitted diseases

| **Author, date; design, quality** | **Disease; country; setting** | **Intervention** | | **Comparator** | **Outcomes** | | | |
| --- | --- | --- | --- | --- | --- | --- | --- | --- |
|  |  | **Category** | **Key components** |  | **Disease incidence or prevalence** | **Case detection** | **Treatment rates** | **Others** |
| Andersen, 1998;^1^  RCT,  MODERATE | Chlamydia,  Denmark;  Community | PR | Questionnaire  Sampling kit for partners | PR  Request to visit clinic  Contact slip  (Usual care) |  | No difference  (1 outcome) |  |  |
| Apoola, 2009;^2^  RCT,  WEAK | Chlamydia;  UK;  STI Clinics | PR | Contact slips  Urine sampling kit  Fast track appointments  PN interview | PR  Contact slip  Invited to PN interview  Fast track appointments  Swab testing  (Usual care) |  |  | No difference  (1 outcome) |  |
| Brown, 2011;^3^  RCT,  WEAK | HIV;  Malawi;  Clinics | PR/PICT | Contract referral  7 day PN window  Active follow-up  Education | PR  Referral cards  Education  (Usual care) |  | No difference  (1 outcome)^+^ |  |  |
|  |  | PICT | Provider referral within 48 hours  Referral cards  Education | PR  Referral cards  Education  (Usual care) |  | No difference  (1 outcome)^+^ |  |  |
|  |  | PR/PICT | Contract referral  7 day PN window  Active follow-up  Education | PICT within 48 hours  Referral cards  Education |  | No difference  (1 outcome)^+^ |  |  |
| Chen, 2021;^4^  RCT,  MODERATE | HIV;  USA;  Clinics | PR/PICT | Contract referral  7 day PN window  Active follow-up  Referral cards | PR  Referral cards  (Usual care) |  | **Increased**  (1 outcome) |  |  |
| Cherutich, 2017;^5^  cRCT,  WEAK | HIV;  Kenya;  Clinics | PICT | Immediate partner contact by health advisor  Active follow-up  Education | Delayed support (6 weeks) |  | **Increased**  (1 outcome) | **Increased**  (1 outcome) | IPV: no difference |
| Chiou, 2015;^6^  RCT,  STRONG | HIV;  Taiwan;  Hospital | PR/PICT | Intensive PN counselling  Education  PN plan  Emotional support  Counsellor contact | PR/PICT  Education  PN counselling  Standard follow up |  | No difference  (4 outcomes) |  |  |
| Choko, 2021;^7^  cRCT,  WEAK | HIV;  Malawi;  Community | PR | Test kit  Education  Incentive | PR  Referral slips  (Usual care) |  | **Increased**  (1 outcome) | No difference  (1 outcome)^+^ |  |
|  |  | PR | Test kit  Education | PR  Referral slips  (Usual care) |  | **Increased**  (1 outcome) | No difference  (1 outcome)^+^ |  |
| Clark, 2018;^8^  Pilot RCT,  MODERATE | Syphilis;  Peru;  Clinics/ community | PR | Education  Web based PN | PR  Referral cards  (Usual care) |  |  | No difference  (1 outcome) |  |
|  |  | PR | Referral cards  Education | PR  Referral cards  (Usual care) |  |  | No difference  (1 outcome) |  |
|  |  | PR | Web based PN  Education  Referral cards | PR  Referral cards  Education |  |  | No difference  (1 outcome)^+^ |  |
| Culbert, 2023;^9^  RCT,  WEAK | HIV;  Indonesia;  Prison | PR/PICT | Choice of self-tell or assisted PN  Active follow-up by outreach providers  Rapid testing offered | PR  Self-tell  (Usual care) |  | **Increased**  (1 outcome)^+^ |  |  |
| Dibia, 2024;^10^  OCC,  WEAK | HIV;  Nigeria;  Health facility | PICT | Elicitation form deposited anonymously | PR/PICT  (Usual care) |  | No difference  (1 outcome) |  |  |
| England, 2005;^11^  OCC,  WEAK | Chlamydia;  Australia;  Clinic | PR/PICT | Additional staff to support CT | PR/PICT  (Usual care) |  | No difference  (1 outcome) |  |  |
| Estcourt, 2012;^12^  nrCT,  WEAK | Multiple STIs;  UK;  STI Clinics | PR | Contact slip  Interview  Education  Pharmacist consultation | PR  PN interview  Contact slip  Education  (Usual care) |  |  | **Increased**  (1 outcome) |  |
|  |  | PR | Contact slip  Interview  Education  Telephone consultation | PR  PN interview  Contact slip  Education  (Usual care) |  |  | **Increased**  (1 outcome) |  |
|  |  | PR | Contact slip  Interview  Education  Pharmacist consultation | PR  Contact slip  Interview  Education  Telephone consultation |  |  | No difference  (1 outcome) |  |
| Estcourt, 2015^13^ Escourt 2016;^35^  Pilot RCT,  WEAK | Chlamydia;  UK;  Primary care | PR | APT pack  Telephone consultation  Testing kit | PR  Education  (Usual care) | No difference  (1 outcome) |  | No difference  (1 outcome) |  |
|  |  | PR | APT pack  Pharmacist consultation  Testing kit | PR  Education  (Usual care) | No difference  (1 outcome) |  | No difference  (1 outcome) |  |
|  |  | PR | APT pack  Telephone consultation  Testing kit | PR  APT pack  Pharmacist consultation  Test kit | No difference  (1 outcome) |  | No difference  (1 outcome) |  |
| Estcourt, 2022,^14^ Escourt, 2024;^37^ cRCT,  WEAK | Chlamydia;  UK;  STI Clinics | PR | Telephone assessment  Antibiotic prescription  Testing kit | PR  PN interview  HIV screen  Chlamydia treatment  Active follow-up  (Usual care) | No difference  (1 outcome) |  | No difference  (1 outcome) |  |
| Faxelid, 1996;^15^  RCT,  MODERATE | Multiple STIs;  Zambi;  Health centre | PR/PICT | Individual counselling  Education  Contact slips  Provider referral offered | No CT  Usual care |  |  | **Increased**  (1 outcome)  No difference  (1 outcome) |  |
| Heumann, 2017;^16^  OCC,  STRONG | Syphilis, HIV;  USA;  Clinic/ community | PICT | In-person partner services interviews | PICT  Telephone partner services interview (Usual care) |  | **Increased**  (1 outcome)  No difference  (1 outcome) | **Increased**  (1 outcome) |  |
| Hu, 2021;^17^  RCT,  MODERATE | HIV;  China;  Clinic | PR/PICT | Partner referral  Active follow-up  CHW contacts partner,  Self-testing kit  Remote result submission  Counselling/education | PR  (Usual care) |  | No difference  (1 outcome) |  |  |
| Katz, 1988;^18^  RCT,  MODERATE | Multiple STIs;  USA;  STI Clinics | PICT | Active follow-up | PR  Referral slips  (Usual care) |  |  | **Increased**  (1 outcome) |  |
|  |  | PR | Interview | PR  Referral slips  (Usual care) |  |  | No difference  (1 outcome) |  |
|  |  | PICT | Active follow-up | PR  Interview |  |  | **Increased**  (1 outcome) |  |
| Kerani, 2011;^19^  RCT,  WEAK | Chlamydia, gonorrhoea;  USA;  Clinic/online | PR | Web-based PN service | PR  Standard PN  (Usual care) |  |  | No difference  (1 outcome) |  |
| Kissinger, 2005;^20^  RCT,  WEAK | Multiple STIs;  USA;  Clinic/ community | PR | Booklet/cards to enhance treatment | PR  (Usual care) | **Reduced**  (1 outcome) |  | **Increased**  (1 outcome) |  |
| Kissinger, 2006;^21^  RCT,  STRONG | Multiple STIs;  USA;  Clinic/ community | PR | Booklet/cards to enhance treatment | PR  (Usual care) | No difference  (1 outcome) |  |  |  |
| Landis, 1992;^22^  RCT,  WEAK | HIV;  USA;  County health departments | PR/PICT | Patient option to notify  Provider notification  Active follow up | PR  Education  Referral slips  Active follow-up after 1 month  (Usual care) |  | No difference  (2 outcomes)^+^ |  |  |
| Low, 2006;^23^  RCT,  MODERATE | Chlamydia;  UK;  Community | PICT | Nurse-led PN  Interview  Education  Contact slips  Researcher-led follow up | PR/PICT with active follow-up  Contact slips  Standard PN  (Usual care) |  | No difference (2 outcomes) |  |  |
| Lugada, 2010;^24^  cRCT,  MODERATE | HIV;  Uganda;  Clinic/ community | HI | Home visits  Education  Contact cards  Counselling & testing | PR  VCT vouchers  (Usual care) |  | **Increased**  (1 outcome) |  |  |
| Lukac, 2021;^25^  OCC,  MODERATE | Syphilis;  Canada;  Community | PR | Patient-initiated PN with nurse support  Education  Resources | PICT  Education  Resources |  | No difference  (1 outcome) | **Increased**  ^+^  (1 outcome) |  |
| Luo, 2020;^26^  OCC,  WEAK | HIV;  China;  Clinic | PR/PICT | Provider support  Dual referral model  Active follow up | PR  Active follow up |  | Only raw data  (1 outcome) |  |  |
|  |  | PICT | Provider referral  Active follow up | PR  Active follow up |  | Only raw data  (1 outcome) |  |  |
|  |  | PR | Testing kit  Patient referral  Active follow up  Education | PR  Active follow up |  | Only raw data  (1 outcome) |  |  |
| Malave, 2008;^27^  OCC,  WEAK | HIV;  USA;  Clinic/ hospital | PICT | PN led by STD clinic | PICT led by non-STD clinic |  | No difference  (1 outcome) |  |  |
| Mathews, 2020;^28^  RCT,  WEAK | STIs;  South Africa;  STI clinic | PR/PICT | Education  Training  Provider assisted PN | Health education  (Usual care) | No difference  (1 outcome) |  |  | IPV:  No difference (1 outcome)  Abandonment: **Greater risk**  (1 outcome) |
| Oh, 1996;^29^  nrCT,  WEAK | Chlamydia; gonorrhoea;  USA;  Clinic | PICT | Provider assisted PN | PR  Referral slips  (Usual care) |  |  |  |  |
| Ostergaard, 2003;^30^  RCT,  MODERATE | Chlamydia;  Denmark;  Community | PR | Home sampling | PR  Office sampling  (Usual care) |  | **Increased**  (2 outcomes) | No difference  (1 outcome) |  |
| Parkes-Ratanshi, 2020;^31^  RCT,  STRONG | Syphilis;  Uganda;  Antenatal clinic | PR | SMS reminders  Notification slips | PR  Referral slips  Active follow-up  (Usual care) |  | No difference  (1 outcome |  |  |
|  |  | PR | Phone call reminders  Notification slips | PR  Referral slips  Active follow-up  (Usual care) |  | No difference  (1 outcome) |  |  |
|  |  | PR | SMS reminders  Notification slips | PR  Phone call reminders  Notification slips |  | No difference  (1 outcome |  |  |
| Peterman, 1997;^32^  RCT,  MODERATE | Syphilis;  USA;  Community | PICT | Provider referral | PR/PICT  Contract referral |  | No difference  (1 outcome)^+^ | No difference  (1 outcome) ^+^ |  |
|  |  | PICT | Provider referral  Option to take blood in field | PR/PICT  Contract referral |  | No difference  (1 outcome) ^+^ | No difference  (1 outcome) ^+^ |  |
|  |  | PICT | Provider referral | PICT  Option to take blood in field |  | No difference  (1 outcome) ^+^ | No difference  (1 outcome) ^+^ |  |
| Potterat, 1997;^33^  nrCT,  WEAK | Gonorrhoea;  USA;  Community | PR | Self-referral  Contact slips  Interview  Education | PR/PICT  PN interview  Active follow-up  (Usual care) |  | No difference  (1 outcome) ^+^ |  |  |
| Schwebke, 2010;^34^  RCT,  MODERATE | Trichomoniasis;  Cameroon;  Community | PR/PICT | Interview  Contract referral  Active follow up  Assisted PN | PR  Treatment (metronidazole)  Education  (Usual care) | No difference  (2 outcomes) |  |  |  |
| Wilson, 2009;^35^  RCT,  STRONG | Chlamydia, gonorrhoea;  USA;  STI clinic | PR | Individual counselling  Referral slips  Education  Interview | PR  Education  Referral slips | **Decreased**  (1 outcome) |  |  |  |
| ^+^, no statistical analysis provided for comparison; APT, accelerated partner therapy; CHW, community health worker; cRCT, cluster RCT; HIV, human immunodeficiency virus; IPV, intimate partner violence; nrCT, non-randomised controlled trial; OCC, observational study with concurrent control group; PICT, provider-initiated contact tracing; PR, patient referral; PN, partner notification; PR/PICT, contract referral; RCT, randomised controlled trial; SMS, short messaging service; STI, sexually transmitted infection; UK, United Kingdom; USA, United States of America; VCT, voluntary HIV counselling and testing. | | | | | | | | |

## Table S2. Characteristics of interventions to improve contact tracing in sexually transmitted infections

| **First author, date** | **Study arm name** | **Study arm summary** | **Duration** | **Setting** | **Who delivered** |  |  |  |
| --- | --- | --- | --- | --- | --- | --- | --- | --- |
| *Chlamydia studies* | | | | | | |  |  |
| Andersen, 1998^1^ | Home sampling | Women in the intervention group were asked to complete a questionnaire, including the number of male sexual partners over the preceding six months, and to supply their partners with an envelope containing a 10 ml sterile container, information on collecting the first urine sample of the morning, and a prepaid envelope for returning the sample to the hospital laboratory. | NR | Community | Participants |  |  |  |
|  | SOC (doctor sampling) | Request for partner to visit doctor; contact slip with prepaid envelope for returning urethral swab sample. | NR | Community and clinic | Participants and healthcare staff |  |  |  |
| Apoola, 2009^2^ | Urine testing group | Patients were seen by the health advisor and contact details were recorded. Contact slips were given to the index patient as well as a urine sampling kit with clinic details for sexual contact to bring in the sample and get treated. Sexual contacts were seen rapidly with a fast-track appointment system for chlamydial infection. They also had a full history and examination if accepted. All contacts were invited to see the health advisor for a PN interview and to have screening tests for other STIs. | 28 days | STI clinic | Health advisor |  |  |  |
|  | SOC (swab testing) | Health advisor recorded details of patient’s contacts. Contact slips given to the index patient to give to male sexual contacts to bring into clinic for testing by urethral swab and treatment. Sexual contacts in both arms were seen with the same fast track appointment system for sexual contacts of patients with chlamydial infection. All contacts were also invited to a PN interview. | 28 days | STI clinic | Health advisor |  |  |  |
| England, 2005^11^ | Public health officer (PHO) CT through Communicable diseases control section (CDCS) | During the study period, CDCS utilised 2 part-time PHOs (registered nurses with sexual health experience). They provided assistance with CT to practitioners and clinics across the Australian Capital Territory. The PHOs were based at the clinic and collectively spent ~5 hours per working week on CT. Index cases (ICs) contacted by PHO, counselling and information provided and if assistance accepted by ICs PHO contacts partners, otherwise assumed patient initiates notification. | 13 months | Clinic | Clinician or PHO |  |  |  |
|  | SOC (clinic CT) | The clinic performs its own inhouse CT by either a registered nurse or centre clinician. PN was provider or patient initiated. | 13 months | Clinic | Clinician or registered nurse |  |  |  |
| Estcourt, 2015^13^/2016^36^ | Accelerated Partner Therapy (APT) hotline + standard PN | Telephone consultation and invitation for clinic-based HIV and syphilis screening for sex partners. Sex partners collect APT Pack (containing patient information leaflet about the study, chlamydia urine sample kits and instructions, antibiotics, condoms, chlamydia information leaflet, prepaid envelope and packaging for returning the sample to the study clinic) and post back completed chlamydia NAAT urine sample kit. Results managed by a specialist clinic. ICs contactable by telephone and willing to be re-tested for re-infection/persistence of infection were sent urine sample kit 4-6 weeks later. | 6 weeks | Primary care | Healthcare professionals |  |  |  |
|  | APTPharmacy + standard PN | Consultation with a sexual health trained community pharmacist for sex partners. Pharmacist gives sex partner APT pack at the time of consultation. ICs contactable by telephone and willing to be re-tested for re-infection/persistence of infection were sent urine sample kit 4-6 weeks later. | 6 weeks | Primary care | Healthcare professionals (pharmacist) |  |  |  |
|  | SOC (standard PN) | Standard PN involved patient referral (index patient advised to notify their sex partner of the need for treatment). In the contraception and sexual health (CASH) services written information about chlamydia and details of local sexual health services were passed to the index patient to give to her sex partner. ICs contactable by telephone and willing to be re-tested for re-infection/persistence of infection were sent urine sample kit 4-6 weeks later. | 6 weeks | Primary care | Healthcare professionals |  |  |  |
| Estcourt, 2022^14^/2024^37^ | APT + SOC | APT involved a healthcare professional assessing the partner(s) by telephone, then giving index patient antibiotics and STI self-sampling kits for partner(s). | 12-14 weeks | Sexual health clinic | Healthcare professionals |  |  |  |
|  | SOC | Healthcare professionals advised index patients to inform their partner(s) of the need for testing (routinely, a comprehensive STI and HIV screen) and chlamydia treatment (doxycycline). Written/online information provided. Follow-up of all index patients by telephone at 2 weeks and 12–13 weeks. | 12-14 weeks | Sexual health clinics | Healthcare professionals |  |  |  |
| Low, 2006^23^ | Practice nurse | PN initiated by a practice nurse immediately after diagnosis. This strategy involved a PN interview (identifying sexual contacts in previous 6 months); patient referral using contact slips for each partner; and advice and information. Contact slips included study genitourinary medicine clinic details, and a request to return the slip to the study centre. Each practice nominated 1-2 nurses, who were trained in PN and study procedures. A research health adviser was available to support practices and carried out telephone follow up. Practice nurses did not follow-up ICs. | NR | General practices | General practice nurses |  |  |  |
|  | SOC (GUM clinic) | The subjects were given research health advisor details. If the IC did not phone the clinic within a week the health advisor made up to two attempts to contact them. Health advisors carried out PN using standardised protocols, including provider referral (immediately informing partners on behalf of the patient), or conditional referral (contacting partners if the patient had not done so after an agreed period) and issued contact slips. | NR | General practices | Research health advisor |  |  |  |
| Ostergaard, 2003^30^ | Home sampling | Partners in the home sampling group received specimen collection kits and mailed their samples directly to the diagnostic laboratory in pre-paid and pre-addressed envelopes. | 4 months | Community | Participants |  |  |  |
|  | SOC (office sampling) | Partners in the office sampling group brought the specimen collection kit into the office of a healthcare provider to obtain a sample along with a letter explaining the study and the importance that the health care provider used the provided specimen collection kit. | 4 months | Community and clinic | Participants and healthcare staff |  |  |  |
|  |  | *Chlamydia & Gonorrhoea studies* |  |  |  |  |  |  |
| Kerani, 2011^19^ | InSPOT (web-based partner notification service) + SOC | InSPOT participants were given an opportunity to use inSPOT on a clinic computer. They also received a small, printed card with the site's internet address. Study staff described the site to persons enrolled over the telephone and informed them of the site's URL. | 2 weeks | Clinic and internet | Study staff |  |  |  |
|  | SOC (standard partner management) * | The standard partner services interview was carried out (including partner enumeration) before randomisation. In all arms, staff developed a PN plan for each of a participant's sex partners from the prior 60 days and offered to directly notify each partner with contact information. In standard partner management no additional procedures occurred. | 2 weeks | Clinic | Study staff |  |  |  |
| Oh, 1996^29^ | Provider referral | Partner contact details obtained from the IC. Staff attempted to contact partner(s). If contact unsuccessful after 3 phone attempts and 3 letters, the provider-referral was coded as "failure". (By phone) Each partner informed that he had been named a sexual contact and free STD treatment was offered at the clinic. (By letter) Partners informed of their exposure and urged to call for an appointment. A follow-up call occurred if appointments were not kept, and if care had been sought elsewhere, patients were requested to notify the clinic. ICs were anonymous. ICs were recalled, if the provider-referral outcome could not be ascertained through other means. | 6 weeks | Clinic | Healthcare staff |  |  |  |
|  | SOC (patient referral) | In self-referral, partners were provided referral-slips and instructed to call the clinic for an appointment by the IC. The referral-slip had clinic details, infection information and need for treatment. A follow-up phone contact was made to assess outcome of partner notification. ICs interviewed by case manager 2-6 weeks post-treatment. | 6 weeks | Clinic | Participants |  |  |  |
| Wilson, 2009^35^ | Enhanced patient referral | Programme of 2 sessions: Session 1 (in clinic at time of diagnosis) involved one-on-one counselling where client behaviours related to risk of infection, identification of sexual partners, development of a PN plan, role-playing exercises, and a signed contract to notify sexual partners were discussed. Support materials about PN were provided with referral slips with information on free, confidential STI testing and treatment. No identifying information was collected on sexual partners; Session 2, telephone or in person interview 2 to 10 weeks after the first session (target of 4 weeks). This included progress review and discussion of any barriers. All health educators were trained on interviewing and sexual partner elicitation and on STI epidemiology. | 2-10 weeks | STI clinic | Health educators / a disease intervention specialist (DIS) trainer |  |  |  |
|  | Control group (standard PN) | SOC PN occurred at each site. Patients also met programme health educator, and discussed the clinic visit, diagnosis, treatment and prevention. Patients were provided with referral slips to give to sexual partners. The patient sat with the health educator for this interaction (different from SOC) to allow for similar rapport building opportunities between groups, and to help standardize messages provided to participants. | NR | STI clinic | Health educator |  |  |  |
|  |  | *Chlamydia & NGU studies* |  |  |  |  |  |  |
| Katz, 1988^18^ | Field follow-up | Stepwise procedure that used increasing time and resources until the partner was contacted or the search was terminated because of insufficient information. First, clinic records were searched to see whether the partner had been treated in the clinic the same day or the few days prior to the IC visit (these were excluded). If not, DIS attempted a phone call (obtained from the IC or if not possible from other sources). If the partner could not be reached by phone within 24 hours, a letter was sent. At the same time, the DIS attempted to contact the partner at her home or place of employment, as well as sending a letter. If the partner was not home when the DIS visited, a second letter was left. Finally, a DIS verified with the treating physician that the proper treatment was given if treatment was sought elsewhere. | 6 months | STI clinic, home visits | DIS + clinic staff |  |  |  |
|  | Interview only | Counselling was provided by trained DIS who gathered the names of the contacts only. ICs were advised of the importance of referring their partners to the clinic, but no referral letters were distributed, and no additional attempt was made to locate the women for treatment. In this self-referral strategy, partners who sought treatment at another location could not be identified. | 6 months | STI clinic | DIS |  |  |  |
|  | SOC (nursing referral) | Counselling provided by only the nurse. Patients advised of the importance of referring his partners for treatment and were given the requested number of referral letters. When partners attended the clinic, they were matched to the ICs via referral letters or the clinic's database. | 6 months | STI clinic | Clinic nurse |  |  |  |
|  |  | *Chlamydia, gonorrhoea and NGU studies* |  |  |  |  |  |  |
| Estcourt, 2012^12^ | APTHotline + standard PN | A health advisor/senior nurse assessed partners by phone using standard protocols. In clinic A, the partner could collect the treatment pack from the clinic or have it delivered by the index patient. In clinic B, an additional doctor was also needed to assess the partner by phone. Both APT options included an ‘assertive invitation’ to the partner to attend a local sexual health clinic for a fast-track HIV and syphilis test. | 6 weeks | STI clinic | Health advisor or senior nurse |  |  |  |
|  | APTPharmacy + standard PN | Participating pharmacies held consultations for sexual partners with a trained community pharmacist who supplied the treatment. Each clinic linked to 3 community pharmacies, in which 1-2 pharmacists undertook training in the clinical management of partners of people with chlamydia, gonorrhoea or NGU by Patient Group Directive. This option also included an ‘assertive invitation’. | 6 weeks | STI clinic | Trained community pharmacist |  |  |  |
|  | SOC (routine PN) | Routine PN at Clinic A involved an interview with a health advisor/nurse practitioner, provision of contact slip, infection-specific information and advice for partner to attend clinic/general practice for treatment. Routine PN at Clinic B was similar, with additional provision of letter for partner to take to health provider in which possible treatment regimens are described. | 6 weeks | STI clinic | Health adviser or nurse practitioner |  |  |  |
| Kissinger, 2005^20^ | Booklet-enhanced partner referral (BEPR) | BEPR involved ICs being given 4 cards with information for partners and treatment guidelines for professionals who would see partners. The partners could then present this card at the clinic of their choice to help the clinician better treat them. Men with > 4 partners were given additional booklets. | 2-8 weeks | STD clinic | Clinicians |  |  |  |
|  | Standard of care (partner referral (PR)) ** | Men instructed to tell their partners that they needed to go to a clinic for STD evaluation and treatment. | 2-8 weeks | STD clinic | Clinicians |  |  |  |
|  |  | *Gonorrhoea studies* |  |  |  |  |  |  |
| Potterat, 1977^33^ | Study group (self-referral) | A short interview (3-5 minutes) discussing the nature and implications of the disease and the importance of self-referral of contacts. Patients given contact slips to give to sex partners. No names were elicited. Patient re-interviewed 7-10 days later to elicit identifying information on contacts. Contacts sought to learn their clinical outcomes, and where necessary, refer them for treatment. | 7-10 days | Clinic | Clinic staff |  |  |  |
|  | SOC (standard interview) | Standard interview was carried out, including eliciting names and identifying contacts data. The patient was asked to refer contacts for treatment (unless the Programme's help was requested) and informed them that the Programme would seek named contacts who did not appear in seven to ten days. | 7-10 days | Clinic | Clinic staff |  |  |  |
| *HIV studies* | | | | | |  |  |  |
| Brown, 2011^3^ | Contract referral | Participants given seven days to notify partners, then health care providers contacted partners who had not reported and counselled them to visit the clinic. All were provided referral cards to give to their partners and were counselled on the importance of safe sex behaviour. | 30 days | STI clinic | Healthcare provider |  |  |  |
|  | Provider referral | Notification in the provider referral group occurred within 48 hours. Community outreach workers or nurses performed notification. A standard protocol for community contact was followed. All were provided referral cards to give their partners and counselled on the importance of safe sex behaviour. | 30 days | STI clinic | Community outreach worker (trained HIV testing counsellors) or nurse |  |  |  |
|  | SOC (Patient referral) | ICs notified partners themselves and initiated HIV care. All were provided referral cards to give their partners and counselled on the importance of safe sex behaviour. | 30 days | STI clinic | Index case |  |  |  |
| Chen, 2021^4^ | Contract partner notification | Intervention combined contract PN, social contact referral and acute HIV infection (AHI) screening. Contract PN: IC referred sexual partners for testing but partner contact information also collected in case partner does not return for testing within 7 days. Participant-specific referral cards used but ICs remained anonymous to partners. Social contact referral: participants referred acquaintances who might benefit from HIV/STI services. Names or pseudonyms used but no contact details collected. Participants used referral cards with referral type (sexual partner vs. social contact) distinguishable by card colour. AHI screening: HIV-seronegative and -serodiscordant participants tested for HIV RNA. Study staff contacted patients with AHI within one day of a positive result and referred them to HIV care and offered enrolment into the intervention arm. | 7 days | Outpatient STI | Clinic staff |  |  |  |
|  | SOC (partner referral) | Malawian SOC: participants asked to refer up to 5 sexual partners from the last six months to the STI clinic for HIV testing with coded referral cards. | NR | Outpatient STI | Index case |  |  |  |
|  | Immediate assisted partner services | Health advisors immediately contacted sex partners, offered HIV home-testing (or other convenient venue), and referred HIV positive patients to a HIV clinic. Health advisors made 3 attempts to contact by phone. If unsuccessful, they attempted to contact the partner in person at least twice using details from ICs. Those who refused testing or tested negative were encouraged to test at a later date or retest as a couple and counselled on HIV prevention methods. Health advisors encouraged ICs to notify sex partners of HIV-positive status as per Kenyan SOC. | 6 weeks | Clinic | Health advisors |  |  |  |
| Cherutich, 2017^5^ | Delayed | No additional support was delivered to promote PN or testing until 6 weeks post-enrolment. ICs were encouraged to notify sex partners of HIV-positive status as per Kenyan SOC. | 6 weeks | Clinic | Health advisors |  |  |  |
| Chiou, 2015^6^ | Experimental group | SOC plus early multiple-time PN counselling, consisting of ~60 minutes of counselling (upon recruitment) followed by a second ~90-minute counselling session (post diagnosis). Like in SOC, treatment information  given, PN methods introduced, PN plan set up, emotional support provided, and resource referrals furnished. Contact with counsellor either by phone 8-5 or 24hours/day through communication apps/email. | 150 mins | Clinic | PN trained counsellor |  |  |  |
|  | SOC (PN) | Post diagnosis ICs received 60 minutes of PN counselling. This included an introduction to PN and available resources, setting up a PN plan (including choosing a PN procedure [from patient referral, provider referral, dual referral, and contract referral]), and providing emotional support. Contact with counsellor either by phone 8am-5pm or 24 hours/day through communication apps/email. One routine follow-up interview 2 months later. Within 1 week of receiving contact details, chosen PN method was used to inform partners, followed by preliminary HIV screening and a western blot analysis. Partners who tested negative at first screening were tested again after 3 months, and if negative follow-up ceased. | 60 mins | Clinic | PN trained counsellor |  |  |  |
| Choko, 2021^7^ | HIV self-testing (HIVST) | Women in antenatal clinic (ANC) cohort or patients in index cohort were provided with one HIV self-test kit per sexual partner for secondary distribution, with a brief demonstration (~5 minutes) on how to carry out and interpret the test. Partners were encouraged to attend clinic services to confirm all positive results or results that were serodiscordant with their regular partner. The participant’s own interpretation of HIVST results and results obtained from retesting by the HIV provider were compared. Follow-up interviews in ANC group within 28 days ascertained male partner testing. | 28 days | ANC and HIV testing services in government health centres | Government staff managed secondary HIVST kit distribution and HIV care. Independent interviewer completed follow-up interviews in ANC group |  |  |  |
|  | HIVST + financial incentive | Women in ANC cohort or patients in index cohort were, in addition to the test kit and demonstration, provided with a leaflet offering their male partner or sexual contacts $10 to retest at the clinic. Follow-up interviews in ANC group within 28 days ascertained male partner testing. | 28 days | ANC and HIV testing services in government health centres | Government staff managed secondary HIVST kit distribution and HIV care. Independent interviewer completed follow-up interviews in ANC group |  |  |  |
|  | Enhanced SOC | Referral slips used to invite male partners of women in ANC cohort or sexual partners of patients in the index cohort to attend HIV testing within 28 days. | 28 days | ANC and HIV testing services in government health centres | Clinic staff |  |  |  |
| Culbert, 2023^9^ | Assisted partner notification (APN) | Participants in Impart APN arm could choose per partner between self-tell or APN. APN: trained nurses and community outreach healthcare providers collaborated in locating and notifying partners keeping ICs anonymous. Impart notifiers initially contacted named partners by phone and asked to meet in person. Notification by phone occurred if meeting not possible. APN notifiers conducted home visits to find unreachable partners. Partners were notified by the Impart team on HIV exposure anonymously and then offered immediate HIV rapid testing. Alternatively, partners were referred to a clinic of their choice from a list of nearby free services. Meanwhile, those who chose to tell ≥1 partners themselves were given the same list of services to share with partners. Participants who chose to tell partners themselves self-reported this. | 6 weeks | HIV subspecialty clinics located within each jail and prison facility/home visits | Project-trained APN counsellors, Impart APN-trained nurses and community outreach healthcare provider |  |  |  |
|  | SOC (self-tell notification) | Subjects were assisted in developing a PN plan and coached in telling their at-risk partners within 6 weeks. Permission sought to verify who was notified after 6 weeks. Inmates could notify partners by phone, mail or in-person during visitation. They could have an HIV counsellor present. Participants were reinterviewed about their notification experience. Participants were asked to self-report on which partners they had informed. | 6 weeks | HIV subspecialty clinics located within each jail and prison facility | Project-trained APN counsellors |  |  |  |
| Dibia, 2024^10^ | Elicitation box | ICs wrote contact details of sexual partners on a coded, anonymous elicitation form. The health care provider collected it from the elicitation box, completed the index tracing form, contacted the ICs’ partners, and updated the index register. The elicitation box was placed in a conspicuous corner of the facility. | NR | Health facility | HCW |  |  |  |
|  | SOC (conventional method/patient referral) | ICs provided details directly to healthcare providers. Elicited partners were reached for HIV testing via the IC (patient referral), the provider (provider-assisted referral), or both (dual and contracted referral approaches). | NR | Health facility | HCW |  |  |  |
| Hu, 2021^17^ | APN | SOC + APN. APN: ICs had 2 options: HIVST kits for at-home testing of their partners or providing partners’ contact information and consent for a trained CHW to anonymously notify partner(s) of potential HIV exposure and invite them for HIV testing in clinic. For the HIVST kits option, ICs were trained to use kits by research staff and provided kits for all partners. ICs distributed kits to partners and asked them to provide online consent. After partners completed self-testing, ICs sent the photographs of results through the research website. CHW outreach: ICs shared their sexual partners’ contact details. A CHW contacted partners using a standardised message involving reimbursement information and no information on the IC. CHW discussed overcoming barriers to clinic visits, and scheduled appointments. CHW made 3 phone attempts then 3 social media attempts to contact each partner. Follow-up phone calls to ICs at 2, 3 and 4 months by researcher to ask of partner testing and result. | 4 months | Outpatient HIV clinic | CHW |  |  |  |
|  | SOC (patient PN) | ICs were asked to disclose their HIV status to their sexual partners and suggest they get an HIV test (patient referral). Follow-up phone calls to ICs at 2, 3 and 4 months by researcher to ask of partner testing and result. | 4 months | Outpatient HIV clinic | CHW |  |  |  |
| Landis, 1992^22^ | Provider referral | Subjects could choose to notify some or all partners (sexual and needle sharing) themselves. The public health counsellor (PHC) immediately attempted to notify remaining partners and partners who had not attended the health department within 2 weeks. Methods used were similar to those used in cases of syphilis, with the exception that HIV exposure was initially discussed in person rather than by mail/phone. Coloured cards with identification codes were provided to ICs to give their partners, as a means of matching attending partners to subjects. | 4 weeks | Public health departments | PHC |  |  |  |
|  | Standard of care (patient referral) | Subjects asked to locate partners (sexual and needle sharing), give them coded cards and request that partners come in for counselling, testing, or both within a month. Counsellors gave advice on talking to partners about HIV exposure. After 1 month, counsellors attempted to locate all non-attending partners, to determine whether they were aware of their HIV exposure. | 4 weeks | Public health departments | PHC |  |  |  |
| Lugada, 2010^24^ | Home-based ART delivery + voluntary HIV counselling and testing (VCT) | Household members were visited by trained lay field officers. Household members received basic HIV and ART education, and consenting individuals were offered VCT in a private place at or near their home, as of Ugandan policy. Routine VCT cards were used to record HIV testing data and results. VCT cards were completed for all household members present during home visits. | NR | Community/home visits | Trained lay field officers |  |  |  |
|  | SOC (clinic-based ART delivery + VCT) | Participants given free VCT vouchers for each household member and encouraged to invite all household members to clinic for VCT by a trained counsellor. | NR | Community | Trained counsellors |  |  |  |
| Luo, 2020^26^ | Couples HIV counselling and testing (CHCT) | Based on dual referral, where a trained provider accompanied and provided support to ICs when notifying partners. The IC returned to the VCT clinic with his sexual partner to receive joint HIV testing and counselling. The IC is not requested to disclose his HIV status to his sexual partner prior to the CHCT session. VCT staff followed up with phone reminders once monthly until the IC and his sexual partner complete the CHCT session (within 3 months). | 3 months | VCT clinic | VCT staff | | | |
|  | Information-assisted Partner Notification (IAPN) | Based on provider referral, in which a trained provider confidentially contacts and offers services to the patient’s partner(s) directly. IC is requested to provide contact information. VCT staff contact sexual partner and communicate their risk of HIV infection. They also promote VCT and provide related information. VCT staff ensure the IC remains anonymous and follow-up the partner once monthly until they attend a VCT for testing within 3 months. | 3 months | VCT clinic | VCT staff |  |  |  |
|  | HIVST | ICs received a demonstration before or during HIVST, then an information session on the testing kit by VCT staff, then received a kit, assisted in testing his partner, and returned the kit to VCT clinic within 2 weeks. Upon a positive result, the IC is requested to either (a) request his partner comes to the VCT clinic for a confirmatory test; or (b) provide contact information for his partner so the VCT staff may initiate tracing. VCT staff follow up with a phone call to the IC once monthly until his partner attends HIV testing (within 3 months). | 3 months | VCT clinic | VCT staff |  |  |  |
|  | Patient referral | ICs notify their sexual partner of their positive status and suggest HIV Testing Services (HTS). ICs provide details of sexual partners to confirm their identity during the following HIV testing. HTS staff follow-up IC once monthly by phone until partner attends a service for HIV testing (within 3 months). | 3 months | VCT clinic | VCT staff |  |  |  |
| Malave, 2008^27^ | STD clinic partner notification | For ICs diagnosed in STD clinics, DIS elicited the partners and notified them. In addition, DIS also conducted PNs (if partner's locating information was sufficient) on partners referred to DIS from ICs diagnosed in non-STD facilities that were elicited by community clinicians. During the period of this study, DIS did not interview ICs diagnosed in non-STD facilities. | 1 year | STD clinics | DIS |  |  |  |
|  | Non-STD clinic partner notification | Non-STD clinic facilities had community clinicians carry out partner notification. | 1 year | Non-STD facilities including hospitals, clinics, and private physician offices | Community clinician |  |  |  |
|  |  | *STI (unspecified) studies* |  |  |  |  | |  |
| Mathews, 2020^28^ | Enhanced partner notification (ePN) | ePN involved: STI information provision from counsellors; motivation-enhancing and skills building exercises; a menu of PN options including patient referral (using a referral card, or inviting partner to attend clinic with IC) and anonymous provider-assisted options; assisting the IC in developing a PN plan for each partner; and inviting the IC to role-play the PN. | 45-minute session | STI clinic | Counsellors |  | |  |
|  | SOC (health education (HE)) *** | Standardised version of counsellor activities. HE included counsellor-provided information about STIs, advice and answering questions. | 20-minute session | STI clinic | Counsellors |  | |  |
|  |  | *STI (syphilis, chancroid, lymphogranuloma venereum, gonorrhoea, trichomoniasis) studies* |  |  |  |  | |  |
| Faxelid, 1996^15^ | Individual counselling and assisted PN | A ~10-20-minute one-to-one counselling session with trained staff. Sessions included HE about STDs, importance of completing treatment and not having sex during the treatment period, and why and how they should inform sex partners. Partners’ details were registered. ICs received identifiable contact slips to give to partners which had brief information on the importance of seeking health care even if asymptomatic and displayed the infection, so health staff knew what to treat. Partners were requested to bring the contact slips when they came to clinic. Provider referral offered if IC refused to refer. | 2 weeks | Clinic | Nurses |  | |  |
|  | SOC | No contact slips or counselling. | 2 weeks | Clinic | Nurses |  | |  |
|  |  | *Syphilis studies* |  |  |  |  |  |  |
| Clark, 2018^8^ | Arm 1: web-based PN only + SOC | SOC plus participants made aware of web-based notification resources and supplied with the link. These provided anonymous PN messaging services, and information on testing and treatment available in major metropolitan areas. | 21 days | Clinic | Clinic staff |  |  |  |
|  | Arm 2: referral cards + web-based PN + SOC | SOC plus provided with both the web-based resources and referral cards. | 21 days | Clinic | Clinic staff |  |  |  |
|  | Arm 3: referral cards only + SOC | SOC plus provided with 5 cards to be delivered to recent sexual partners. Each card contained information about syphilis and local site details offering free or low-cost HIV and STI testing services. | 21 days | Clinic | Clinic staff |  |  |  |
|  | SOC | SOC: participants advised about the importance of notifying recent partners of their STI diagnosis and informed about free testing and treatment resources at the study website, as well as at other health centres. Participants in all arms asked to return to clinic in 14-21 days for follow-up evaluation and PN update. | 21 days | Clinic | Clinic staff |  |  |  |
| Lukac, 2021^25^ | Patient initiated PN | Public health (PH) nurses offer ICs education and resources to contact partners. | NR | Community | Clinic staff/participants |  |  |  |
|  | SOC (PH initiated PN) | ICs with >1 notifiable partner chose a PN method for each partner. PH nurses offered to contact notifiable partners. | NR | Community | Clinic staff/participants |  |  |  |
| Parkes-Ratanshi 2020^31^ | Short Message Service (SMS) reminders and notification slip for partner screening | SOC plus participants received weekly SMS reminders to encourage partners to attend the STI clinic for syphilis testing for up to 8 weeks. Participant identification (ID) code number was written on the notification slip which partners were asked to bring with them to the clinic and in the SMS reminders. | 8 weeks | ANC | Healthcare professionals |  |  |  |
|  | Phone call reminders and notification slip for partner screening | SOC plus participants received a weekly phone call from a nurse for up to 8 weeks after initial syphilis diagnosis to remind them to encourage their partners to attend the STI clinic for syphilis testing. The participant ID code number was written on the notification slip which partners were asked to return to clinic and was also given to the participant via the nurse calls. | 8 weeks | ANC | Healthcare professionals |  |  |  |
|  | SOC (notification slip) | PN slip given to pregnant female participants when they received syphilis test results, to give to their sexual partner(s) and encourage them to attend the STI clinic for syphilis management. All participants asked to attend ANC every 4 weeks until delivery. One visit post-partum. Follow-up call or visit if mother did not attend. Mother’s knowledge of partner treatment noted. | 8 weeks | ANC | Healthcare professionals |  |  |  |
| Peterman, 1997^32^ | Provider referral, field notification | DIS notified partners without waiting for the IC to notify them. | 90 days | Community and clinic | DIS |  |  |  |
|  | Provider referral, blood notification | Similar to "field notification," except the DIS had the option of drawing blood in the field if partner seemed unlikely to come to clinic for testing. | 90 days | Community and clinic | DIS |  |  |  |
|  | Contract referral | ICs given 2 days to notify partners. If partners did not come to the clinic for testing within 2 days, the DIS would notify the partners on the third day. | 3 days | STI clinic | DIS |  |  |  |
|  |  | *HIV and syphilis* |  |  |  |  | |  |
| Heumann, 2017^16^ | In-person interviews | Partner services interviews done in-person, either at clinics or in the field. | NR | Clinics and field | DIS |  |  |  |
|  | SOC (telephone interviews) | Partner services telephone interviews conducted. | NR | Clinic | DIS |  |  |  |
|  |  | *Trichomoniasis studies* |  |  |  |  | |  |
| Kissinger, 2006^21^ | BEPR | Women given 4 cards with information for partner and treatment guidelines for providers that would see partners. Partners could present this card at a clinic to help the clinician better treat them. Women with >4 partners given additional booklets. | 2-8 weeks | Clinic and community | Participants and healthcare workers |  | |  |
|  | SOC (standard PR) ** | Women instructed to inform partners that they needed to go to a clinic for STI evaluation and treatment. | 2-8 weeks | Community | Participants |  | |  |
| Schwebke, 2010^34^ | SOC + DIS interview and DIS-assisted PN and treatment | SOC plus confidential interviews during which ICs agreed to refer partners to clinic for treatment. Interviews collected descriptions and locating information for partners. Partners were contacted by phone within 2 days and encouraged to participate in the male substudy (reimbursed). Male partners presenting to clinic as a result of this interaction were consented and treated, urethral and urine specimens were obtained for testing for T. vaginalis, and a brief questionnaire administered. If treatment could not be verified after 2 working days, DIS attempted to notify exposed partners by phone or field visits. Partners unwilling to attend the STD clinic for evaluation were supplied educational messages, delivery of medication, and if they elected to participate in the male substudy, completion of a short questionnaire regarding perceptions of the PN process, and collection of a urine specimen to be used for diagnostic testing for T. vaginalis. | 3 months | Clinic | DIS |  | |  |
|  | SOC (PR) ** | ICs received directly observed treatment with metronidazole and standardised educational messages concerning infection and the need for treatment of sexual partners. ICs given standard messages about partner treatment and asked to tell partners to present to clinic for evaluation and treatment. Partners who presented were offered participation in the male sub study. | 3 months | Clinic | Index patients |  | |  |
| AHI, acute HIV infection; ANC, Antenatal clinic; APN, assisted partner notification; APT, accelerated partner therapy; ART, antiretroviral therapy; BEPR, booklet-enhanced partner referral; CASH, contraception and sexual health; CDCS, communicable diseases control section; CHCT, couples HIV counselling and testing; CHW, community health worker; CT, contact tracing; DIS, disease intervention specialist; ePN, enhanced partner notification; GUM, genitourinary medicine; HCW, healthcare workers; HE, health education; HIV, human immunodeficiency virus; HIVST, HIV self-testing; HTS, HIV Testing Services; IAPN, information-assisted partner notification; IC, index case; ID, identification; InSPOT, web-based partner notification service; NAAT, nucleic acid amplification test; NGU, nongonococcal urethritis; NR, not reported; PH, public health; PHC, public health counsellor; PHO, public health officer; PN, partner notification; PR, partner referral; RNA, ribonucleic acid; SMS, short message service; SOC, standard of care; STD, sexually transmitted disease; STI, sexually transmitted infection; T. vaginalis, Trichomoniasis Vaginalis; URL, uniform resource locator; VCT, voluntary HIV counselling and testing.  * There were 4 arms in total for this study, the two patient-delivered partner therapy (PDPT) arms were not included in this review as this element of the intervention was not thought to be related to CT.  ** There were 3 arms in total for this study, one PDPT arm was not included in this review as this element of the intervention was not thought to be related to CT.  *** There were 3 arms in total for this study, one arm concerning risk reduction counselling was not included in this review as this element of the intervention was not thought to be related to CT. | | | | | |  | |  |

## Table S3. Outcomes reported for interventions to improve contact tracing in sexually transmitted infections

| **First author, date; design** | **Trial arm name** | **Outcome Category** | **Specific outcome** | **Time point** | **Index (I) / contact (C)** | **N** | **Value** | **Estimate of effect** |
| --- | --- | --- | --- | --- | --- | --- | --- | --- |
| Andersen, 1998;^1^ RCT | Home sampling | CD | Cases detected per index case | NR | I | 45 | 0.27 | Mean difference: 0.13 (-0.03 to 0.29) |
|  | Doctor sampling |  |  |  |  | 51 | 0.14 |  |
| Apoola, 2009;^2^ RCT | Urine testing group | TR | Percentage of index patients with ≥1 partner treated within 28 days (%) | 28 days | C | 100 | 59 | p=0.661 |
|  | Swab testing group |  |  |  |  | 100 | 62 |  |
| Brown, 2011;^3^ RCT | Contract referral | CD | Percentage of new HIV diagnoses (% [95% CI]) | NR | C | 94 | 24 [15-33] | NR |
|  | Provider referral |  |  |  |  | 115 | 26 [16-35] |  |
|  | Passive referral |  |  |  |  | 93 | 15 [7-22] |  |
| Chen, 2021;^4^ RCT | Contract referral | CD | Number of referred persons with new HIV diagnoses (number referred per index [95% CI]) | NR | I | 655 | 38 (0.06 [0.04 to 0.08]) | Ratio: 1.9, 95% CI: 1.2 to 3.1 |
|  | Passive referral |  |  |  |  | 1230 | 38 (0.03 [0.02 to 0.04]) |  |
|  | Contract referral | CD | Sensitivity analysis (new HIV diagnoses per index case [95% CI]) | NR | I | 644 | 0.07 (0.03 - 0.13) | Ratio: 2.0, 95% CI: 1.2 to 3.2 |
|  | Passive referral |  |  |  |  | 1202 | 0.03 (0.02 - 0.06) |  |
| Cherutich, 2017; ^5^ cRCT | Immediate | CD | Number of new HIV diagnoses (per index case) | 6 weeks | C | 586 | 136 (0.247) | IRR: 5.0, 95% CI: 3.2 to 7.9 |
|  | Delayed |  |  |  |  | 680 | 28 (0.049) |  |
|  | Immediate | TR | Treatment rates among contacts per index case (n) | 6 weeks | C | 586 | 0.16 (88) | IRR:4.4, 95% CI: 2.6 to 7.4 |
|  | Delayed |  |  |  |  | 680 | 0.033 (19) |  |
|  | Immediate | UC | IPV events as related to partner notification or study procedures | 6 weeks | C | 586 | 1 | NR |
|  | Delayed |  |  |  |  | 680 | 1 |  |
| Chiou, 2015;^6^ RCT | Experimental | CD | Mean number of new syphilis positive partners per index case (SD) | 7-8 weeks | I | 42 | 0.43 (0.48) | Poisson regression (β): 0.766, SE: 0.425, p<0.05 |
|  | Control |  |  |  |  | 42 | 0.19 (0.39) |  |
|  | Experimental | CD | Number of syphilis positive partners in those that tested (%) | 7-8 weeks | C | 78 | 18 (23.08) | IRR: 0.952, 95% CI: 0.41 to 2.19 |
|  | Control |  |  |  |  | 33 | 8 (24.24) |  |
|  | Experimental | CD | Mean number of new HIV positive partners per index case (SD) | 7-8 weeks | I | 42 | 0.74 (0.87) | Poisson regression (β): 0.599, SE: 0.318, p<0.001 |
|  | Control |  |  |  |  | 42 | 0.21 (0.40) |  |
|  | Experimental | CD | Number of syphilis positive partners in those that tested (%) | 7-8 weeks | C | 78 | 31 (39.74) | IRR: 1.457, 95% CI: 0.69 to 3.06 |
|  | Control |  |  |  |  | 33 | 9 (27.27) |  |
| Choko, 2021;^7^ cRCT | HIVST | CD | Number of new HIV positive contacts (%) | 28 days | C | 155 | 13 (8.4) | Risk difference: 4.3%, 95% CI: 2.2 to 6.4;  Adjusted IRR: 1.65, 95% CI: 0.49 to 5.55, p=0.337 |
|  | SOC |  |  |  |  | 209 | 9 (4.3) |  |
|  | HIVST + financial incentive | CD | Number of new HIV positive contacts (%) | 28 days | C | 285 | 32 (11.2) | Risk difference: 6.9%, 95% CI: 3.2 to 9.1;  Adjusted IRR: 3.11, 95% CI: 0.99 to 9.77, p=0.044 |
|  | SOC |  |  |  |  | 209 | 9 (4.3) |  |
|  | HIVST | TR | Number of male partners who started antiretroviral therapy | 28 days | I | 1465 | 0 | NR |
|  | HIVST + financial incentive |  |  |  |  | 1632 | 22 |  |
|  | SOC |  |  |  |  | 1447 | 2 |  |
| Clark, 2018;^8^ RCT (pilot) | All web-based PN (arms 1 + 2) | TR | Treatment rates among contacts (%) | 21 days | I | 179 | 20.8 | OR: 0.88, 95% CI: 0.55 to 1.40 |
|  | Control/SOC |  |  |  |  | 94 | 16.3 |  |
|  | All referral cards (arms 2 + 3) | TR | Treatment rates among contacts (%) | 21 days | I | 181 | 15.2 | OR: 0.92, 95% CI: 0.52 to 1.63 |
|  | Control/SOC |  |  |  |  | 94 | 16.3 |  |
|  | All web-based PN (arms 1 + 2) | TR | Treatment rates among contacts (%) | 21 days | I | 179 | 20.8 | NR |
|  | All referral cards (arms 2 + 3) |  |  |  |  | 181 | 15.2 |  |
| Culbert, 2023;^9^ RCT | Impart assisted partner notification | CD | Partners diagnosed with HIV | 6 weeks | C | 51 | 5 | NR |
|  | Self-tell only notification |  |  |  |  | 66 | 0 |  |
| Dibia, 2024;^10^ OCC | Elicitation box | CD | Newly identified HIV positive partners n (%) | NR | C | 1250 | 76 (6.1) | X^2^=0.72, p=0.13 |
|  | Conventional method/passive referral |  |  |  |  | 2546 | 126 (4.9) |  |
| England, 2005;^11^ OCC | Public health officer (PHO) CT | CD | Contacts testing positive n (% [95% CI]) | 13 months | C | 245 | 38 (15.5 [11.5-20.6) | p=0.2 |
|  | Clinic CT |  |  |  |  | 192 | 13 (7.8 [4.8-12.5]) |  |
| Estcourt, 2012;^12^ nrCT | APTHotline | TR | Partners assumed treated by allocated method n (% [95% CI]) | 6 weeks | C | 135 | 47 (35 [26 to 45]) | p<0.001 |
|  | Routine PN |  |  |  |  | 117 | 13 (11 [6 to 19]) |  |
|  | APTPharmacy | TR | Partners assumed treated by allocated method n (% [95% CI]) | 6 weeks | C | 44 | 15 (34 [22 to 49]) | p<0.001 |
|  | Routine PN |  |  |  |  | 117 | 13 (11 [6 to 19]) |  |
|  | APTHotline | TR | Partners assumed treated by allocated method n (% [95% CI]) | 6 weeks | C | 135 | 47 (35 [26 to 45]) | NR |
|  | APTPharmacy |  |  |  |  | 44 | 15 (34 [22 to 49]) |  |
| Estcourt, 2015, 2016;^13, 36^ RCT (Pilot) | APTHotline | DIP | Reinfection of index cases n (%) | 6 weeks | I | 15 | 0 (0) | NR |
|  | APTPharmacy |  |  |  |  | 10 | 1 (10) |  |
|  | Standard PN |  |  |  |  | 13 | 2 (15) |  |
|  | APTHotline | TR | Treatment rates among contacts n (%) | 6 weeks | C | 111 | 39 (35) | Adjusted OR: 0.64, 95% CI: 0.35 to 1.18 |
|  | Standard PN |  |  |  |  | 102 | 46 (45) |  |
|  | APTPharmacy | TR | Treatment rates among contacts n (%) | 6 weeks | C | 100 | 46 (46) | Adjusted OR: 1.06, 95% CI: 0.78 to 1.45 |
|  | Standard PN |  |  |  |  | 102 | 46 (45) |  |
|  | APTHotline | TR | Treatment rates among contacts n (%) | 6 weeks | C | 111 | 39 (35) | NR |
|  | APTPharmacy |  |  |  |  | 100 | 46 (46) |  |
| Estcourt, 2022, 2024;^14, 37^ cRCT | Accelerated partner therapy | DIP | Reinfection rates among index cases that were tested n (%) | 24 weeks | I | 666 | 31 (4.7) | Adjusted OR: 0.66, 95% CI: 0.41 to 1.04, p=0.071; marginal difference: -2.2%, 95% CI: -4.7 to 0.3 |
|  | Control |  |  |  |  | 800 | 53 (6.6) |  |
|  | Accelerated partner therapy | TR | Number of index cases with at least one contact treated n (%) | 4 weeks | I | 881 | 775 (88.0) | Adjusted OR: 1.27, 95% CI: 0.96 to 1.68, p=0.10; marginal difference: 2.7%, 95% CI: -0.5 to 6.0 |
|  | Control |  |  |  |  | 898 | 760 (84.6) |  |
| Faxelid, 1996;^15^ RCT | Individual counselling and assisted partner notification | TR | Partners per male index that attended treatment (n, %) | 2 weeks | I | 143 | 1.8 (262, 85) | p<0.001 |
|  | Control / usual care |  |  |  |  | 152 | 1.2 (176, 55) |  |
|  | Individual counselling and assisted partner notification | TR | Partners per female index that attended treatment (n, %) | 2 weeks | I | 46 | 0.7 (31, 65) | NR |
|  | Control / usual care |  |  |  |  | 48 | 0.7 (30, 56) |  |
| Heumann, 2017;^16^ OCC | In-person interviews | CD | Number of partners diagnosed with syphilis (partners per index) | NR | I | 682 | 193 (0.28) | Adjusted RR: 0.91, 95% CI: 0.71 to 1.17, p=0.470 |
|  | Telephone interviews |  |  |  |  | 646 | 162 (0.25) |  |
|  | In-person interviews | CD | Number of partners diagnosed with HIV (partners per index) | NR | I | 358 | 36 (0.10) | Adjusted RR: 2.17, 95% CI: 1.04 to 4.50, p=0.039 |
|  | Telephone interviews |  |  |  |  | 489 | 24 (0.05) |  |
|  | In-person interviews | TR | Partners treated for syphilis (partners per index) | NR | I | 682 | 703 (1.03) | Adjusted RR: 1.19, 95% CI: 1.03 to 1.37, p=0.017 |
|  | Telephone interviews |  |  |  |  | 646 | 477 (0.74) |  |
| Hu, 2021;^17^ RCT | Assisted partner notification | CD | Number of sexual partners who provided a documented positive HIV test | 4 months | I | 97 | 10 | p=0.451 |
|  | Passive partner notification (SOC) |  |  |  |  | 90 | 3 |  |
| Katz, 1988;^18^ RCT | Field follow-up | TR | Treatment rates among contacts per index case | 6 months | I | 221 | 0.72 | p<0.001 |
|  | Nursing referral |  |  |  |  | 217 | 0.22 |  |
|  | Interview only | TR | Treatment rates among contacts per index case | 6 months | I | 240 | 0.18 | NR |
|  | Nursing referral |  |  |  |  | 217 | 0.22 |  |
|  | Field follow-up | TR | Treatment rates among contacts per index case | 6 months | I | 221 | 0.72 | p<0.001 |
|  | Interview only |  |  |  |  | 240 | 0.18 |  |
| Kerani, 2011;^19^ RCT | inSPOT (web-based partner notification service) | TR | Mean number of partners per patient treated (95% CI) | 2 weeks | I | 10 | 2.00 (1.50 to 3.65) | Ratio of unadjusted means: 1.02, 95% CI: 0.68 to 1.53 |
|  | Standard partner management |  |  |  |  | 13 | 1.96 (1.47 to 2.62) |  |
| Kissinger, 2006;^21^ RCT | Booklet-enhanced partner referral | DIP | Reinfection rate among index cases (%) | 2-12 weeks | I | 147 | 9 | Crude RR: 1.42, 95% CI: 0.59 to 3.41; similarity between all 3 study arms: p=0.64 |
|  | Standard partner referral |  |  |  |  | 155 | 6.3 |  |
| Kissinger, 2005;^20^ RCT | Booklet-enhanced partner referral | DIP | Reinfection rates among index cases (Total tested positive across groups) | 4 weeks | I | 289 | 14.3 | Adjusted OR: 0.22, 95% CI: 0.11 to 0.44 |
|  | Partner referral |  |  |  |  |  | 42.7 |  |
|  | Booklet-enhanced partner referral | TR | Treatment rates among contacts (%) | 4 weeks | I | 707 | 45.6 | Adjusted OR: 1.66, 95% CI: 1.22 to 2.27, p=0.001 |
|  | Partner Referral |  |  |  |  | 579 | 35.0 |  |
| Landis, 1992;^22^ RCT | Provider referral | CD | Number of cases detected among contacts tested n (%) | NR | C | 36 | 9 (25) | NR |
|  | Patient referral |  |  |  |  | 25 | 5 (20) |  |
|  | Provider referral | CD | Secondary attack rate | NR | I | 157 | 5 | NR |
|  | Patient referral |  |  |  |  | 153 | 2 |  |
| Low, 2006;^23^ RCT | Practice Nurse | TR | Treatment rates among contacts | 6 weeks | C | 119 | 53 | NR |
|  | Genitourinary medicine clinic |  |  |  |  | 87 | 39 |  |
|  | Practice Nurse | TR | Mean contacts treated per case randomised (SD) | 6 weeks | I | 72 | 0.74 (0.6) | Mean difference: 0.16, 95% CI: −0.02 to 0.34, p=0.078 |
|  | Genitourinary medicine clinic |  |  |  |  | 68 | 0.57 (0.6) |  |
| Lugada, 2010;^24^ cRCT | Home-based ART | CD | Case detection rate among contacts tested n (%) | NR | C | 2678 | 189 (7.1) | OR: 2.76, 95% CI: 1.97 to 3.86, p<0.001 |
|  | Clinic-based ART |  |  |  |  | 260 | 45 (17.3) |  |
| Lukac, 2021;^25^ OCC | Patient-initiated partner notification | CD | Number of cases detected | NR | C | 236 | 30 | p=0.271 |
|  | Public health nurse–initiated partner notification |  |  |  |  | 834 | 66 |  |
|  | Patient-initiated partner notification | TR | Treatment rates among contacts (n) | NR | C | 236 | 154 | NR |
|  | Public health nurse–initiated partner notification |  |  |  |  | 834 | 416 |  |
| Luo, 2020; ^26^ Cross-sectional/prospective cohort with concurrent control | Couples’ HIV counselling and testing | CD | Case detection rate among contacts who tested n (%) | 3 months | C | 197 | 34 (21.0) | NR |
|  | Information assisted partner notification |  |  |  |  | 155 | 10 (6.8) |  |
|  | Assisted HIV self-testing |  |  |  |  | 16 | 0 (0) |  |
|  | Patient referral |  |  |  |  | 171 | 30 (19.6) |  |
| Malave, 2008;^27^ OCC | STD-clinic partner notification | CD | Proportion of new HIV diagnoses among those tested (%) | 1 year | I | 206 | 27 | p=0.56 |
|  | Non-STD facilities partner notification |  |  |  |  | 3460 | 22.2 |  |
| Mathews, 2020;^28^ RCT | Enhanced partner notification | UC | Intimate partner violence | 12 months | C | 721 | 8 | IRR: 0.5%, 95% CI: −0.5% to 1.5%, p=0.28 |
|  | Health Education |  |  |  |  | 714 | 4 |  |
|  | Enhanced partner notification | UC | Abandonment | 12 months | C | 721 | 20 | IRR: 1.7%, 95% CI: 0.2% to 3.3%, p=0.02 |
|  | Health Education |  |  |  |  | 714 | 7 |  |
|  | Enhanced partner notification | DIP | Overall disease incidence or prevalence | 12 months | I | 350 | 136 | IRR: 1.0, 95% CI: 0.7 to 1.3, p=0.8 |
|  | Health Education |  |  |  |  | 350 | 141 |  |
| Oh, 1996;^29^ nrCT | Provider referral | TR | Treatment rates among contacts N (%) | 6 weeks | I | 47 | 26 (55.3) | p=0.05 |
|  | Patient referral |  |  |  |  | 61 | 22 (36.1) |  |
| Ostergaard, 2003;^30^ RCT | Home sampling | CD | Number of index women with at least one partner identified with chlamydia n (%) | 4 months | I | 637 | 69 (10.8) | Relative effectiveness: 1.63, 95% CI: 1.1 to 2.3, p=0.01 |
|  | Office sampling |  |  |  |  | 663 | 44 (6.6) |  |
|  | Home sampling | CD | Number of index men with at least one partner identified with chlamydia n (%) | 4 months | I | 257 | 16 (6.2) | Relative effectiveness: 3.3, 95% CI: 1.2 to 9.0, p=0.02 |
|  | Office sampling |  |  |  |  | 269 | 5 (1.9) |  |
| Parkes-Ratanshi, 2020;^31^ RCT | Phone call reminders and notification slip for partner screening | CD | Number of cases detected | 8 weeks / 3 months after end of pregnancy | I | 146 | 36 | p=0.267 |
|  | Notification slip |  |  |  |  | 152 | 27 |  |
|  | SMS reminders and notification slip for partner screening | CD | Number of cases detected | 8 weeks / 3 months after end of pregnancy | I | 144 | 35 | p=0.267 |
|  | Notification slip |  |  |  |  | 152 | 27 |  |
|  | SMS reminders and notification slip for partner screening | CD | Case detection rate | 8 weeks / 3 months after end of pregnancy | I | 144 | 35 | p=0.267 |
|  | Phone call reminders and notification slip for partner screening |  |  |  |  | 146 | 36 |  |
| Peterman, 1997;^32^ RCT | Field notification | CD | Case detection rate per index patient | Variable | C | 742 | 0.18 | NR |
|  | Field testing |  |  |  |  | 638 | 0.18 |  |
|  | Contract referral |  |  |  |  | 586 | 0.2 |  |
|  | Field notification | TR | Treatment rates among contacts per index patient | Variable | C | 742 | 0.61 | NR |
|  | Field testing |  |  |  |  | 638 | 0.62 |  |
|  | Contract referral |  |  |  |  | 586 | 0.67 |  |
| Potterat, 1997;^33^ nrCT | Study group (self-referral) | CD | Case detection rate | NR | I | 93 | 70 | NR |
|  | Control group (standard interview) |  |  |  |  | 94 | 67 |  |
| Schwebke, 2010;^34^ RCT | DIS interview and DIS-assisted partner notification and treatment + SOC | DIP | Reinfection rates among index cases (n) | 1 month | I | 100 | 15 | RR: 1.24, 95% CI: 0.88 to 1.74 |
|  | Partner referral (usual care) |  |  |  |  | 92 | 9 |  |
|  | DIS interview and DIS-assisted partner notification and treatment + usual care | DIP | Reinfection rates among index cases | 3 months | I | 64 | 5 | RR: 1.23, 95% CI: 0.70 to 2.16 |
|  | Partner referral (usual care) |  |  |  |  | 60 | 3 |  |
| Wilson, 2009;^35^ RCT | Enhanced patient referral | CD | Reinfection rates in index cases (%) | 6 months | I | 253 | 6 | p=0.02 |
|  | Standard of care partner notification |  |  |  |  | 263 | 11 |  |
| APT, accelerated partner therapy; CD, case detection; CI, confidence interval; cRCT, cluster randomised controlled trial; CT, contact tracing; DIP, disease incidence or prevalence; DIS, disease intervention specialist; HIV, human immunodeficiency virus; HIVST, HIV self-testing; InSPOT, web-based partner notification service; IPV, intimate partner violence; IRR, incidence rate ratio; NR, not reported; nrCT, non-randomised controlled trial; OCC, observational cohort study with control; OR, odds ratio; PHO, public health officer; PN, partner notification; SD, standard deviation; SE, standard error; TR, treatment rate among contacts; RR, risk ratio; RCT, randomised controlled trial; SOC, standard of care; UC, unintended consequences.  + additional break down of smear positive, smear negative and extra pulmonary TB available from paper.  * N is lower as only a portion of each group (63% SOC and 75% Int) were interviewed directly and TB status ascertained.  ** data for active TB contacts of index patients including extrapulmonary and contacts detected with active smear positive pulmonary TB available from paper. | | | | | | | | |

# References

1. Andersen B, Ostergaard L, Moller JK, Olesen F. Home sampling versus conventional contact tracing for detecting Chlamydia trachomatis infection in male partners of infected women: randomised study. *BMJ (Clinical research ed)* 1998;**316**:350-1. <https://doi.org/10.1136/bmj.316.7128.350>

2. Apoola A, Beardsley J. Does the addition of a urine testing kit to use of contact slips increase the partner notification rates for genital chlamydial infection? *International journal of STD & AIDS* 2009;**20**:775-7. <https://doi.org/10.1258/ijsa.2009.009196>

3. Brown LB, Miller WC, Kamanga G, Nyirenda N, Mmodzi P, Pettifor A*, et al.* HIV partner notification is effective and feasible in sub-Saharan Africa: opportunities for HIV treatment and prevention. *J Acquir Immune Defic Syndr* 2011;**56**:437-42. <https://doi.org/10.1097/qai.0b013e318202bf7d>

4. Chen JS, Matoga M, Pence BW, Powers KA, Maierhofer CN, Jere E*, et al.* A randomized controlled trial evaluating combination detection of HIV in Malawian sexually transmitted infections clinics. *Journal of the International AIDS Society* 2021;**24**:e25701. <https://doi.org/10.1002/jia2.25701>

5. Cherutich P, Golden MR, Wamuti B, Richardson BA, Asbjornsdottir KH, Otieno FA*, et al.* Assisted partner services for HIV in Kenya: a cluster randomised controlled trial. *Lancet HIV* 2017;**4**:e74-e82. <https://doi.org/10.1016/S2352-3018(16)30214-4>

6. Chiou P-Y, Lin L-C, Chen Y-M, Wu S-C, Lew-Ting C-Y, Yen H-W*, et al.* The effects of early multiple-time PN counseling on newly HIV-diagnosed men who have sex with men in Taiwan. *AIDS and behavior* 2015;**19**:1773-81. <https://doi.org/10.1007/s10461-015-1007-0>

7. Choko AT, Fielding K, Johnson CC, Kumwenda MK, Chilongosi R, Baggaley RC*, et al.* Partner-delivered HIV self-test kits with and without financial incentives in antenatal care and index patients with HIV in Malawi: a three-arm, cluster-randomised controlled trial. *Lancet Global Health* 2021;**9**:e977-e88. <https://doi.org/10.1016/S2214-109X(21)00175-3>

8. Clark JL, Segura ER, Oldenburg CE, Salvatierra HJ, Rios J, Perez-Brumer AG*, et al.* Traditional and Web-Based Technologies to Improve Partner Notification Following Syphilis Diagnosis Among Men Who Have Sex With Men in Lima, Peru: Pilot Randomized Controlled Trial. *J Med Internet Res* 2018;**20**:e232. <https://doi.org/10.2196/jmir.9821>

9. Culbert GJ, Levy JA, Steffen AD, Waluyo A, Earnshaw VA, Rahadi A. Findings from a prison-based model of HIV assisted partner notification in Indonesia. *Journal of the International AIDS Society* 2023;**26**. <https://doi.org/10.1002/jia2.26132>

10. Dibia CC, Nwaokoro P, Akpan U, Toyo O, Cartier S, Sanwo O*, et al.* Innovations in Providing HIV Index Testing Services: A Retrospective Evaluation of Partner Elicitation Models in Southern Nigeria. *Glob Health Sci Pract* 2024;**12**. <https://doi.org/10.9745/GHSP-D-24-00013>

11. England DO, Currie MJ, Bowden FJ. An audit of contact tracing for cases of chlamydia in the Australian Capital Territory. *Sexual health* 2005;**2**:255-8. <https://doi.org/10.1071/sh05021>

12. Estcourt C, Sutcliffe L, Cassell J, Mercer CH, Copas A, James L*, et al.* Can we improve partner notification rates through expedited partner therapy in the UK? Findings from an exploratory trial of Accelerated Partner Therapy (APT). *Sexually transmitted infections* 2012;**88**:21-6. <https://doi.org/10.1136/sti.2010.047258>

13. Estcourt CS, Sutcliffe LJ, Copas A, Mercer CH, Roberts TE, Jackson LJ*, et al.* Developing and testing accelerated partner therapy for partner notification for people with genital Chlamydia trachomatis diagnosed in primary care: a pilot randomised controlled trial. *Sexually Transmitted Infections* 2015;**91**:548-54.

14. Estcourt CS, Stirrup O, Copas A, Low N, Mapp F, Saunders J*, et al.* Accelerated partner therapy contact tracing for people with chlamydia (LUSTRUM): a crossover cluster-randomised controlled trial. *Lancet Public Health* 2022;**7**:e853-e65. <https://doi.org/10.1016/S2468-2667(22)00204-3>

15. Faxelid E, Tembo G, Ndulo J, Krantz I. Individual counseling of patients with sexually transmitted diseases. A way to improve partner notification in a Zambian setting? *Sex Transm Dis* 1996;**23**:289-92.

16. Heumann CL, Katz DA, Dombrowski JC, Bennett AB, Manhart LE, Golden MR. Comparison of In-Person Versus Telephone Interviews for Early Syphilis and Human Immunodeficiency Virus Partner Services in King County, Washington (2010-2014). *Sex Transm Dis* 2017;**44**:249-54. <https://doi.org/10.1097/OLQ.0000000000000583>

17. Hu Q-H, Qian H-Z, Li J-M, Leuba SI, Chu Z-X, Turner D*, et al.* Assisted Partner Notification and Uptake of HIV Testing among Men Who Have Sex with Men: A Randomized Controlled Trial in China. *Lancet Reg Health West Pac* 2021;**12**:100171. <https://doi.org/10.1016/j.lanwpc.2021.100171>

18. Katz BP, Danos CS, Quinn TS, Caine V, Jones RB. Efficiency and cost-effectiveness of field follow-up for patients with Chlamydia trachomatis infection in a sexually transmitted diseases clinic. *Sex Transm Dis* 1988;**15**:11-6. <https://doi.org/10.1097/00007435-198801000-00003>

19. Kerani RP, Fleming M, DeYoung B, Golden MR. A randomized, controlled trial of inSPOT and patient-delivered partner therapy for gonorrhea and chlamydial infection among men who have sex with men. *Sex Transm Dis* 2011;**38**:941-6. <https://doi.org/10.1097/OLQ.0b013e318223fcbc>

20. Kissinger P, Mohammed H, Richardson-Alston G, Leichliter JS, Taylor SN, Martin DH*, et al.* Patient-delivered partner treatment for male urethritis: a randomized, controlled trial. *Clin Infect Dis* 2005;**41**:623-9. <https://doi.org/10.1086/432476>

21. Kissinger P, Schmidt N, Mohammed H, Leichliter JS, Gift TL, Meadors B*, et al.* Patient-delivered partner treatment for Trichomonas vaginalis infection: A randomized controlled trial. *Sex Transm Dis* 2006;**33**:445-50. <https://doi.org/10.1097/01.olq.0000204511.84485.4c>

22. Landis SE, Schoenbach VJ, Weber DJ, Mittal M, Krishan B, Lewis K*, et al.* Results of a randomized trial of partner notification in cases of HIV infection in North Carolina. *N Engl J Med* 1992;**326**:101-6. <https://doi.org/10.1056/NEJM199201093260205>

23. Low N, McCarthy A, Roberts TE, Huengsberg M, Sanford E, Sterne JAC*, et al.* Partner notification of chlamydia infection in primary care: randomised controlled trial and analysis of resource use. *BMJ (Clinical research ed)* 2006;**332**:14-9. <https://doi.org/10.1136/bmj.38678.405370.7C>

24. Lugada E, Levin J, Abang B, Mermin J, Mugalanzi E, Namara G*, et al.* Comparison of home and clinic-based HIV testing among household members of persons taking antiretroviral therapy in Uganda: Results from a randomized trial. *J Acquir Immune Defic Syndr* 2010;**55**:245-52. <https://doi.org/10.1097/QAI.0b013e3181e9e069>

25. Lukac CD, Consolacion T, Ryan V, Cumming E, Mercado J, Ford G*, et al.* Population-Level Outcomes of Partner Notification Among Gay, Bisexual, and Other Men Who Report Sex With Men Diagnosed With Infectious Syphilis in British Columbia, Canada. *Sex Transm Dis* 2021;**48**:901-8. <https://doi.org/10.1097/OLQ.0000000000001477>

26. Luo M, Hann K, Zhang G, Pan X, Ma Q, Jiang J*, et al.* HIV testing uptake and yield among sexual partners of HIV-positive men who have sex with men in Zhejiang Province, China, 2014-2016: A cross-sectional pilot study of a choice-based partner tracing and testing package. *PloS one* 2020;**15**:e0232268. <https://doi.org/10.1371/journal.pone.0232268>

27. Malave MC, Shah D, Sackoff JE, Rubin S, Begier EM. Human immunodeficiency virus partner elicitation and notification in new york city: public health does it better. *Sex Transm Dis* 2008;**35**:869-76. <https://doi.org/10.1097/OLQ.0b013e31817d2f82>

28. Mathews C, Lombard C, Kalichman M, Dewing S, Banas E, Dumile S*, et al.* Effects of enhanced STI partner notification counselling and provider-assisted partner services on partner referral and the incidence of STI diagnosis in Cape Town, South Africa: randomised controlled trial. *Sexually transmitted infections* 2021;**97**:38-44. <https://doi.org/10.1136/sextrans-2020-054499>

29. Oh MK, Boker JR, Genuardi FJ, Cloud GA, Reynolds J, Hodgens JB. Sexual contact tracing outcome in adolescent chlamydial and gonococcal cervicitis cases. *J Adolesc Health* 1996;**18**:4-9. <https://doi.org/10.1016/1054-139X(95)00109-6>

30. Østergaard L, Andersen B, Møller JK, Olesen F, Worm AM. Managing partners of people diagnosed with Chlamydia trachomatis: A comparison of two partner testing methods. *Sexually Transmitted Infections* 2003;**79**:358-62. <https://doi.org/10.1136/sti.79.5.358>

31. Parkes-Ratanshi R, Mbazira Kimeze J, Nakku-Joloba E, Hamill MM, Namawejje M, Kiragga A*, et al.* Low male partner attendance after syphilis screening in pregnant women leads to worse birth outcomes: the Syphilis Treatment of Partners (STOP) randomised control trial. *Sexual health* 2020;**17**:214-22. <https://doi.org/10.1071/SH19092>

32. Peterman TA, Toomey KE, Dicker LW, Zaidi AA, Wroten JE, Carolina J. Partner notification for syphilis: a randomized, controlled trial of three approaches. *Sex Transm Dis* 1997;**24**:511-8. <https://doi.org/10.1097/00007435-199710000-00003>

33. Potterat JJ, Rothenberg R. The case finding effectiveness of a self referral system for gonorrhea: a preliminary report. *Am J Public Health* 1977;**67**:174-6. <https://doi.org/10.2105/AJPH.67.2.174>

34. Schwebke JR, Desmond RA. A randomized controlled trial of partner notification methods for prevention of trichomoniasis in women. *Sex Transm Dis* 2010;**37**:392-6. <https://doi.org/10.1097/OLQ.0b013e3181dd1691>

35. Wilson TE, Hogben M, Malka ES, Liddon N, McCormack WM, Rubin SR*, et al.* A randomized controlled trial for reducing risks for sexually transmitted infections through enhanced patient-based partner notification. *Am J Public Health* 2009;**99 Suppl 1**:S104-10. <https://doi.org/10.2105/AJPH.2007.112128>

36. Estcourt C, Sutcliffe L, Mercer CH, Copas A, Saunders J, Roberts TE*, et al.* The Ballseye programme: a mixed-methods programme of research in traditional sexual health and alternative community settings to improve the sexual health of men in the UK. *NIHR Journals Library* 2016; 10.3310/pgfar04200. <https://doi.org/10.3310/pgfar04200>

37. Estcourt CS, Mapp F, Woode Owusu M, Low N, Flowers P, Copas A*, et al.* Improving sexual health through partner notification: the LUSTRUM mixed-methods research Programme including RCT of accelerated partner therapy. *National Institute for Health and Care Research* 2024; 10.3310/TRQW3886. <https://doi.org/10.3310/TRQW3886>
